# A concerted two-prong approach to the *in situ* allosteric regulation of bifunctional catalysis†
†Electronic supplementary information (ESI) available. CCDC 1469900–1469903. For ESI and crystallographic data in CIF or other electronic format see DOI: 10.1039/c6sc01454b



**DOI:** 10.1039/c6sc01454b

**Published:** 2016-07-18

**Authors:** C. Michael McGuirk, Jose Mendez-Arroyo, Andrea I. d'Aquino, Charlotte L. Stern, Yuan Liu, Chad A. Mirkin

**Affiliations:** a Department of Chemistry , International Institute for Nanotechnology , Northwestern University , 2145 Sheridan Road , Evanston , Illinois 60208-3113 , USA . Email: chadnano@northwestern.edu

## Abstract

Herein, we report the reversible *in situ* “on–off” allosteric regulation of hydrogen-bond-donating (HBD)–Lewis base co-catalytic activity *via* a concerted two-prong methodology entailing cooperative acid–base chemistry and a structurally addressable coordination complex. Specifically, a heteroligated Pt(ii) weak-link approach (WLA) tweezer complex containing both a hemilabile squaramide–piperidine-based catalytic ligand and a sodium sulfonate hydrogen-bond-accepting (HBA) ligand was synthesized. Due to the hemilabile nature of the catalyst-containing ligand, the heteroligated complex can be reversibly toggled *in situ* between a flexible, semi-open state and a rigid, fully closed state upon the addition of elemental ion cues. ^1^H NMR spectroscopy titration studies show that in the semi-open state interligand hydrogen-bonding prevents substrate recognition by the squaramide unit, while in the fully closed state ligand–ligand interactions are prevented. This results in a catalytically active closed state, whereas in the semi-open state, when the piperidine tertiary amine is deliberately protonated, no catalytic activity is observed. Reversible interconversion between the active fully closed state and the dormant protonated semi-open state is achieved in the presence of substrate upon the concerted addition and abstraction of both a proton and a coordinating elemental anion. In this work, allosteric regulation of catalytic activity is demonstrated for both the Michael addition of nitroethane to β-nitrostyrene and the ring-opening of l-(–)-lactide. Taken together, this work details a potentially generalizable platform for the “on–off” allosteric regulation of a family of HBD–Lewis base co-catalysts capable of catalyzing a broad scope of reactions, including the living ring-opening polymerization of cyclic esters.

## Abstract 2

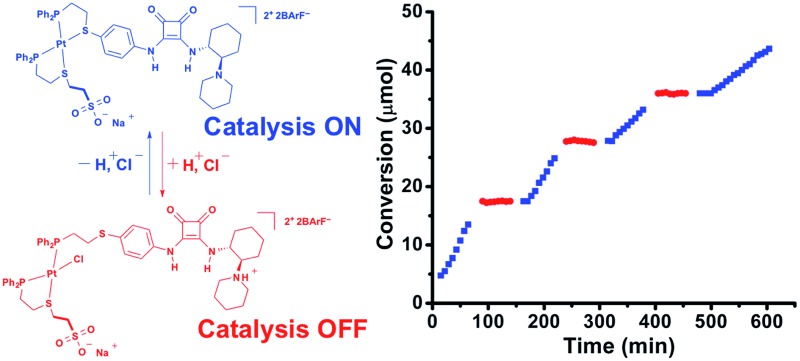
The allosteric regulation of bifunctional catalysis is achieved through the simultaneous use of reversible acid–base and structurally addressable coordination chemistry.

## Introduction

**Scheme 1:**
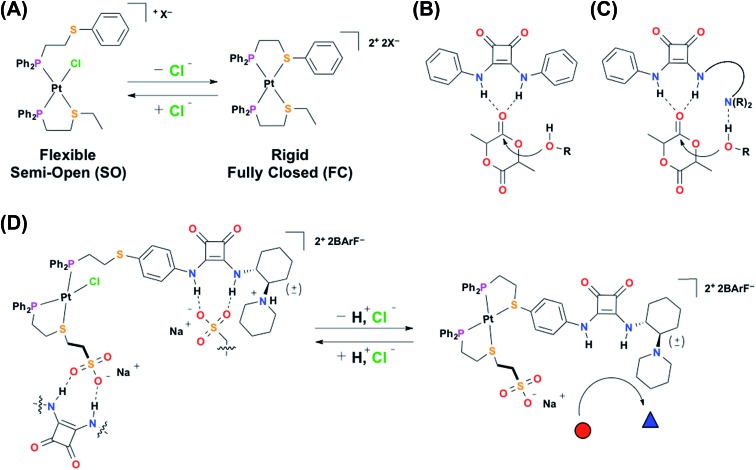
(A) Toggling between the flexible, semi-open (left) and the rigid, fully closed (right) state in a model heteroligated tweezer WLA complex, (B) monofunctional HBD electrophile activation, (C) bifunctional HBD–Lewis base electrophile/nucleophile activation, and (D) the concerted, two-prong approach for the allosteric regulation of HBD–Lewis base bifunctional catalytic activity. The red circle represents catalytic feedstock, and the blue triangle represents catalytic product.

Due to the supramolecular organization of the active site, enzymes are able to catalyze a wide variety of organic transformations with high selectivity and often times impressive rates.^1–5^ Therefore, chemists have sought generalizable strategies for the design of catalytic systems with similar control over local environment.^6–10^ Of the reported approaches, supramolecular coordination chemistry is of particular interest since it allows for the modular and convergent assembly of a broad range of structures with tailorable sizes, shapes, and properties.^11–23^ For example, our group has developed the weak-link approach (WLA) to supramolecular coordination chemistry, which is a powerful tool for the synthesis of multi-component catalytically active complexes that can be reversibly toggled between multiple configurational states *via* coordination-based responses to chemical cues.^24,25^ This structural switching is reminiscent of allosterically regulated enzymes, in which catalytic activity is controlled by changes in the supramolecular environment of the active site caused by recognition events at a chemically orthogonal regulatory site.^26,27^ Structural regulation of WLA complexes is achieved *via* the reversible coordination of chemical effectors (*e.g.*, Cl^–^) to d^8^ metal-based structural nodes (*e.g.*, Rh(i), Pt(ii)) typically coordinated by phosphino-heteroatom (P,X; X = S, O, Se, N) hemilabile ligands.^28^ With this approach, tweezer-like, macrocyclic, and triple-decker multicomponent systems can be readily synthesized and reversibly toggled between a flexible, semi-open (SO) state and a rigid, fully closed (FC) state (Scheme 1A).^29,30^ By synthetically embedding functional moieties into these structures, chemical and physical properties such as catalytic activity, redox potential, and host–guest interactions can be allosterically regulated.^31–34^

Recently, we have shown that the structural switching of a WLA complex can be used to allosterically regulate the catalytic activity of hydrogen-bond-donating (HBD) catalysts through the control of competitive hydrogen-bonding interactions.^35,36^ HBD catalysts activate electrophilic substrates toward nucleophilic attack through cooperative hydrogen-bonding interactions (Scheme 1B), therefore by controlling this ability to associate with a potential substrate, catalytic activity can be regulated. For example, by incorporating a diaryl urea HBD catalyst, which is prone to deleterious hydrogen-bond-driven self-association, into a homoligated WLA complex, we can control catalytic activity based on the coordination mode of a strategically designed structural metal center.^35^ Specifically, the flexible, semi-open state allows for urea self-association, while the rigid, fully closed state minimizes these associative interactions, thus promoting activity. Additionally, we reported that the catalytic activity of a diaryl squaramide HBD catalyst (Scheme 1B) could be reversibly regulated *in situ* in an “on–off” manner by incorporation into a heteroligated WLA construct containing a “regulatory” hydrogen-bond-accepting (HBA) group on the other ligand.^36^ Similarly, the rigid, fully closed state prevents deleterious HB interactions between the ligands and promotes catalysis, while the flexible semi-open state allows such interactions, thus turning off catalysis. In both systems, the HBD catalytic moiety shows significantly improved activity relative to the free catalyst upon incorporation into the supramolecular environment of the rigid fully closed complex. While these reports illustrate the ability of WLA coordination complexes to allosterically regulate HBD catalysis, the small number of reactions that can be catalyzed using monofunctional HBD catalysts hampers the applicability of these systems. In contrast, the closely related family of bifunctional HBD–Lewis base co-catalysts, which are able to cooperatively activate both electrophilic and nucleophilic substrates (Scheme 1C), can promote a large number of organic transformations.^37–41^ Therefore, if one were able to develop chemistry for strategically incorporating a HBD–Lewis base co-catalyst into a WLA complex, in principle, one could create a new family of allosterically regulated catalysts that provide *in situ* control over a broad range of conjugate additions,^42^ Friedel–Crafts reactions,^43^ Nitro–Mannich reactions,^44^ and the ring-opening polymerization of cyclic esters.^45,46^ In this vein, we report a novel two-prong approach for realizing such structures. Notably, this approach entails the simultaneous use of a structurally addressable WLA complex to regulate HBD electrophile activation and acid–base chemistry to control Lewis base nucleophile activation (Scheme 1D, **1H^+^** and **2**).

## Results and discussion

**Scheme 2:**
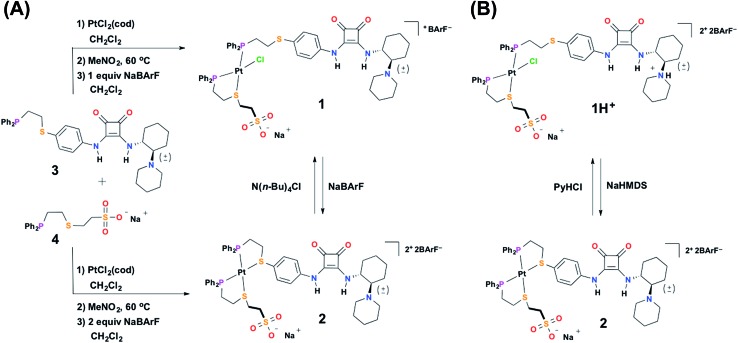
(A) Synthesis of semi-open complex **1** and fully closed complex **2**, and reversible toggling between the two states, and (B) reversible toggling between protonated semi-open **1H^+^** and fully closed **2** *via* concerted chemical cues. PyHCl = pyridinium chloride, NaHMDS = sodium hexamethyldisilazide.

To achieve the allosteric regulation of HBD–Lewis base co-catalytic activity we have designed a heteroligated Pt(ii) tweezer-type complex featuring both a squaramide–tertiary amine (*i.e.*, HBD–Lewis base) co-catalytic ligand (**3**) and a HBA sodium sulfonate-functionalized “regulatory” ligand (**4**) (Scheme 2A). In line with previous systems, we expected that in the fully closed state (**2**) the rigidity, steric bulk, and geometry of full chelation at the Pt(ii) node would prevent the ability of the regulatory HBA sulfonate unit to deleteriously interact with the HBD squaramide, thus allowing the squaramide to activate electrophilic substrates and behave co-catalytically with the tertiary amine. Whereas in the semi-open state (**1**), the flexibility afforded by the non-chelating ligand would allow for interligand interactions, and thus, deter substrate activation and any contribution to catalytic activity by the HBD moiety. In order to simultaneously control catalytic contributions by the Lewis basic tertiary amine, reversible acid–base protonation–deprotonation is used, as the free tertiary amine can behave as a Lewis base, while the conjugate ammonium cannot. Therefore, “on–off” allosteric regulation of this co-catalytic system is realized by simultaneously toggling both the coordination mode of the Pt(ii) structural center and the state of the Lewis base by using acidic and basic salts that allow for *in situ* switching between catalytically active FC **2** and catalytically dormant SO(H^+^) **1H^+^** (Scheme 2B).

**Scheme 3:**
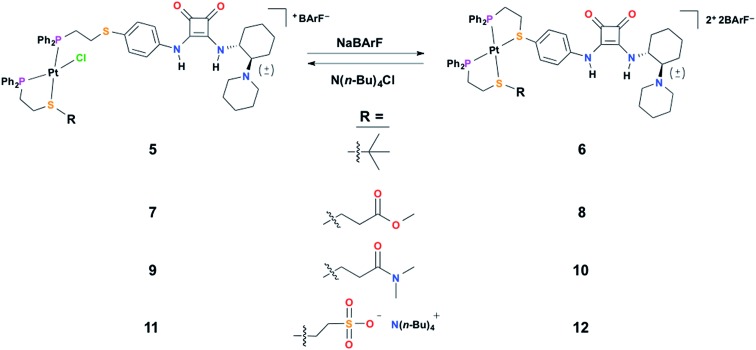
The series of control semi-open (left) and fully closed (right) complexes varying at the regulatory group (R) used in this study.

In order to characterize and understand the structure–function relationships of the multiple configurational states of this system, NMR spectroscopy (*i.e.*, ^1^H, ^31^P{^1^H}, and ^19^F{^1^H}), single crystal X-ray diffraction studies, and computational DFT modeling experiments were performed. The ability of the HBD squaramide unit to recognize a HBA model substrate in the two distinct coordination states was studied by ^1^H NMR spectroscopy substrate titration studies. Using ^1^H NMR spectroscopy, the catalytic activity of the individual states and the ability to reversibly toggle between them *in situ* was extensively studied using the Michael addition of nitroethane to β-nitrostyrene. Importantly, an extensive series of control experiments were performed to aid in the determination of the pathway of allosteric regulation. Throughout this study multiple model constructs, which deviate from **1** and **2** at the HBA regulatory motif, were used as controls (Scheme 3). Finally, the allosteric regulation of the ring-opening of lactide by a primary alcohol was studied.

### Synthesis

#### Ligand **3**

The bifunctional HBD–Lewis base catalytic ligand (**3**) was synthesized through a convergent methodology derived from previous reports on squaramide–piperidine co-catalysis.^47^ Ligand **3** was readily characterized by heteronuclear NMR spectroscopy (*i.e.*, ^1^H, ^31^P{^1^H}) and ESI-MS. Throughout this work, ligand **3** was composed of a racemic mixture of the *trans* diastereomer (*i.e.*, (*R*,*R*) and (*S*,*S*) enantiomers) of the diamino-cyclohexyl unit. Efforts to grow single crystals suitable for X-ray diffraction analysis of **3** were unsuccessful, although we were able to solve the solid-state structure of a related squaramide-functionalized ligand (Fig. S21, see ESI†), which contains a cinchona alkaloid derived moiety in place of the cyclohexyl-piperidine motif found in **3**.^48^

#### Ligand **4**

**Fig. 1:**
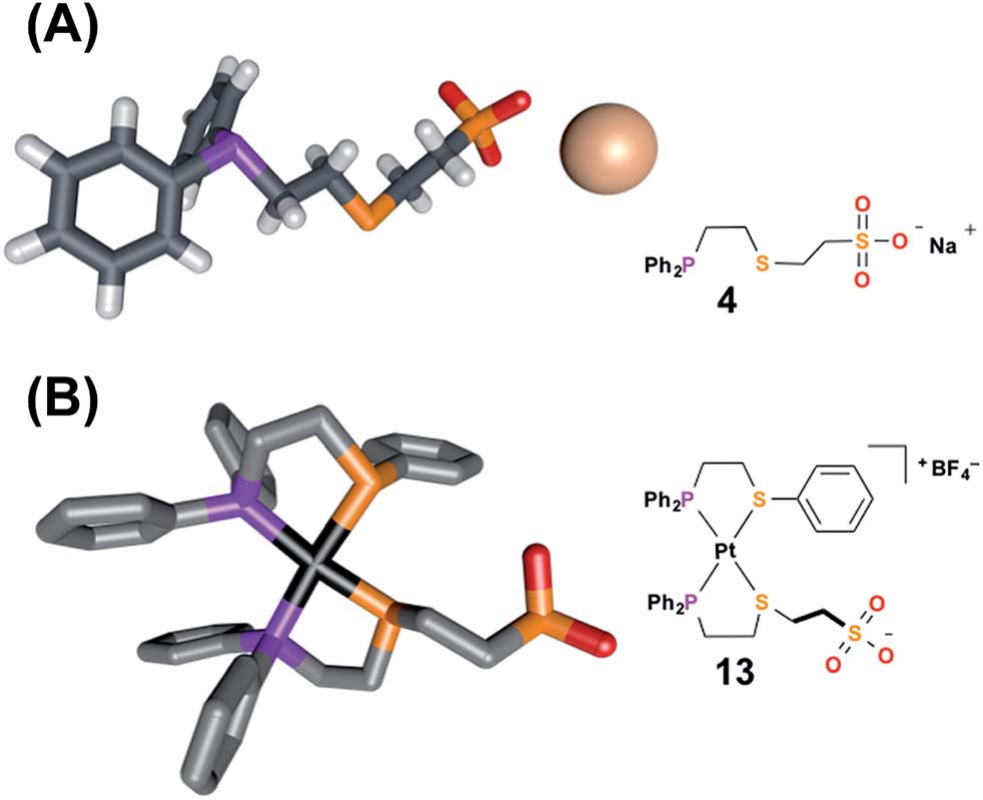
Solid-state X-ray structures of (A) ligand **4** with solvent omitted for clarity, and (B) fully closed complex **13** with outer-sphere anions, hydrogens, and solvent omitted for clarity: C, gray; P, purple; S, orange; O, red; Na, pale pink; Pt, black. Thermal ellipsoid representations can be found in the ESI.†

The HBA regulatory ligand was synthesized in one step *via* the addition of diphenylvinylphosphine to the thiol-terminated aliphatic sodium sulfonate salt.^49^ In addition to characterization by NMR spectroscopy (*i.e.*, ^1^H, ^31^P{^1^H}), single crystals of **4** suitable for X-ray diffraction analysis were grown *via* slow evaporation of an aqueous solution of the ligand (Fig. 1A). In the solid-state structure of **4**, the sodium counter-cation is closely associated to the anionic sulfonate, which proves to be important in controlling ligand–ligand hydrogen-bonding interactions and will be discussed in greater detail later.

#### Semi-open **1**

The synthesis of the tweezer-type complex containing ligands **3** and **4** was afforded *via* an adaptation of our group's previously reported syntheses for heteroligated Pt(ii) complexes.^28,50,51^ Specifically, ligands **3** and **4** and a Pt(ii) precursor salt, dichloro(1,5-cyclooctadiene)platinum (PtCl_2_(cod)), were mixed in 1 : 1 : 1 stoichiometry and stirred in the polar solvent nitromethane at 60 °C overnight (see ESI†). The use of a polar solvent is key, as it screens intermolecular hydrogen-bonding interactions between the ligands, thus allowing the initial complex mixture to afford the thermodynamically favored heteroligated complex.^52^ After removal of nitromethane, the SO complex (**1**) is isolated *via* addition of one equivalent of sodium tetrakis[3,5-bis(trifluoromethyl)phenyl]borate (NaBArF) in CH_2_Cl_2_ (Scheme 2A). The use of the BArF^–^ counter-anion both significantly increases the solubility of these complexes, as well as, prevents the co-precipitation of the Na^+^ counter-cation (belonging to the sulfonate unit) with the outer-sphere counter-anion of the overall complex. In contrast, when tetrafluoroborate (BF_4_^–^) is used as the outer-sphere counter-anion NaBF_4_ precipitates due to its low solubility in CH_2_Cl_2_. ^31^P{^1^H} NMR spectroscopy of **1** displays two resonances: one at 7.60 ppm (*J*_P–Pt_ = 3172 Hz) and the other at 41.84 ppm (*J*_P–Pt_ = 3504 Hz), corresponding to the phosphorous-bound P,S-phenylene ligand (**3**) and the chelated P,S-ethylene ligand (**4**), respectively. These values are in strong agreement with previously reported values for analogous heteroligated P,S-phenyl/P,S-aliphatic Pt(ii) SO model structures.^28,50,51^ Additionally, the broadness of the observed resonances is consistent with previous reports on WLA complexes containing HBD ligands.^35,36^

#### Fully closed **2**

Complex **2** can be synthesized *via* two distinct routes (Scheme 2A): (i) abstraction of the inner-sphere Cl^–^ from SO **1** with one equivalent of NaBArF, or (ii) simultaneous abstraction of both the inner- and outer-sphere chlorides from SO(Cl^–^) *via* the addition of two equivalents of NaBArF. Regardless of the route, the ^31^P{^1^H} NMR spectrum of FC **2** contains two downfield resonances at 45.99 ppm (*J*_P–Pt_ = 3257 Hz) and 45.16 ppm (*J*_P–Pt_ = 3093 Hz), which strongly corresponds with previously reported values for analogous heteroligated P,S-phenyl/P,S-aliphatic Pt(ii) FC model structures.^28,50,51^ Again, the broadness of the observed resonances is consistent with previous reports on WLA complexes containing HBD ligands.^35,36^ Importantly, the observed full chelation of both ligands upon abstraction of both chloride equivalents confirms that the designed inner coordination sphere is robust enough to prevent any undesirable coordination by the tertiary amine moiety of ligand **3**. Additionally, we can reversibly toggle between FC **2** and SO **1** *via* the introduction and subsequent abstraction of Cl^–^ (*i.e.*, *n*-tetrabutylammonium chloride (N(*n*-Bu)_4_Cl) and NaBArF, respectively). N(*n*-Bu)_4_Cl is used because, similar to BArF^–^, the N(*n*-Bu)_4_^+^ cation does not interact significantly with the complex.

To aid the study of the proposed allosteric regulatory pathway a series of similar model complexes were synthesized and are used throughout (Scheme 3). All of the model complexes are structurally analogous to **1** and **2** and they were synthesized and characterized in a similar fashion. Although significant attempts to grow single crystals of complexes containing ligand **3** were unsuccessful, single crystals suitable for X-ray diffraction analysis of FC complex **13** containing a model P,S-phenyl ligand and sulfonate ligand **4** were grown *via* vapor diffusion of pentane into CH_2_Cl_2_ (Fig. 1B). Significantly, since the complex was closed with NaBF_4_, the solid-state structure contains no Na^+^ counter-cation for the sulfonate unit and only one outer-sphere BF_4_^–^, due to the precipitation of NaBF_4_ either during synthesis or during crystal growth. Regardless, not only does the solid-state structure of **13** illustrate the underlying architecture of a FC complex containing the sulfonate ligand, but also shows that even in the absence of a strongly associated counter-cation, the Pt(ii) coordination sphere in the FC state is orthogonal to the anionic sulfonate unit.

### HBA substrate association studies

In order to allosterically regulate electrophile activation by the squaramide moiety (Scheme 1C), and thus its contribution to bifunctional catalysis, the FC state should have full recognition of the HBA substrate, while the SO state has no substrate association. In order to evaluate the recognition of HBA substrate, ^1^H NMR spectroscopy titration studies were performed. Specifically, a series of complexes containing ligand **3**, in both the SO and FC state, were titrated with the strongly HBA small molecule dimethylacetamide (DMA). Recognition of substrate was measured by changes in the ^1^H NMR chemical shifts of the amino-methyl protons of DMA as a function of DMA equivalents added.

**Fig. 2:**
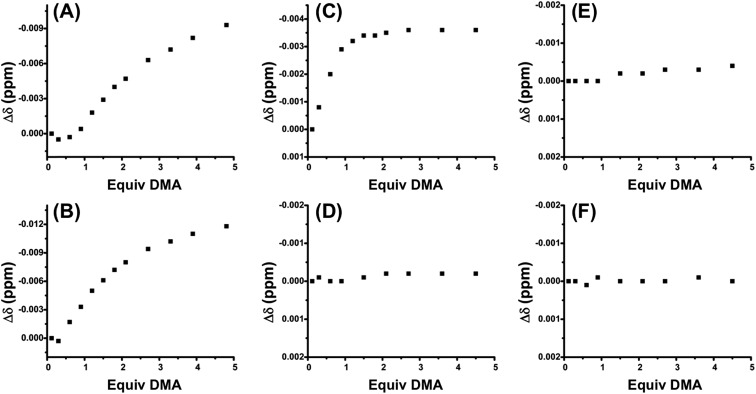
Δ*δ* (ppm) of dimethylacetamide (DMA) *vs.* equivalents of DMA added to (A) fully closed **6** (*K*_a_ = 11 336 ± 46% M^–1^), (B) semi-open **5** (*K*_a_ = 56 428 ± 133% M^–1^), (C) fully closed **2** (*K*_a_ = 75 558 ± 85% M^–1^), (D) semi-open **1**, (E) fully closed **12**, and (F) semi-open **11**. All titrations were performed with a 1.2 mM solution of complex in CD_2_Cl_2_.

Fig. 2A shows the titration of control FC complex **6**, in which the sulfonate-containing ligand **4** is replaced by non-functional P,S-(*t*-butyl) (Scheme 3). The titration of DMA to **6** shows very strong hydrogen-bond-driven association between the squaramide moiety and DMA (*K*_a_ = 11 336 ± 46% M^–1^), which serves as a comparative standard for the functional complexes. As predicted, similar association occurs in the SO state (**5**) (Fig. 2B). For FC complex **8**, containing a methyl ester regulatory unit, strong association with DMA is again observed (*K*_a_ = 21 430 ± 64% M^–1^) (Fig. S1, see ESI†). In the SO state (**7**) association with DMA is observed (*K*_a_ = 910 ± 57% M^–1^), although it is significantly weaker than that observed in the FC state (Fig. S1†). A similar trend is observed for **10** and **9**, in which a dimethyl amide regulatory ligand is used (FC **10** *K*_a_ = 12 043 ± 43% M^–1^, SO **9** *K*_a_ = 935 ± 25% M^–1^) (Fig. S2, see ESI†), thus suggesting the necessity of an even stronger HBA regulatory ligand. In this vein, the titration of FC **2** shows strong association to DMA (*K*_a_ = 75 558 ± 85% M^–1^) (Fig. 2C), whereas, the SO state (**1**) shows no observable recognition of the substrate (Fig. 2D). These results indicate that the sulfonate group is a strong enough HBA moiety to prevent recognition of HBA substrates at the HBD squaramide constituent in the SO state (*i.e.*, **1**). Therefore, in using HBA regulatory ligand **4**, we have developed a two-state system (**1** and **2**) in which substrate recognition by the HBD squaramide can be predictably regulated in an on–off fashion.

With these results, we were curious if the sodium counter-cation, which is tightly associated to the sulfonate anion, played a role in regulating any possible interligand interactions in the FC state. Thus, titrations were performed with the analog complex containing N(*n*-Bu)_4_^+^ as the counter-cation to the sulfonate ligand (**11** and **12**). As seen in Fig. 2E, by switching from Na^+^ to N(*n*-Bu)_4_^+^, HBA substrate recognition in the FC state (**12**) is effectively negated. From these studies multiple points can be inferred: first, although some flexibility exists in the FC structures, ligand–ligand interactions are deterred by the rigidity and geometry induced by full chelation. Second, in order to minimize the hydrogen-bonding-based recognition of a broad scope of HBA substrates in the SO state, an anionic regulatory group is necessary. Third, the sulfonate counter-cation plays a surprisingly significant role in controlling ligand–ligand interactions in the FC state, a point that will be discussed in more detail below.

### DFT calculations

In order to better understand how coordination mode affects the structure and spatial organization of the functional units within the synthesized complexes, as well as help explain the results of the HBA substrate titration studies, we turned to Density Functional Theory (DFT) calculations to generate ground-state, energy minimized models of SO **1** and FC **2**. Calculations were done using PBE:GGA triple-*ζ* potentials, which have previously been used to calculate WLA structures, and when possible, are shown to correlate well with solid-state structures.^32,33,36^ For consistency, all of the calculated structures contain the (*R*,*R*) enantiomer of the diamino-cyclohexyl moiety of **3**.

**Fig. 3:**
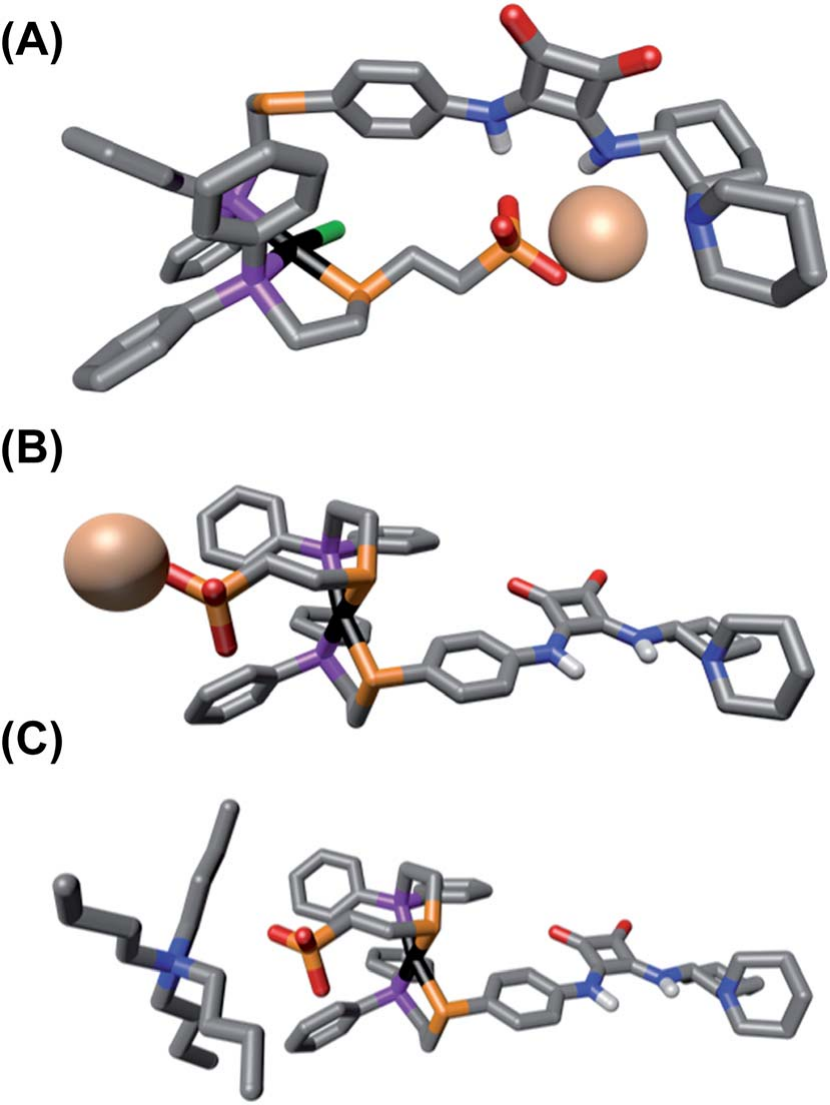
Energy-minimized structures of (A) semi-open **1**, (B) fully closed **2**, and (C) fully closed **12**. All hydrogens, except for on the squaramide unit, are omitted for clarity. C, gray; P, purple; S, orange; Cl, green; O, red; N, blue; Na, pale pink; Pt, black.

As seen in Fig. 3A, the calculated structure of SO **1** illustrates that the combined flexibility of the solely P-bound squaramide-functionalized ligand (**3**) and the ethylene spaced sulfonate-functionalized ligand (**4**) allows strong interligand hydrogen-bonding to occur. While the optimized structure of this single complex shows intramolecular association, we would predict that in solution intermolecular association of the HBD and HBA units is also likely to occur. This strong interaction of the HBD squaramide with the sulfonate anion provides a clear and expected explanation for the observed lack of HBA substrate recognition in the SO coordination mode (Fig. 2D). In contrast to SO **1**, in the optimized structure of FC **2** (Fig. 3B), the rigidity induced by full chelation at the Pt(ii) center prevents intramolecular association, even with the flexibility of the ethylene spacer within the sulfonate ligand. Additionally, in line with previously reported solid-state and calculated FC structures, FC **2** possesses a large dihedral angle between the ligands (C–S–S–C), thus spatially separating the HBD and HBA moieties.^51^ Thus, the rigidity and geometry induced by full chelation at the Pt(ii) center prevents intramolecular association in the FC state.

From the HBA substrate titration study of FC analog **12** (Fig. 2E), containing N(*n*-Bu)_4_^+^, it was clear that the nature of the sulfonate unit's counter-cation is critical to controlling the degree of ligand–ligand interactions in the FC state. The calculated structure of **12** (Fig. 3C) shows that even in the presence of the more loosely associated N(*n*-Bu)_4_^+^, intramolecular hydrogen-bonding association of the ligands is deterred by the relative rigidity, steric profile, and preferred geometry of the FC coordination sphere. Because it was empirically found that changing the counter-cation strongly affects HBA substrate recognition in the FC state (Fig. 2C and E), yet changing the counter-cation to N(*n*-Bu)_4_^+^ does not cause intramolecular ligand–ligand association in the calculated FC structure (Fig. 3B and C), it is reasonable to conclude that the nature of the counter-cation (*i.e.*, Na^+^ *vs.* N(*n*-Bu)_4_^+^) plays a key role in controlling intermolecular association of the sulfonate and squaramide motifs in the FC structure. Therefore, we hypothesize that in FC **2**, containing Na^+^, the combination of effective charge screening by the tightly associated counter-cation and the structural rigidity caused by chelation behaves cooperatively to minimize intermolecular ligand–ligand association, thus allowing for significant HBA substrate recognition. Therefore, in order to achieve maximal catalytic activity in the fully closed state, both architectural and electrostatic effects must be taken into consideration.

### Catalytic activity and *in situ* regulation

**Fig. 4:**
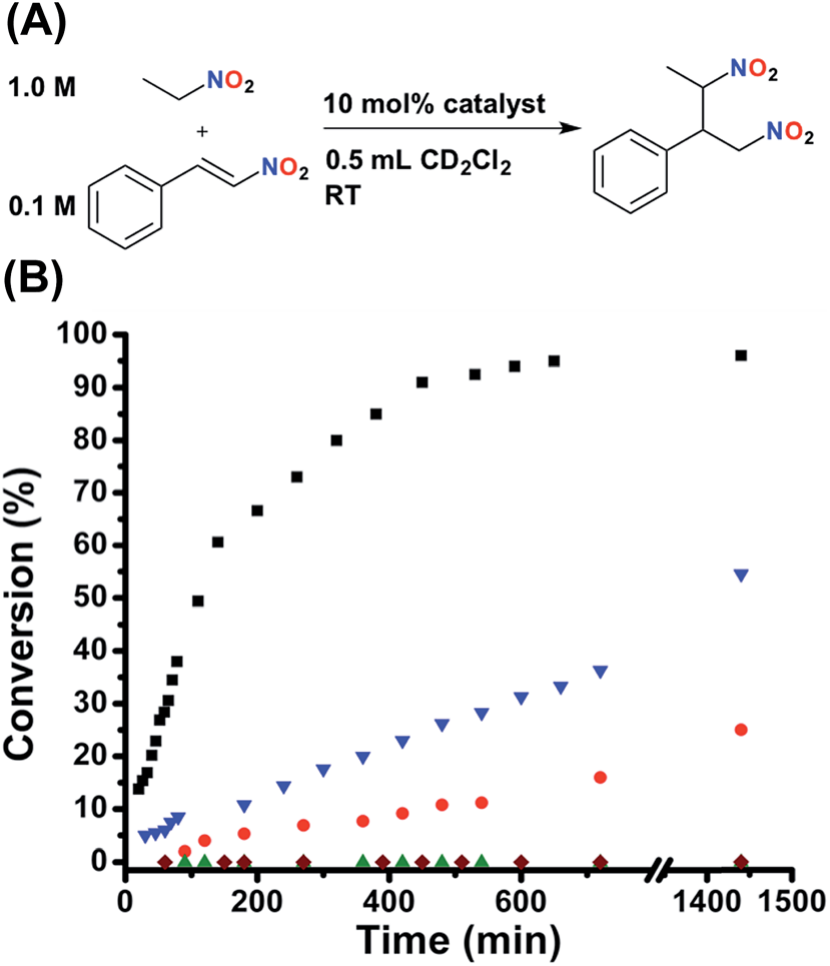
(A) Reaction conditions for the Michael addition between nitroethane and β-nitrostyrene. (B) Fully closed **2** (black squares) shows full conversion, whereas fully closed **12** (N(*n*-Bu)_4_^+^) shows stunted kinetics (blue triangles). Semi-open **1** shows slowed conversion (red circles), but significant residual activity is observed. Protonated semi-open **1H^+^** (green triangles) shows zero conversion, the same as the catalyst free control (maroon diamonds). Reaction progress was monitored by ^1^H NMR spectroscopy with an internal standard. Further details are found in the ESI.†

In order to extensively study the catalytic properties of this system, we decided to use a small molecule reaction that was straightforward to characterize and could be performed without extensive considerations of reaction environment (*i.e.*, moisture and O_2_). Thus, at this stage, catalytic experiments were performed using the Michael addition of nitroethane to β-nitrostyrene to generate (2-nitro-1-(nitromethyl)propyl)benzene (Fig. 4A).^53–55^ This reaction is of particular interest because the reaction product can be readily reduced to realize a 1,3-diamine moiety, a key precursor in many natural product syntheses. The diastereomer distribution of the product was not measured, as it is beyond the scope of this study, although a diastereomeric ratio of approximately 75 : 25 (*syn* : *anti*) was reported for a similar bifunctional catalyst.^55^ As can be seen in Fig. 4B, FC **2** gives the product with nearly quantitative conversion (TOF = 2.69 × 10^–4^ s^–1^). Importantly, control experiments with both FC **6** and SO **5** (P,S-(*t*-butyl)) show very similar kinetic profiles to FC **2** (Fig. S7, see ESI†), demonstrating not only that the presence of the sulfonate in **2** has no effect on activity, but also that confinement of the catalyst to the rigid FC state does not slow catalysis. In fact, functionalizing the ligand onto the Pt(ii) scaffold actually dramatically increases activity, as was determined by measuring the activity of the free ligand (**3**) (Fig. S7, see ESI†). This may be due to the prevention of detrimental self-association upon inclusion into the complex. Additionally, FC model complexes **8** (ester) and **10** (amide) show similar activity to **2**. In contrast to **2**, FC **12** (N(*n*-Bu)_4_^+^) shows significantly deterred activity (Fig. 4B), explicitly demonstrating the nuanced importance of the tightly associated Na^+^ counter-cation to controlling competitive intermolecular ligand–ligand hydrogen-bonding interactions in the FC state. Having established the significant activity of the FC state, the effect of ligand–ligand hydrogen-bonding interactions in the SO state on activity was investigated. Indeed, while SO complexes **7** (ester) and **9** (amide) lack the desired large effect on activity, conversion for SO **1** (sulfonate) is significantly stunted (Fig. 4B), although substantial product formation is still observed. As SO **1** is unable to recognize HBA substrate (Fig. 2D), the observed conversion was attributed to monofunctional nucleophile activation by the Lewis basic tertiary piperidine moiety. This prediction was confirmed when similar conversion, to that of **1**, was observed when the reaction was run solely in the presence of an equimolar loading of free triethylamine (N(Et)_3_) (Fig. S6, see ESI†).

Therefore, in order to achieve a switchable “on–off” two-state system for catalytic activity, a two-prong approach was indeed necessary. Namely, a strategy was needed in which the squaramide moiety was being regulated by competitive hydrogen-bonding interactions while the tertiary amine was simultaneously controlled by reversible protonation/deprotonation. To prove that protonation terminates contributions from the amine, N(Et)_3_ was protonated *in situ via* the addition of one equivalent of pyridinium BArF (PyHBArF) (see Fig. S22† for solid-state structure). Unlike the free amine, the ammonium salt showed no conversion of the Michael addition. Thus, one equivalent of pyridinium chloride (PyHCl) was added to FC **2**, yielding the protonated SO state **1H^+^** (Scheme 2B) (alternatively, addition of N(*n*-Bu)_4_Cl and PyHBArF to **2** or addition of PyHBArF to **1** also yields **1H^+^**). Importantly, **1H^+^** shows zero detectable conversion after 24 h (Fig. 4B), confirming that *via* simultaneously addressing the squaramide and tertiary amine moieties catalytic activity is effectively terminated. In order to confirm that protonation alone does not fully negate activity and that both chemical cues are indeed truly necessary, protonated FC (**2H^+^**) was generated by the addition of PyHBArF to FC **2**. Complex **2H^+^** showed considerable conversion (29%, 24 h, Fig. S9, see ESI†), even when a 50% excess of PyHBArF was added, thus confirming the necessity of the two-effector approach. Importantly, these results confirm that using a concerted two-prong methodology we can regulate the catalytic activity of a bifunctional HBD–Lewis base catalyst in an absolute on–off fashion.

**Fig. 5:**
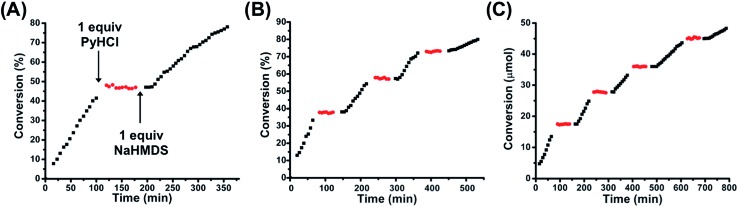
(A) Allosteric regulation of the Michael addition between nitroethane and β-nitrostyrene *via in situ* toggling between fully closed **2** (black squares) and protonated semi-open **1H^+^** (red circles) using one equivalent of PyHCl and one equivalent of NaHMDS. (B) Multiple inversions *in situ* between **2** and **1H^+^** in a closed system (*i.e.*, no additional substrate added after initiation of reaction). (C) Multiple inversions *in situ* between **2** and **1H^+^** with the calculated consumed moles of β-nitrostyrene added before each addition of NaHMDS, thus maintaining approximately *T* = 0 min conditions for each reinitiation of catalytic activity. The same reaction conditions as those found in Fig. 4 were used. Reaction progress was monitored by ^1^H NMR spectroscopy with an internal standard. Further details are found in the ESI.†

Given the binary difference in catalytic activity between FC **2** and SO(H^+^) **1H^+^**, we set out to study the ability to reversibly switch between these two states in the presence of substrate in order to allosterically regulate catalytic activity. As stated above, one equivalent of PyHCl can be used to switch from **2** to **1H^+^**. In order to toggle from **1H^+^** back to FC **2**, one equivalent of sodium hexamethyldisilazide (NaHMDS) was used, as it contains the necessary Na^+^ to abstract the inner-sphere Cl^–^, as well as a non-nucleophilic strong base to readily deprotonate the tertiary amine (Scheme 2B). Importantly, it was empirically determined that the byproducts of toggling between **1H^+^** and **2** (*i.e.*, pyridine and bis(trimethylsilyl)amine) were not basic enough to induce product formation. Using the same reaction conditions described above, FC **2** was used to catalyze the reaction for ∼110 min, after which one equivalent of PyHCl was added to the reaction solution (Fig. 5A). Owing to the rapid *in situ* formation of catalytically inert SO(H^+^) **1H^+^**, conversion was immediately ceased. Following a dormant ∼60 min period in which no conversion occurred, one equivalent of NaHMDS was added, which caused the reformation of FC **2** and the reinitiation of catalytic conversion (Fig. 5A). Therefore, in using straightforward chemical cues for the reversible *in situ* toggling between two configurational states, **2** and **1H^+^**, we have indeed achieved binary on–off allosteric regulation of bifunctional HBD–Lewis base co-catalysis.

### Multiple inversions and recyclability

Thus far, we have established the ability to allosterically regulate the catalytic activity of a HBD–Lewis base co-catalyst. Yet, as can be seen from Fig. 5A, there are apparent differences in the rate of conversion before and after configurational switching. This becomes more apparent when these regions of activity are normalized and superimposed, as in Fig. S12 (see ESI†). Therefore, a thorough study on the effects of multiple configurational inversions on catalytic activity was performed. An *in situ* switching experiment analogous to that found in Fig. 5A was again performed, but in this case configurational inversions were consecutively performed until the catalytic rate significantly slowed. As can be seen in Fig. 5B, three full FC–SO(H^+^)–FC inversions were performed before significant rate loss was observed. In Fig. S13 (see ESI†) the regions of FC **2** catalytic activity have been normalized and superimposed onto one another to more clearly compare their relative kinetics. Interestingly, when these same FC **2** catalysis regions are connected into a single continuous curve, we see that the combined slopes coincide with a plot of uninterrupted catalytic conversion by FC **2** (Fig. S14, see ESI†). Thus, we hypothesized that this apparent loss in activity was primarily induced by substrate (*i.e.*, β-nitrostyrene) depletion. To test this prediction, a similar polyinversion experiment was again performed, but in this case after each region of FC **2** induced conversion and subsequent SO(H^+^) (**1H^+^**) dormancy, the calculated amount of β-nitrostyrene consumed to that point was replenished *via* addition of a concentrated CD_2_Cl_2_ stock solution. At each point of reinitiation of FC **2** catalytic activity, reaction conditions (*e.g.*, substrate loading), besides some light dilution, were approximately the same. Fig. 5C shows that with added substrate, activity is significantly more continuous over a greater number of FC–SO(H^+^)–FC inversions, demonstrating that substrate loss does play a central role in the observed loss of rate in Fig. 5A and B. Although, even with continuous substrate loading, eventual rate loss was still observed (see ESI, Fig. S15,† for normalized/superimposed regions of FC **2** conversion), therefore, it was predicted that the observed rate loss was caused by the byproducts of inversion between the two configurational states (*i.e.*, pyridine and bis(trimethylsilyl)amine) interfering with the HBD–Lewis base catalyst.

In order to probe this effect, a series of control catalysis and titration experiments were performed. First, without the presence of β-nitrostyrene (with only nitroethane present), three full inversions of the complex (FC–SO(H^+^)–FC) were performed. β-Nitrostyrene was then added to initiate the reaction and catalysis was measured, and indeed, we see a significant loss in overall activity (Fig. S16, see ESI†). In order to determine if this observed effect was actually due to pyridine and bis(trimethylsilyl)amine-induced interference, we tested how the direct introduction of three equivalents-worth of byproduct (no configurational inversion) affected catalysis. Thus, to a neutralized solution of three equivalents of PyHCl and NaHMDS (*i.e.*, pyridine, bis(trimethylsilyl)amine, and precipitated NaCl) was added FC **2**, nitroethane, and β-nitrostyrene, and conversion was measured. Fig. S16 (see ESI†) shows that direct addition of multiple equivalents of inversion byproducts induces a similar loss in activity as performing multiple *in situ* FC–SO(H^+^)–FC inversions. This similarity in rate loss strongly implicates byproduct interference as the deleterious mechanism. Additionally, this interference was directly probed with a ^1^H NMR spectroscopy HBA substrate titration study in which DMA recognition was measured after multiple inversions were performed (no substrate present). Fig. S4† shows that, after multiple inversions (FC–SO(H^+^)–FC), DMA recognition is significantly reduced (*K*_a_ = 518 ± 62% M^–1^) owing to byproduct interference, corroborating that the byproducts of complex inversion are a major deleterious factor affecting catalytic activity. Moving forward, the overall *in situ* recyclability of this system could be improved by the exploration of alternative chemical effectors that produce inert byproducts (*i.e.*, are unable to compete with substrate *via* deleterious hydrogen-bonding interactions) or byproducts with appreciably lower boiling points, which could be readily removed under vacuum.

### Ring-opening

**Fig. 6:**
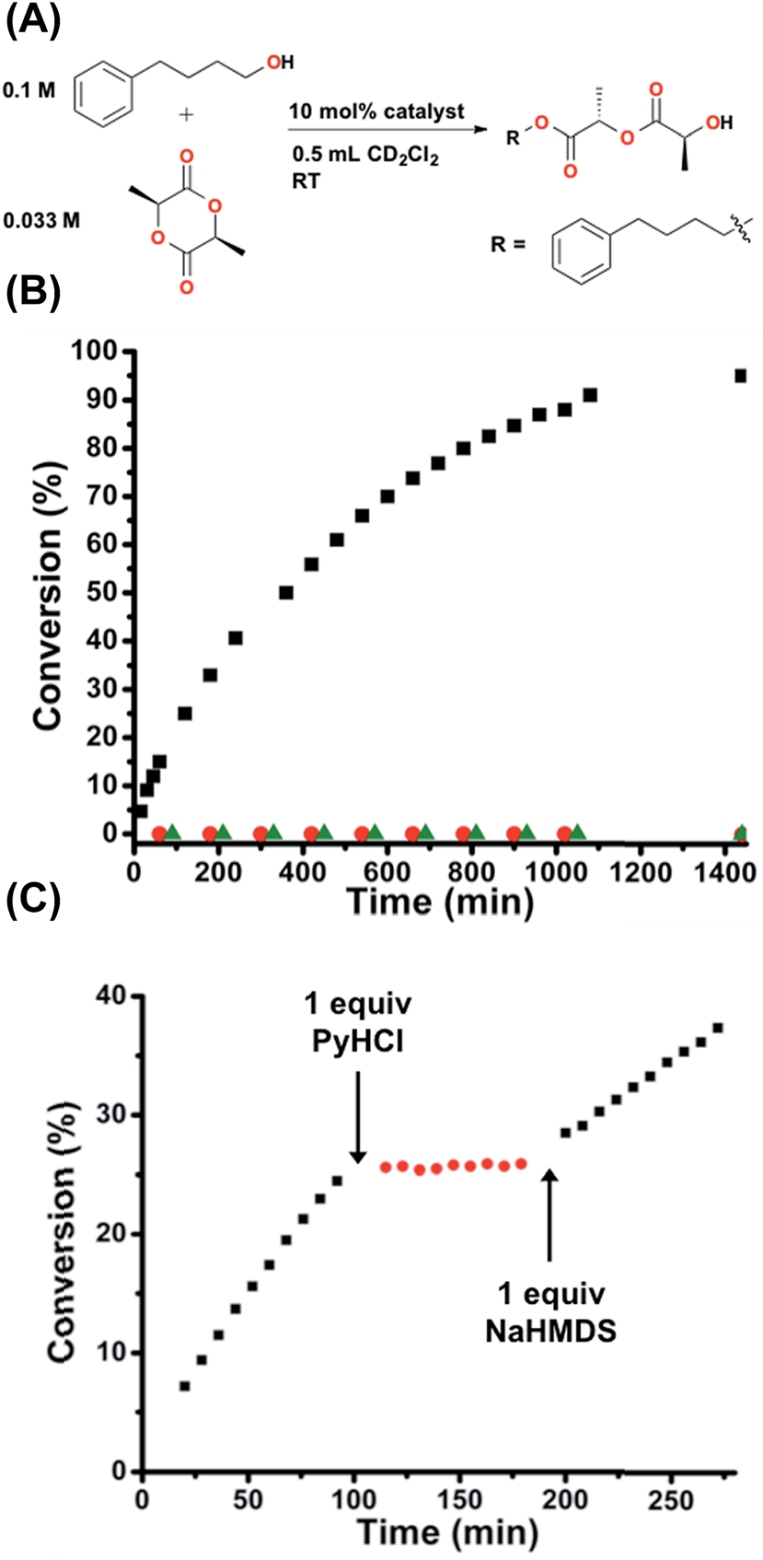
(A) Reaction conditions for the ring-opening of l-(–)-lactide. (B) Fully closed **2** (black squares) shows full conversion, whereas protonated semi-open **1H^+^** (red circles) shows zero conversion, the same as the catalyst free control (green triangles). (C) Allosteric regulation *via in situ* toggling between fully closed **2** (black squares) and protonated semi-open **1H^+^** (red circles). Reaction progress was monitored by ^1^H NMR spectroscopy with an internal standard. Further details found in the ESI.†

In order to study the potential generalizability of the herein detailed regulatory strategy, as well as to validate the possible future application of this approach to the allosteric regulation of ring-opening polymerizations of cyclic esters, we investigated the *in situ* control of the one-step ring-opening of l-(–)-lactide *via* interconversion of **2** and **1H^+^** (Fig. 6A). This reaction was chosen as it not only represents the initial step in the ring-opening polymerization of lactide to form polylactic acid, but also allows for straightforward characterization of a small molecule product.^45^ As seen in Fig. 6B, whereas **1H^+^** is wholly inert towards the ring-opening reaction, FC **2** gives nearly quantitative conversion to the linear product (TOF = 1.15 × 10^–4^ s^–1^), again demonstrating fully orthogonal on–off states of catalytic activity. Therefore, we once again investigated the ability to allosterically regulate catalytic activity *in situ* in the presence of the substrates. Using the same reaction conditions described above and the same *in situ* methodology described for Fig. 5, we are able to allosterically regulate the ring-opening of lactide (Fig. 6C).

## Conclusion

In using a targeted and concerted two-prong approach to reversibly deactivate both underlying functional constituents of a bifunctional system, we have developed a novel and generalizable methodology for the *in situ* allosteric regulation of HBD–Lewis base catalysts. Importantly, we have demonstrated that this strategy not only allows for the highly reversible switching of catalytic activity in a truly “on–off” fashion, but also that this approach can be reliably extended to disparate organic transformations. Additionally, it was shown that incorporation of the catalyst into the WLA architecture improves catalytic activity. Using the underlying design principles elucidated herein, this regulatory approach can potentially be applied to many analogous HBD–Lewis base co-catalysts, which are capable of catalyzing a wide variety of organic transformations. Of particular interest is the application of this approach to the living ring-opening polymerizations of lactide and lactone derivatives for the predictable control of polymer length. This tool has potential applications in the fields of switchable polymer catalysts, stimuli-responsive materials, and molecular sensors.
