# Supplementary material for: A concerted two-prong approach to the in situ allosteric regulation of bifunctional catalysis
Source: Chem Sci. 2016 Jul 18;7(11):6674–83. doi: 10.1039/c6sc01454b (PMC5355828; doi:10.1039/c6sc01454b)
Supplement: Supplementary file 1 [file SC-007-C6SC01454B-s001.pdf]

Supporting Information (SI) to accompany:

A Concerted Two-Prong Approach to the *in Situ* Allosteric Regulation of Bifunctional Catalysis

C. Michael McGuirk, Jose Mendez-Arroyo, Andrea I. d'Aquino, Charlotte L. Stern, Yuan Liu, and Chad. A. Mirkin*

Department of Chemistry and The International Institute for Nanotechnology, Northwestern University, 2145 Sheridan Road, Evanston, Illinois 60208-3113, United States

Table of Contents

General Methods S2

Synthesis S3−21

Computational Calculation Details S23−24

^{1}H NMR Spectroscopy Titration Studies S24−26

Catalysis Experiments S26−37

Single Crystal X−Ray Diffraction Studies S38−45

References S45

NMR Spectra S46−70

General Methods

All phosphino−thioether ligands and precursors were prepared and stored using standard Schlenk line techniques under an inert nitrogen atmosphere, unless noted otherwise. P,S−(phenyl) and P,S−(*t*-butyl) were prepared as previously reported.[1,2] Ligand precursors 1−chloro−2−(diphenylphosphino)ethane and 4−(2−diphenylphosphanylethylthio)phenylamine were prepared as previously reported.[3,4] Dimethyl 3-mercaptopropanamide was prepared as previously reported, except that the product was not purified before moving forward.[5] **S1** (vide infra) was prepared as previously reported.[6] The synthesis, manipulation, and characterization of all Pt(II) complexes were performed under ambient conditions. All solvents used for ligand and complex synthesis were anhydrous grade, purchased from Sigma-Aldrich. Deuterated solvents were purchased from Cambridge Isotope Laboratories. $PtCl_2$(cod) was purchased from Strem Chemicals, Inc. 3,4−dimethoxycyclobut−3−ene−1,2−dione (dimethyl squarate) was purchased from Alfa Aesar. L−(−)−lactide was purchased from TCI Chemicals. All other chemicals were purchased from Aldrich Chemical Co. and used as received unless noted otherwise. NMR spectra were recorded on either a Bruker Avance III 500 or 400 MHz spectrometer. ^{1}H NMR spectra were referenced internally to residual protons in the deuterated solvents (e.g., dichloromethane-d_2 = δ 5.32 ppm). ^{13}C{^{1}H} NMR spectra were referenced internally to carbons in the deuterated solvents (e.g., dichloromethane-d_2 = δ 53.84 ppm). ^{31}P{^{1}H} NMR spectra were referenced to an external 85% $H_3PO_{4(aq)}$ standard (δ 0 ppm). ^{19}F{^{1}H} spectra were reference to an external $CFCl_3$ standard (δ 0 ppm). Electrospray ionization (ESI) mass spectra were recorded on a Bruker AmaZon SL LC−MS instrument in positive ion mode. High-resolution electrospray ionization (ESI) mass spectrometry (HRMS) spectra were recorded on an Agilent 6120 LC-TOF

instrument in positive-ion mode. ^{1}H NMR spectroscopy spectra for catalysis experiments were collected using the "multi_zgvd" command in Topspin 3.2. FT–IR spectra were recorded on a PerkinElmer Spectrum Two FT–IR Spectrometer using films on disposable NaCl cards.

Synthesis

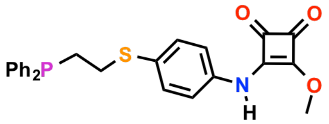

3–(4–(2–diphenylphosphanylethylthio)phenylamino–4–methoxycyclobut–3–ene–1,2–dione. 3,4–dimethoxycyclobut–3–ene–1,2–dione (200.0 mg, 1.4 mmol) and 4–(2–diphenylphosphanylethylthio)phenylamine (523.2 mg, 1.6 mmol) were dissolved in degassed methanol (50 mL) in an oven-dried Schlenk flask and allowed to stir for 16 h. The mixture was filtered over a fritted glass funnel, and the retentate was washed with minimal methanol, then with diethyl ether. The resulting yellow solid was dried under vacuum. (454.3 mg, 72% yield) ^{1}H NMR (400.16 MHz, 25 °C, DMSO-d_6): δ 10.77 (s, 1H), 7.42–7.35 (m, 10H), 7.29 (d, J_{H-H} = 8 Hz, 2H), 7.23 (d, J_{H-H} = 8 Hz, 2H), 4.38 (s, 3H), 2.92 (m, 2H), 2.34 (m, 2H). $^{31}P\{^{1}H\}$ NMR (161.94 MHz, 25 °C, DMSO-d_6): δ −18.46.

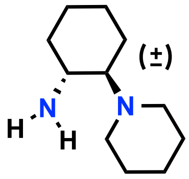

(±)–trans–2–(1–piperidinyl)–cyclohexanamine. The ligand precursor was synthesized using an adaptation of the synthesis reported by Rawal et al.[7] (±)–trans–1,2–diaminocyclohexane

(560.0 mg, 4.9 mmol) and $NaBH(OAc)_3$ (4.16 g, 19.6 mmol) were mixed in dichloroethane (50 mL) at room temperature, then cooled on an ice bath. Glutaraldehyde (50 wt% in H_2O, 0.93 mL, 5.1 mmol) was added dropwise to the stirring mixture, after which the reaction was allowed to stir for 3 h at room temperature. The reaction was slowly quenched with 20 mL of a 6 M aqueous NaOH solution, then extracted with CH_2Cl_2 (3 X 50 mL). The combined organic layers were concentrated under reduced pressure, then reconstituted in CH_2Cl_2 and washed with brine (3 X 50 mL). The organic layer was dried over Na_2SO_4, filtered, then dried under reduced pressure to give a crude brown oily residue containing both the disubstituted byproduct and the desired monosubstituted, as seen by mass spectroscopy. The crude mixture was used in the next step of the ligand synthesis. ESI-MS (*m*/*z*) 183.19 $[M+H]^+$. Found 183.06.

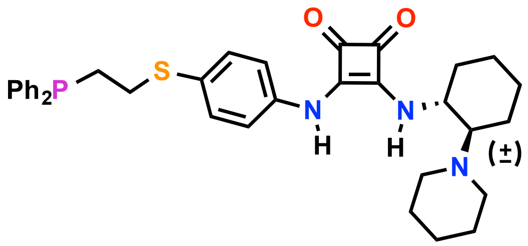

Ligand (***3***). In degassed dichloroethane (20 mL), crude (±)–trans–2–(1–Piperidinyl)–cyclohexanamine (404.7 mg, ~2.2 mmol, calculated 2 equivalents from crude) and 3–(4–(2–diphenylphosphanylethylthio)phenylamino–4–methoxycyclobut–3–ene–1,2–dione (500.0 mg, 1.1 mmol) were mixed and stirred at 65 °C overnight. The reaction mixture was subsequently dried under reduced pressure. Silica-gel column chromatography was performed to give the product as an off-white/brown solid. Column conditions were as follows: The column was packed with EtOAc, and the crude reaction mixture loaded using minimal CH_2Cl_2. The column was eluted with EtOAc, then the solvent system was changed to 2:1 EtOAc:MeOH, with

which the product eluted as a yellow fractions. (230.1 mg, 35% yield) ^{1}H NMR (400.16 MHz, 25 °C, CD_2Cl_2): δ 8.75 (br s, 1H), 7.75−7.27 (m, 15H), 4.00 (br s, 1H), 2.97 (m, 2H), 2.87 (br s, 2H), 2.57 (br s, 2H), 2.46 (br s, 1H), 2.40 (m, 2H), 2.00 (d, J_{H-H} = 10 Hz, 1H), 1.91 (d, J_{H-H} = 10 Hz, 1H), 1.84−1.24 (br m, 12 H). ^{13}C{^{1}H} NMR (100.61 MHz, 25 °C, CD_2Cl_2, major signals): δ 183.99, 169.10, 137.77, 132.73, 132.52, 131.03, 128.81, 128.49, 120.34, 99.98, 68.84, 31.01, 30.78, 28.13, 27.98, 26.30, 25.28, 24.53, 23.12. ^{31}P{^{1}H} NMR (161.94 MHz, 25 °C, CD_2Cl_2): δ −17.65. FT−IR (Film) 3266, 3204, 3076, 2934, 2860, 2812, 1794, 1680, 1606, 1572, 1532, 1452, 1278, 1104, 830, 742, 698 1/cm. ESI-MS (*m*/*z*) 598.27 $[M+H]^+$. Found 598.29. HRMS (*m*/*z*) 598.2657 $[M+H]^+$. Found 598.2648.

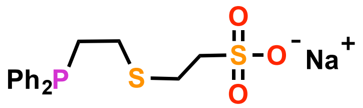

*Ligand (**4**)*. The synthesis for ligand **4** is adapted from the previous synthesis reported by Oehme et al.[8] To degassed H_2O (10 mL) was added a premixed solution of diphenylvinylphosphine (2.12 g, 9.9 mmol) in degassed EtOH (10 mL). Then, sodium 2−mercaptoethanesulfonate (1.63 g, 9.9 mmol) was added directly and the reaction mixture was stirred vigorously overnight. The resulting white precipitate was filtered over a fritted glass funnel and washed with minimal 1:1 H_2O/EtOH and then diethyl ether (2 X 20 mL). In order minimize associated water in the hygroscopic product, the product was dried using a benzene azeotrope with vigorous drying under reduced pressure (3X), followed by drying under high vacuum at 100 °C for two days. The remaining equivalents of water was calculated *via* ^{1}H NMR spectroscopy for each batch and incorporated into the MW for calculating the correct stoichiometry for the synthesis of complexes **1** and **2**. The resulting product was a pristine white

powder. Single crystals suitable for a X–ray diffraction study were grown *via* the slow evaporation of an aqueous solution. (See Figure S19 for single crystal structure) (3.45 g, 81% yield for MW+3H_2O). ^{1}H NMR (400.16 MHz, 25 °C, D_2O): δ 7.54–7.44 (m, 10H), 3.03 (m, 2H), 2.87 (m, 2H), 2.68 (m, 2H), 2.49 (m, 2H). ^{13}C{^{1}H} NMR (125.8 MHz, 25 °C, DMSO-d_6, major signals): δ 138.27, 132.93, 129.09, 51.96, 39.99, 28.05, 27.83, 27.17. ^{31}P{^{1}H} NMR (161.94 MHz, 25 °C, D_2O): δ −19.28. FT–IR (Film) 3636, 3482, 3072, 2926, 1630, 1482, 1434, 1190, 1054, 738, 696 1/cm. HRMS (*m/z*) 399.0230 [M+Na]$^+$. Found 399.0224.

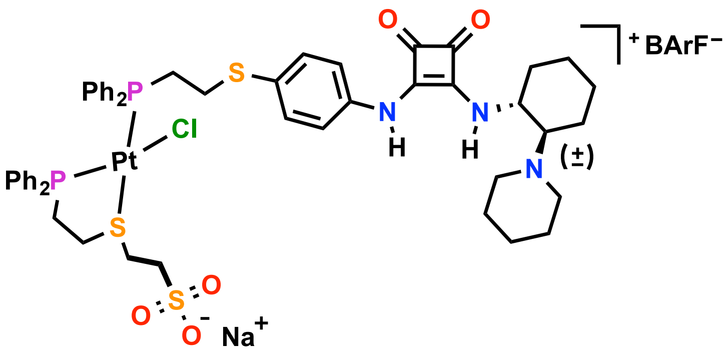

*Semi-open Complex (**1**).* To a solution of $PtCl_2$(cod) (150 mg, 0.40 mmol) in CH_2Cl_2 (15 mL) was added a slurry of **3** (239.1 mg, 0.40 mmol) in CH_2Cl_2 (15 mL), and the mixture was allowed to stir until full dissolution was observed (~20 min). This solution was then transferred to a slurry of **4** (172.2 mg, 0.40 mmol, calculated for 3 equiv H_2O per ligand) in CH_2Cl_2 (15 mL) and stirred until full dissolution was observed (~30 min). Solvent was then minimized (~2 mL) under reduced pressure, the complex mixture was precipitated with hexanes, and the resulting precipitate filtered over a fritted glass funnel. The retentate was washed with hexanes (3 X 10 mL) and dried under vacuum. The resulting powder was added to $MeNO_2$ (30 mL) and stirred at 60 °C overnight, after which solvent was removed under reduced pressure and the complex dried under high vacuum overnight. The resulting dry complex was reconstituted in CH_2Cl_2 (10 mL), 1

equiv of NaBArF was added, and the mixture was stirred for 8 h. The resulting slurry was filtered over Celite on a fritted glass funnel and then washed with dry CH_2Cl_2 (2 X 10 mL). The filtrate was collected, and the solvent was removed under reduced pressure to give **1**. (678.2 mg, 82% yield) ^{1}H NMR (400.16 MHz, 25 °C, CD_2Cl_2): 8.00–6.76 (br m, 36 H), 4.35–3.14 (br m, 8H), 3.02–2.64 (br m, 5 H), 2.54–2.28 (br m, 5H), 2.18–1.71 (br m, 5H), 1.62–1.05 (br m, 10H). ^{13}C{^{1}H} NMR (125.8 MHz, 25 °C, CD_2Cl_2, major signals) δ 162.30, 161.91, 161.51, 161.11, 134.77, 129.21, 128.94, 128.73, 127.82, 125.65, 123.49, 121.32, 117.47, 117.44, 117.41, 117.37, 59.04, 23.75, 19.63, 13.17. ^{31}P{^{1}H} NMR (161.94 MHz, 25 °C, DMSO-d_6): δ 41.84 (br s, J_{P-Pt} = 3504 Hz, 1P), 7.60 (br s, J_{P-Pt} = 3172 Hz, 1P). ^{19}F{^{1}H} NMR (375.49 MHz, 25 °C, CD_2Cl_2): δ –62.72. FT–IR (Film) 3468, 3288, 3196, 2972, 2962, 2884, 1794, 1682, 1614, 1584, 1534, 1438, 1356, 1280, 1130, 888, 838, 714, 682 1/cm. ESI-MS (*m*/*z*) 1256.25 [M–BArF^{-}+3H_2O]$^{+}$. Found 1256.34. HRMS (*m*/*z*) 1203.2248 [M–BArF^{-}]$^{+}$. Found 1203.2261.

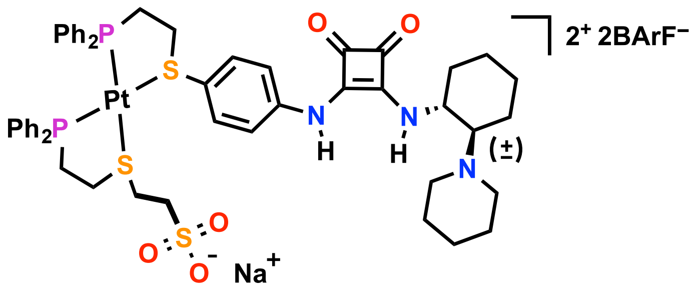

*Fully Closed Complex (**2**)*. The fully closed complex was synthesized *via* two different methods: (a) abstraction of the inner-sphere Cl^{-} of **1** in CH_2Cl_2 with 1 equiv NaBArF, and (b) direct abstraction of both equivalents of Cl^{-} in a synthesis analogous to that described for **1**.

*Method (a)***.** To a solution of **1** (100 mg, 48.3 μmol) in CH_2Cl_2 (20 mL) was added 1 equiv of NaBArF (42.9 mg, 48.3 μmol). The reaction was stirred for 8 h, and the resulting slurry was filtered over Celite on a fritted glass funnel and then washed with dry CH_2Cl_2 (2 X 10 mL).

The filtrate was collected, and the solvent was removed under reduced pressure to give **2**. (132.9 mg, 95% yield)

Method (b). To a solution of $PtCl_2$(cod) (150 mg, 0.40 mmol) in CH_2Cl_2 (15 mL) was added a slurry of **3** (239.1 mg, 0.40 mmol) in CH_2Cl_2 (15 mL), and the mixture was allowed to stir until full dissolution was observed (~20 min). This solution was then transferred to a slurry of **4** (172.2 mg, 0.40 mmol, calculated for 3 equiv H_2O per ligand) in CH_2Cl_2 (15 mL) and stirred until full dissolution was observed (~30 min). Solvent was then minimized (~2 mL) under reduced pressure, the complex mixture was precipitated with hexanes, and the resulting precipitate filtered over a fritted glass funnel. The retentate was washed with hexanes (3 X 10 mL) and dried under vacuum. The resulting powder was added to $MeNO_2$ (30 mL) and stirred at 60 °C overnight, after which solvent was removed under reduced pressure and the complex dried under high vacuum overnight. The resulting dry complex was reconstituted in CH_2Cl_2 (10 mL), 2 equiv of NaBArF were added, and the mixture was stirred for 8 h. The resulting slurry was filtered over Celite on a fritted glass funnel and then washed with dry CH_2Cl_2 (2 X 10 mL). The filtrate was collected, and the solvent was removed under reduced pressure to give **2**. (915.0 mg, 79% yield) ^{1}H NMR (400.16 MHz, 25 °C, CD_2Cl_2): δ 10.04 (br s, 1H), 7.86–7.00 (br m, 48H), 5.79 (br s, 1H), 3.81 (m, 2H), 3.57 (m, 2H), 3.20 (m, 4H), 2.87 (m, 4H), 2.08–1.22 (br m, 20H). ^{13}C{^{1}H} NMR (100.61 MHz, 25 °C, CD_2Cl_2, major signals): δ 162.44, 161.95, 161.45, 160.95, 134.77, 129.33, 128.97, 128.62, 125.91, 123.20, 120.49, 117.47, 31.98, 24.74, 24.24, 23.55, 23.33, 23.18, 22.88, 21.80. ^{31}P{^{1}H} NMR (161.94 MHz, 25 °C, DMSO-d_6): δ 45.99 (br s, J_{P-Pt} = 3257 Hz, 1P), 45.16 (br s, J_{P-Pt} = 3093 Hz, 1P). ^{19}F{^{1}H} NMR (375.49 MHz, 25 °C, CD_2Cl_2): δ −62.74. FT−IR (Film) 3444, 3292, 3220, 3080, 2954, 2876, 1796, 1612, 1538, 1440, 1356, 1280,

1128, 888, 838, 714, 682 1/cm. ESI-MS (*m*/*z*) 602.14 [M−2BArF$^−$+2H_2O]$^{2+}$. Found 603.32. HRMS (*m*/*z*) 573.137 [M−2BArF$^−$−Na^++H^+]$^{2+}$. Found 573.138.

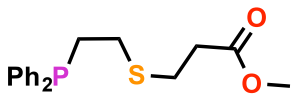

Methyl 3−(2−diphenylphosphanylethylthio)propionate. To dry acetonitrile (50 mL) was added 1−chloro−2−(diphenylphosphino)ethane (200.0 mg, 0.80 mmol), methyl 3-mercaptoproprionate (96.5 mg, 0.80 mmol), and potassium tert-butoxide (98.7 mg, 0.88 mmol), and the solution was heated at reflux for 2 days. The hot solution was filtered through a fritted glass frit, and the solvent was removed from the filtrate under reduced pressure. Silica-gel column chromatography was performed in CH_2Cl_2 to give the product with some residual methyl 3-mercaptoproprionate. To remove the remaining starting material a silica plug was run. The started material was removed with hexanes, followed by product elution with CH_2Cl_2. The resulting product was dried under vacuum. (207.4 mg, 78% yield) ^{1}H NMR (400.16 MHz, 25 °C, CD_2Cl_2): δ 7.42–7.33 (m, 10H), 3.63 (s, 3H), 2.75 (m, 2H), 2.59 (m, 2H), 2.52 (m, 2H), 2.33 (m, 2H). ^{31}P{^{1}H} NMR (161.94 MHz, 25 °C, CD_2Cl_2): δ −17.04. ESI-MS (*m*/*z*) 333.11 [M+H]$^+$. Found 333.13.

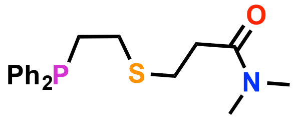

Dimethyl 3−(2−diphenylphosphanylethylthio)propanamide. To dry acetonitrile (50 mL) was added 1−chloro−2−(diphenylphosphino)ethane (200.0 mg, 0.80 mmol), crude dimethyl 3-mercaptopropanamide (106.6 mg, ~0.80 mmol), and potassium tert-butoxide (98.7 mg, 0.88

mmol), and the solution was heated at reflux for 2 days. The hot solution was filtered through a fritted glass frit, and the solvent was removed from the filtrate under reduced pressure. A silica-gel plug was performed to purify the ligand. The plug was loaded and flushed with hexanes, followed by flushing with CH_2Cl_2. The product was eluted off the plug with EtOAc. The resulting product was dried under vacuum. (196.2 mg, 71% yield) ^{1}H NMR (400.16 MHz, 25 °C, $CDCl_3$): δ 7.42–7.33 (m, 10H), 2.95 (s, 3H), 2.94 (s, 3H), 2.83 (m, 2H), 2.59 (m, 2H), 2.51 (m, 2H), 2.33 (m, 2H). ^{13}C{^{1}H} NMR (100.61 MHz, 25 °C, CD_2Cl_2, major signals): δ 170.75, 138.27, 132.56, 128.69, 128.46, 36.86, 35.03, 33.56, 28.62, 28.45, 27.21. ^{31}P{^{1}H} NMR (161.94 MHz, 25 °C, $CDCl_3$): δ –18.77. FT–IR (Film) 3482, 3238, 3054, 2934, 2190, 1976, 1908, 1826, 1652, 1486, 1434, 1406, 1328, 1270, 1180, 1142, 746, 700 1/cm. ESI-MS (*m*/*z*) 346.14 $[M+H]^+$. Found 346.18. HRMS (*m*/*z*) 346.1394 $[M+H^+]^+$. Found 346.1394.

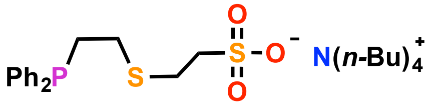

Tetrabutylammonium 2–(2–diphenylphosphanylethylthio)ethanesulfonate. In degassed CH_2Cl_2 ligand **4** (100 mg, 0.27 mmol) and tetrabutylammonium chloride (73.8 mg, 0.27 mmol) were stirred overnight. The resulting cloudy solution was filtered through celite on a fritted glass funnel. The resulting filtrate was dried under reduced pressure to give an oily product. (148.0 mg, 92% yield) ^{1}H NMR (400.16 MHz, 25 °C, CD_2Cl_2): δ 7.40–7.31 (m, 10H), 3.20 (m, 8H), 2.89 (m, 2H), 2.80 (m, 2H), 2.57 (m, 2H), 2.32 (m, 2H), 1.62 (m, 8H), 1.40 (m, 8H), 0.99 (t, $J_{H–H}$ = 7 Hz, 12H). ^{31}P{^{1}H} NMR (161.94 MHz, 25 °C, CD_2Cl_2): δ –17.02.

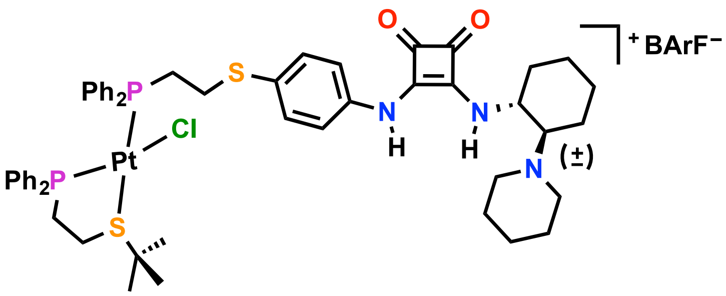

*Semi-open Complex (**5**)*. To a solution of $PtCl_2$(cod) (150 mg, 0.40 mmol) in CH_2Cl_2 (15 mL) was added a solution of P,S–(*t*-butyl) (121.0 mg, 0.40 mmol) in CH_2Cl_2 (15 mL), and the mixture was allowed to stir for ~5 min. This solution was then transferred to a slurry of **3** (239.1 mg, 0.40 mmol) in CH_2Cl_2 (15 mL) and stirred until full dissolution was observed (~30 min). Solvent was then minimized (~2 mL) under reduced pressure, the complex mixture was precipitated with hexanes, and the resulting precipitate filtered over a fritted glass funnel. The retentate was washed with hexanes (3 X 10 mL) and dried under vacuum. The resulting powder was added to $MeNO_2$ (30 mL) and stirred at 60 °C overnight, after which solvent was removed under reduced pressure and the complex dried under high vacuum overnight. The resulting dry complex was reconstituted in CH_2Cl_2 (10 mL), 1 equiv of NaBArF was added, and the mixture was stirred for 8 h. The resulting slurry was filtered over Celite on a fritted glass funnel and then washed with dry CH_2Cl_2 (2 X 10 mL). The filtrate was collected, and the solvent was removed under reduced pressure to give **5**. (695.9 mg, 86%yield) ^{1}H NMR (400.16 MHz, 25 °C, CD_2Cl_2): δ 7.90–7.32 (br m, 38H), 3.90 (br m, 2H), 3.35 (m, 2H), 3.05 (m, 2H), 2.62 (m, 2H), 2.31–1.69 (br m, 20H) 1.30 (s, 9H). $^{13}C\{^1H\}$ NMR (125.8 MHz, 25 °C, CD_2Cl_2, major signals) δ 184.49, 180.09, 170.76, 163.24, 134.10, 133.77, 133.21, 132.99, 132.64, 132.04, 130.05, 129.27, 128.98, 128.55, 119.11, 68.82, 34.21, 31.18, 30.94, 27.98, 26.87, 25.24, 24.95. $^{31}P\{^1H\}$ NMR (161.94 MHz, 25 °C, CD_2Cl_2): δ 42.34 (br s, J_{P-Pt} = 3515 Hz, 1P), 8.10 (br s, J_{P-Pt} = 3182 Hz, 1P).

$^{19}F\{^{1}H\}$ NMR (375.49 MHz, 25 °C, CD_2Cl_2): δ −62.54. FT−IR (Film) 3462, 3156, 2936, 1790, 1682, 1620, 1586, 1534, 1494, 1436, 1104, 838, 710 1/cm. HRMS (*m*/*z*) 1129.3173 [M−BArF$^-$]$^+$. Found 1129.3173.

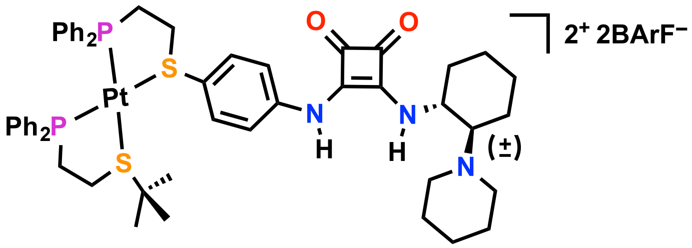

*Fully Closed Complex (**6**)*. The fully closed complex was synthesized *via* two different methods: (a) abstraction of the inner-sphere Cl^- from **5** in CH_2Cl_2 with 1 equiv NaBArF, and (b) direct abstraction of both equivalents of Cl^- in a synthesis analogous to that described for **5**.

*Method (a)***.** To a solution of **5** (100 mg, 50.2 μmol) in CH_2Cl_2 (20 mL) was added 1 equiv of NaBArF (44.4 mg, 50.2 μmol). The reaction was stirred for 8 h, and the resulting slurry was filtered over Celite on a fritted glass funnel and then washed with dry CH_2Cl_2 (2 X 10 mL). The filtrate was collected, and the solvent was removed under reduced pressure to give **6**. (131 mg, 93% yield)

Method (b). To a solution of $PtCl_2$(cod) (150 mg, 0.40 mmol) in CH_2Cl_2 (15 mL) was added a solution of P,S−(*t*-butyl) (121.0 mg, 0.40 mmol) in CH_2Cl_2 (15 mL), and the mixture was allowed to stir for ~5 min. This solution was then transferred to a slurry of **3** (239.1 mg, 0.40 mmol) in CH_2Cl_2 (15 mL) and stirred until full dissolution was observed (~30 min). Solvent was then minimized (~2 mL) under reduced pressure, the complex mixture was precipitated with hexanes, and the resulting precipitate filtered over a fritted glass funnel. The retentate was washed with hexanes (3 X 10 mL) and dried under vacuum. The resulting powder was added to

$MeNO_2$ (30 mL) and stirred at 60 °C overnight, after which solvent was removed under reduced pressure and the complex dried under high vacuum overnight. The resulting dry complex was reconstituted in CH_2Cl_2 (10 mL), 2 equiv of NaBArF were added, and the mixture was stirred for 8 h. The resulting slurry was filtered over Celite on a fritted glass funnel and then washed with dry CH_2Cl_2 (2 X 10 mL). The filtrate was collected, and the solvent was removed under reduced pressure to give **6**. (846.5 mg, 75% yield) ^{1}H NMR (400.16 MHz, 25 °C, CD_2Cl_2): δ 9.99 (br s, 1H), 7.92 (br m, 1H) 7.71–7.32 (br m, 48H), 3.96 (m, 2H), 3.43 (m, 2H), 3.08 (m, 2H), 2.31 (m, 2H), 2.31–1.69 (br m, 20H), 1.30 (s, 9H). ^{13}C{^{1}H} NMR (125.8 MHz, 25 °C, CD_2Cl_2, major signals) δ 184.34, 180.19, 170.47, 162.31, 161.92, 161.52, 161.13, 134.76, 133.88, 133.24, 133.00, 132.11, 129.97, 129.41, 128.99, 128.73, 128.56, 127.81, 125.64, 123.48, 121.31, 119.15, 117.45, 68.78, 34.41, 31.15, 30.86, 30.46, 29.68, 27.98, 26.45, 25.19, 24.84, 24.61, 24.11. ^{31}P{^{1}H} NMR (161.94 MHz, 25 °C, CD_2Cl_2): δ 49.00 (d, $J_{P–Pt}$ = 13 Hz, $J_{P–P}$ = 3250 Hz, 1P), 46.06 (d, $J_{P–Pt}$ = 13 Hz, $J_{P–P}$ = 3105 Hz, 1P). ^{19}F{^{1}H} NMR (375.49 MHz, 25 °C, CD_2Cl_2): δ –62.78. FT–IR (Film) 3468, 3164, 3076, 2940, 1792, 1682, 1618, 1586, 1534, 1438, 1356, 1280, 1130, 840, 714 1/cm. HRMS (*m*/*z*) 1957.4133 [M–BArF$^-$]$^+$. Found 1957.4133.

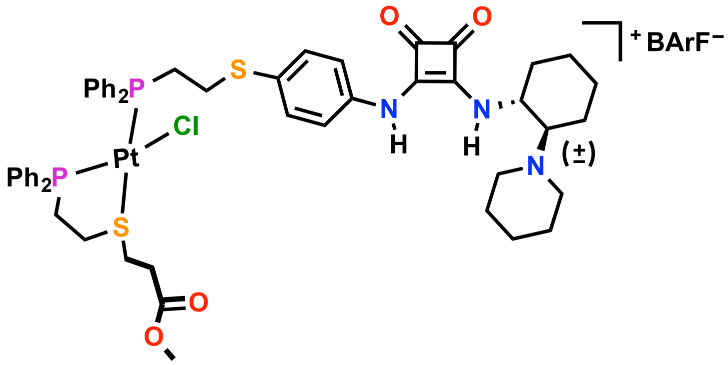

*Semi-open Complex (**7**)*. To a solution of $PtCl_2$(cod) (150 mg, 0.40 mmol) in CH_2Cl_2 (15 mL) was added a solution of methyl 3–(2–diphenylphosphanylethylthio)propionate (133.0 mg,

0.40 mmol) in CH_2Cl_2 (15 mL), and the mixture was allowed to stir for ~5 min. This solution was then transferred to a slurry of **3** (239.1 mg, 0.40 mmol) in CH_2Cl_2 (15 mL) and stirred until full dissolution was observed (~30 min). Solvent was then minimized (~2 mL) under reduced pressure, the complex mixture was precipitated with hexanes, and the resulting precipitate filtered over a fritted glass funnel. The retentate was washed with hexanes (3 X 10 mL) and dried under vacuum. The resulting powder was added to $MeNO_2$ (30 mL) and stirred at 60 °C overnight, after which solvent was removed under reduced pressure and the complex dried under high vacuum overnight. The resulting dry complex was reconstituted in CH_2Cl_2 (10 mL), 1 equiv of NaBArF was added, and the mixture was stirred for 8 h. The resulting slurry was filtered over Celite on a fritted glass funnel and then washed with dry CH_2Cl_2 (2 X 10 mL). The filtrate was collected, and the solvent was removed under reduced pressure to give **7**. (671.9 mg, 83% yield) ^{1}H NMR (400.16 MHz, 25 °C, CD_2Cl_2): δ 7.87–7.34 (br m, 38H), 3.93 (m, 2H), 3.72 (m, 2H), 3.63 (s, 3H), 3.56 (m, 2H), 3.20 (m, 2H), 2.87 (m, 4H), 2.23–1.20 (br m, 20H). ^{13}C{^{1}H} NMR (125.8 MHz, 25 °C, CD_2Cl_2, major signals) δ 184.26, 180.22, 170.87, 133.96, 133.75, 133.34, 132.77, 132.01, 130.08, 129.04, 128.56, 119.11, 68.80, 35.29, 34.38, 33.95, 33.45, 33.02, 31.06, 30.46, 29.67, 24.82. ^{31}P{^{1}H} NMR (161.94 MHz, 25 °C, CD_2Cl_2): δ 41.05 (br s, J_{P-Pt} = 3495 Hz, 1P), 7.95 (br s, J_{P-Pt} = 3156 Hz, 1P). ^{19}F{^{1}H} NMR (375.49 MHz, 25 °C, CD_2Cl_2): δ −63.01. FT−IR (Film) 3456, 3160, 2936, 1792, 1738, 1680, 1620, 1584, 1534, 1492, 1436, 1104, 836, 712 1/cm. HRMS (*m*/*z*) 1159.2915 [M−BArF$^-$]$^+$. Found 1159.2906.

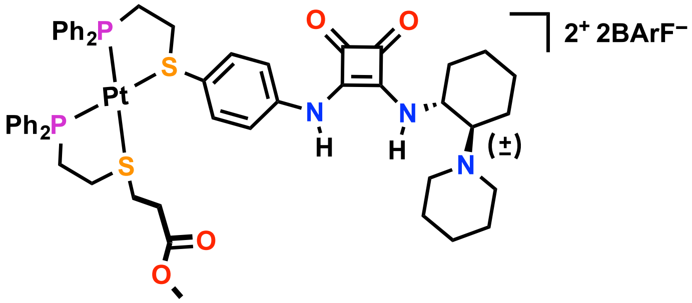

*Fully Closed Complex (**8**)*. The fully closed complex was synthesized *via* two different methods: (a) abstraction of the inner-sphere Cl^- from **7** in CH_2Cl_2 with 1 equiv NaBArF, and (b) direct abstraction of both equivalents of Cl^- in a synthesis analogous to that described for **7**.

*Method (a)***.** To a solution of **7** (100 mg, 49.4 μmol) in CH_2Cl_2 (20 mL) was added 1 equiv of NaBArF (43.8 mg, 49.4 μmol). The reaction was stirred for 8 h, and the resulting slurry was filtered over Celite on a fritted glass funnel and then washed with dry CH_2Cl_2 (2 X 10 mL). The filtrate was collected, and the solvent was removed under reduced pressure to give **8**. (125.4 mg, 89% yield)

Method (b). To a solution of $PtCl_2$(cod) (150 mg, 0.40 mmol) in CH_2Cl_2 (15 mL) was added a solution of methyl 3−(2−diphenylphosphanylethylthio)propionate (133.0 mg, 0.40 mmol) in CH_2Cl_2 (15 mL), and the mixture was allowed to stir for ~5 min. This solution was then transferred to a slurry of **3** (239.1 mg, 0.40 mmol) in CH_2Cl_2 (15 mL) and stirred until full dissolution was observed (~30 min). Solvent was then minimized (~2 mL) under reduced pressure, the complex mixture was precipitated with hexanes, and the resulting precipitate filtered over a fritted glass funnel. The retentate was washed with hexanes (3 X 10 mL) and dried under vacuum. The resulting powder was added to $MeNO_2$ (30 mL) and stirred at 60 °C overnight, after which solvent was removed under reduced pressure and the complex dried under high vacuum overnight. The resulting dry complex was reconstituted in CH_2Cl_2 (10 mL), 2 equiv of NaBArF were added, and the mixture was stirred for 8 h. The resulting slurry was filtered

over Celite on a fritted glass funnel and then washed with dry CH_2Cl_2 (2 X 10 mL). The filtrate was collected, and the solvent was removed under reduced pressure to give **8**. (821.3 mg, 72% yield) ^{1}H NMR (400.16 MHz, 25 °C, CD_2Cl_2): δ 10.06 (br s, 1H), 8.28 (br m, 1H) 7.81–7.33 (br m, 48H), 3.90 (m, 2H), 3.66 (s, 3H), 3.54 (m, 2H), 3.04 (m, 8H), 2.31–1.69 (br m, 20H). ^{13}C{^{1}H} NMR (125.8 MHz, 25 °C, CD_2Cl_2, major signals) δ 166.55, 162.30, 161.91, 161.51, 161.11, 134.77, 133.17, 132.54, 130.43, 130.14, 129.22, 128.99, 128.70, 128.57, 128.31, 127.81, 125.64, 123.48, 121.31, 117.46, 35.14, 34.80, 29.68, 27.98, 24.04, 23.42, 22.33. ^{31}P{^{1}H} NMR (161.94 MHz, 25 °C, CD_2Cl_2): δ 54.53 (d, J_{P-Pt} = 11 Hz, J_{P-P} = 3363 Hz, 1P), 47.24 (d, J_{P-Pt} = 11 Hz, J_{P-P} = 3015 Hz, 1P). ^{19}F{^{1}H} NMR (375.49 MHz, 25 °C, CD_2Cl_2): δ –62.70. FT–IR (Film) 3292, 3202, 3024, 2954, 1796, 1700, 1612, 1594, 1536, 1440, 1356, 1280, 1130, 888, 838, 714, 682 1/cm. HRMS (*m*/*z*) 1987.3875 [M–BArF^{-}]$^{+}$. Found 1987.3871.

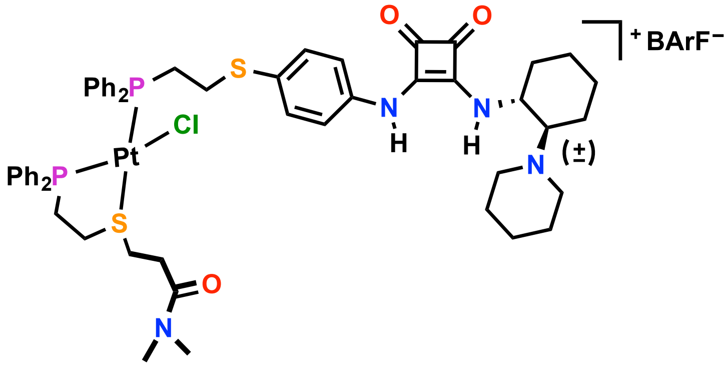

*Semi-open Complex (**9**)*. To a solution of $PtCl_2$(cod) (150 mg, 0.40 mmol) in CH_2Cl_2 (15 mL) was added a solution of dimethyl 3–(2–diphenylphosphanylethylthio)propanamide (138.2 mg, 0.40 mmol) in CH_2Cl_2 (15 mL), and the mixture was allowed to stir for ~5 min. This solution was then transferred to a slurry of **3** (239.1 mg, 0.40 mmol) in CH_2Cl_2 (15 mL) and stirred until full dissolution was observed (~30 min). Solvent was then minimized (~2 mL) under reduced pressure, the complex mixture was precipitated with hexanes, and the resulting precipitate

filtered over a fritted glass funnel. The retentate was washed with hexanes (3 X 10 mL) and dried under vacuum. The resulting powder was added to $MeNO_2$ (30 mL) and stirred at 60 °C overnight, after which solvent was removed under reduced pressure and the complex dried under high vacuum overnight. The resulting dry complex was reconstituted in CH_2Cl_2 (10 mL), 1 equiv of NaBArF was added, and the mixture was stirred for 8 h. The resulting slurry was filtered over Celite on a fritted glass funnel and then washed with dry CH_2Cl_2 (2 X 10 mL). The filtrate was collected, and the solvent was removed under reduced pressure to give **9**. (643.7 mg, 79% yield) ^{1}H NMR (400.16 MHz, 25 °C, CD_2Cl_2): δ 7.80–7.23 (br m, 38H), 3.75 (m, 2H), 3.60 (m, 2H), 3.45 (m, 2H), 3.15 (m, 2H), 3.08 (s, 3H), 2.94 (s, 3H), 2.75 (m, 2H), 2.65 (m, 2H), 2.30–1.15 (br m, 20H). ^{13}C{^{1}H} NMR (125.8 MHz, 25 °C, CD_2Cl_2, major signals) δ 168.99, 162.30, 161.90, 161.51, 161.11, 134.76, 132.87, 129.02, 128.98, 128.94, 128.91, 128.71, 128.68, 128.66, 127.82, 125.65, 123.49, 121.32, 117.47, 117.44, 117.40, 67.73, 59.05, 59.02, 59.00, 36.78, 29.67, 25.56, 23.75, 19.63, 19.61, 13.17. ^{31}P{^{1}H} NMR (161.94 MHz, 25 °C, CD_2Cl_2): δ 41.50 (br s, J_{P-Pt} = 3506 Hz, 1P), 8.02 (br s, J_{P-Pt} = 3165 Hz, 1P). ^{19}F{^{1}H} NMR (375.49 MHz, 25 °C, CD_2Cl_2): δ −62.74. FT–IR (Film) 3442, 3192, 2970, 2940, 2886, 1792, 1612, 1586, 1534, 1438, 1356, 1278, 1126, 888, 838, 714, 682 1/cm. HRMS (*m*/*z*) 1172.3231 $[M{-}BArF^-]^+$. Found 1172.3234.

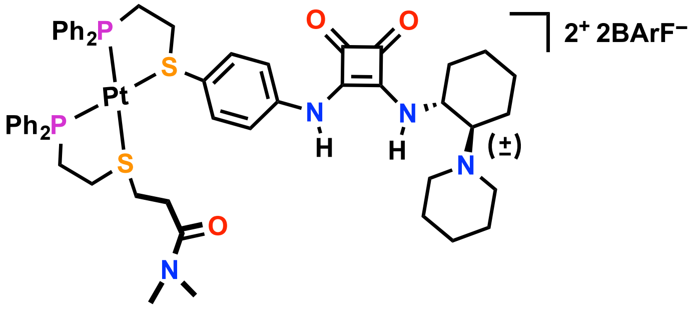

*Fully Closed Complex (**10**)*. The fully closed complex was synthesized *via* two different methods: (a) abstraction of the inner-sphere Cl^- from **9** in CH_2Cl_2 with 1 equiv NaBArF, and (b) direct abstraction of both equivalents of Cl^- in a synthesis analogous to that described for **9**.

*Method (a)***.** To a solution of **9** (100 mg, 49.1 μmol) in CH_2Cl_2 (20 mL) was added 1 equiv of NaBArF (43.5 mg, 49.1 μmol). The reaction was stirred for 8 h, and the resulting slurry was filtered over Celite on a fritted glass funnel and then washed with dry CH_2Cl_2 (2 X 10 mL). The filtrate was collected, and the solvent was removed under reduced pressure to give **10**. (128.0 mg, 91% yield)

Method (b). To a solution of $PtCl_2$(cod) (150 mg, 0.40 mmol) in CH_2Cl_2 (15 mL) was added a solution of dimethyl 3–(2–diphenylphosphanylethylthio)propanamide (138.2 mg, 0.40 mmol) in CH_2Cl_2 (15 mL), and the mixture was allowed to stir for ~5 min. This solution was then transferred to a slurry of **3** (239.1 mg, 0.40 mmol) in CH_2Cl_2 (15 mL) and stirred until full dissolution was observed (~30 min). Solvent was then minimized (~2 mL) under reduced pressure, the complex mixture was precipitated with hexanes, and the resulting precipitate filtered over a fritted glass funnel. The retentate was washed with hexanes (3 X 10 mL) and dried under vacuum. The resulting powder was added to $MeNO_2$ (30 mL) and stirred at 60 °C overnight, after which solvent was removed under reduced pressure and the complex dried under high vacuum overnight. The resulting dry complex was reconstituted in CH_2Cl_2 (10 mL), 2 equiv of NaBArF were added, and the mixture was stirred for 8 h. The resulting slurry was filtered over Celite on a fritted glass funnel and then washed with dry CH_2Cl_2 (2 X 10 mL). The filtrate was collected, and the solvent was removed under reduced pressure to give **10**. (825.0 mg, 72% yield) ^1H NMR (400.16 MHz, 25 °C, CD_2Cl_2): δ 9.97 (br s, 1H), 8.28 (br m, 1H) 7.77–7.20 (br m, 48H), 3.70 (m, 2H), 3.56 (m, 2H), 3.42 (m, 2H), 3.20 (m, 2H), 3.04 (s, 3H), 2.97 (s, 3H), 2.72

(m, 2H), 2.61 (m, 2H), 2.35–1.15 (br m, 20H). $^{13}C\{^1H\}$ NMR (125.8 MHz, 25 °C, CD_2Cl_2, major signals) δ 162.29, 161.89, 161.49, 161.10, 134.76, 130.11, 129.20, 128.95, 128.70, 127.80, 125.63, 123.47, 121.30, 117.50, 117.47, 117.44, 67.91, 29.68, 25.53. $^{31}P\{^1H\}$ NMR (161.94 MHz, 25 °C, CD_2Cl_2): δ 47.28 (br s, $J_{P–P}$ = 3274 Hz, 1P), 46.38 (br s, $J_{P–P}$ = 3109 Hz, 1P). $^{19}F\{^1H\}$ NMR (375.49 MHz, 25 °C, CD_2Cl_2): δ –62.76. FT–IR (Film) 3298, 3206, 2932, 2866, 1798, 1612, 1536, 1440, 1356, 1280, 1130, 888, 838, 714, 682 1/cm. HRMS (*m*/*z*) 2000.4192 $[M–BArF^-]^+$. Found 2000.4193.

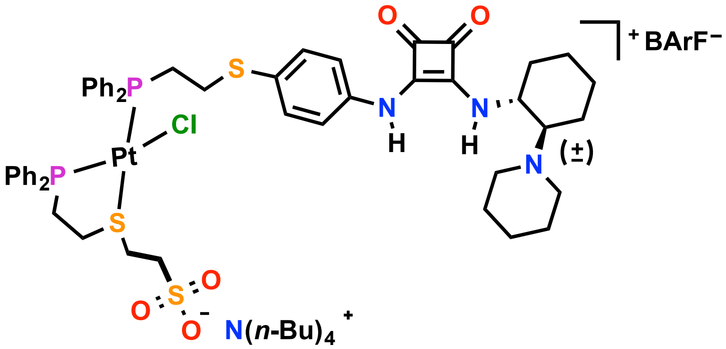

*Semi-open Complex (**11**)*. To a solution of $PtCl_2$(cod) (150 mg, 0.40 mmol) in CH_2Cl_2 (15 mL) was added a solution of tetrabutylammonium 2–(2–diphenylphosphanylethylthio)ethanesulfonate (238.4 mg, 0.40 mmol) in CH_2Cl_2 (15 mL), and the mixture was allowed to stir for ~5 min. This solution was then transferred to a slurry of **3** (239.1 mg, 0.40 mmol) in CH_2Cl_2 (15 mL) and stirred until full dissolution was observed (~30 min). Solvent was then minimized (~2 mL) under reduced pressure, the complex mixture was precipitated with hexanes, and the resulting precipitate filtered over a fritted glass funnel. The retentate was washed with hexanes (3 X 10 mL) and dried under vacuum. The resulting powder was added to $MeNO_2$ (30 mL) and stirred at 60 °C overnight, after which solvent was removed under reduced pressure and the complex dried under high vacuum overnight. The resulting dry

complex was reconstituted in CH_2Cl_2 (10 mL), 1 equiv of NaBArF was added, and the mixture was stirred for 8 h. The resulting slurry was filtered over Celite on a fritted glass funnel and then washed with dry CH_2Cl_2 (2 X 10 mL). The filtrate was collected, and the solvent was removed under reduced pressure to give **11**. (723.1 mg, 79% yield) ^{1}H NMR (400.16 MHz, 25 °C, CD_2Cl_2): 8.05–6.85 (br m, 36 H), 4.40–3.43 (br m, 8H), 3.14 (m, 8H), 2.99–2.53 (br m, 5 H), 2.32–2.15 (br m, 5H), 2.01–1.85 (br m, 5H), 1.79–1.64 (br m, 10H), 1.56 (br s, 8H), 1.30 (m, 8H), 0.91 (m, 12H). ^{31}P{^{1}H} NMR (161.94 MHz, 25 °C, DMSO-d_6): δ 40.95 (br s, J_{P-Pt} = 3498 Hz, 1P), 8.15 (br s, J_{P-Pt} = 3156 Hz, 1P). ^{19}F{^{1}H} NMR (375.49 MHz, 25 °C, CD_2Cl_2): δ −62.74

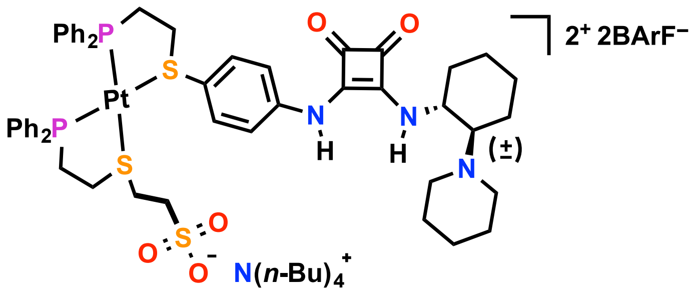

*Fully Closed Complex (**12**)*. The fully closed complex was synthesized *via* two different methods: (a) abstraction of the inner-sphere Cl^- from **11** in CH_2Cl_2 with 1 equiv NaBArF, and (b) direct abstraction of both equivalents of Cl^- in a synthesis analogous to that described for **11**.

*Method (a)***.** To a solution of **11** (100 mg, 43.7 μmol) in CH_2Cl_2 (20 mL) was added 1 equiv of NaBArF (38.7 mg, 43.7 μmol). The reaction was stirred for 8 h, and the resulting slurry was filtered over Celite on a fritted glass funnel and then washed with dry CH_2Cl_2 (2 X 10 mL). The filtrate was collected, and the solvent was removed under reduced pressure to give **12**. (122.5 mg, 90% yield)

Method (b). To a solution of $PtCl_2$(cod) (150 mg, 0.40 mmol) in CH_2Cl_2 (15 mL) was added a solution of tetrabutylammonium 2–(2–diphenylphosphanylethylthio)ethanesulfonate

(238.4 mg, 0.40 mmol) in CH_2Cl_2 (15 mL), and the mixture was allowed to stir for ~5 min. This solution was then transferred to a slurry of **3** (239.1 mg, 0.40 mmol) in CH_2Cl_2 (15 mL) and stirred until full dissolution was observed (~30 min). Solvent was then minimized (~2 mL) under reduced pressure, the complex mixture was precipitated with hexanes, and the resulting precipitate filtered over a fritted glass funnel. The retentate was washed with hexanes (3 X 10 mL) and dried under vacuum. The resulting powder was added to $MeNO_2$ (30 mL) and stirred at 60 °C overnight, after which solvent was removed under reduced pressure and the complex dried under high vacuum overnight. The resulting dry complex was reconstituted in CH_2Cl_2 (10 mL), 2 equiv of NaBArF were added, and the mixture was stirred for 8 h. The resulting slurry was filtered over Celite on a fritted glass funnel and then washed with dry CH_2Cl_2 (2 X 10 mL). The filtrate was collected, and the solvent was removed under reduced pressure to give **12**. (872.2 mg, 70% yield) ^{1}H NMR (400.16 MHz, 25 °C, CD_2Cl_2): δ 10.02 (br s, 1H), 8.05–6.92 (br m, 48H), 3.96 (m, 2H), 3.60 (m, 2H), 3.14 (m, 8H), 3.10 (m, 4H), 2.99 (m, 4H), 2.15–1.20 (br m, 20H) 1.55 (br s, 8H), 1.30 (m, 8H), 0.95 (m, 12H). ^{31}P{^{1}H} NMR (161.94 MHz, 25 °C, DMSO-d_6): δ 46.49 (br s, J_{P-Pt} = 3250 Hz, 1P), 45.94 (br s, J_{P-Pt} = 3110 Hz, 1P). ^{19}F{^{1}H} NMR (375.49 MHz, 25 °C, CD_2Cl_2): δ –62.71.

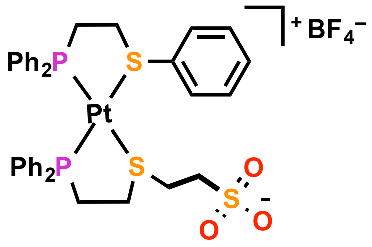

*Fully Closed Complex (**13**).* To a solution of PtCl$_2$(cod) (150 mg, 0.40 mmol) in CH_2Cl_2 (15 mL) was added a solution of P,S–(phenyl) (129.0 mg, 0.40 mmol) in CH_2Cl_2 (15 mL), and the mixture was allowed to stir for ~5 min. This solution was then transferred to a slurry of

ligand **4** (150.5 mg, 0.40 mmol) in CH_2Cl_2 (15 mL) and stirred until full dissolution was observed (~30 min). Solvent was then minimized (~2 mL) under reduced pressure, the complex mixture was precipitated with hexanes, and the resulting precipitate filtered over a fritted glass funnel. The retentate was washed with hexanes (3 X 10 mL) and dried under vacuum. The resulting powder was reconstituted in CH_2Cl_2 and 2 equiv of $NaBF_4$ was added and the solution was stirred overnight. The resulting slurry was filtered over Celite on a fritted glass funnel and then washed with dry CH_2Cl_2 (2 X 10 mL). The filtrate was collected, and the solvent was removed under reduced pressure to give **13**. Single crystals were grown *via* vapor diffusion of pentane into a solution of **13** in CH_2Cl_2. (See Figure S20 for thermal ellipsoid structure). (268.2 mg, 70% yield) ^{1}H NMR (400.16 MHz, 25 °C, CD_2Cl_2): δ 8.05–6.92 (br m, 25H), 3.78 (m, 2H), 3.53 (m, 2H), 3.33 (m, 2H), 3.22 (m, 2H), 2.75 (m, 4H). ^{31}P{^{1}H} NMR (161.94 MHz, 25 °C, DMSO-d_6): δ 50.03 (d, J_{P-Pt} = 13 Hz, J_{P-P} = 3130 Hz, 1P), 45.32 (d, J_{P-Pt} = 13 Hz, J_{P-P} = 3104 Hz, 1P).

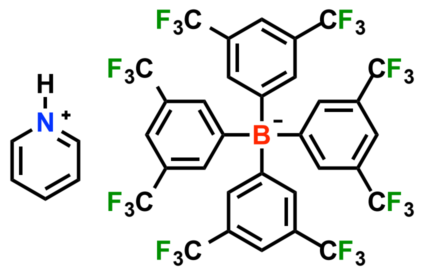

Pyridinium Tetrakis[3,5-bis(trifluoromethyl)phenyl]boron (PyHBArF). In dry, degassed CH_2Cl_2 was mixed pyridinium chloride (PyHCl) (40 mg, 0.35 mmol) and NaBArF (306.8 mg, 0.35 mmol) and stirred overnight. The resulting slurry was filtered over Celite on a fritted glass funnel and then washed with dry CH_2Cl_2 (2 X 10 mL). The filtrate was collected, and the solvent was removed under reduced pressure to give the product. Single crystals were grown *via* slow

evaporation of a CH_2Cl_2 solution of PyHBArF. (See Figure S22 for solid-state structure) (300.5 mg, 91% yield) ^{1}H NMR (400.16 MHz, 25 °C, CD_2Cl_2): δ 8.71–8.67 (m, 3H), 8.16 (m, 2H), 7.72 (s, 8H), 7.58 (s, 4H). ^{19}F{^{1}H} NMR (375.49 MHz, 25 °C, CD_2Cl_2): δ –62.72.

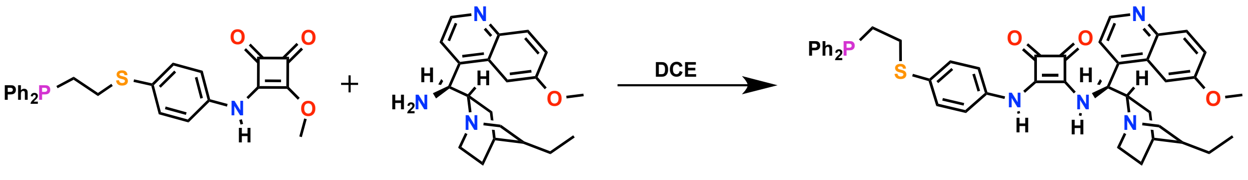

*Ligand (**S2**).*

3–(4–(2–diphenylphosphanylethylthio)phenylamino–4–methoxycyclobut–3–ene–1,2–dione (180.0 mg, 0.40 mmol) and precursor **S1** (107.8 mg, 0.33 mmol) were mixed in degassed dichloroethane (30 mL) and stirred at 65 °C overnight. The reaction mixture was subsequently dried under reduced pressure. Silica-gel column chromatography (EtOAc:MeOH:$NH_4OH_{(aq)}$ 300:70:1) was performed to give **S2** as an off-white/brown solid. (121.5 mg, 41% yield) ^{1}H NMR (400.16 MHz, 25 °C, DMSO-d_6): δ 8.84 (d, $J_{H–H}$ = 4 Hz, 1H), 7.99 (d, $J_{H–H}$ = 9 Hz, 1H), 7.73 (br s, 2H), 7.48 (m, 1H), 7.40 (d, $J_{H–H}$ = 9 Hz, 2H), 7.39–7.30 (m, 10H), 7.19 (d, $J_{H–H}$ = 9 Hz, 2H), 6.13 (br s, 1H), 3.95 (s, 3H), 3.58 (br s, 2H), 3.18, (s, 2H), 2.89 (m, 2H), 2.71 (br s, 1H), 2.31 (m, 2H), 1.80–1.33 (br m, 7H), 0.84 (m, 4H). ^{31}P{^{1}H} NMR (161.94 MHz, 25 °C, DMSO-d_6): δ –18.51. ESI-MS (*m/z*) 741.31 [M+H]$^{+}$. Found 741.28.

Computational

DFT calculations were made using the Amsterdam Density Functional (ADF2014.01) suite on a 64-core Parallel Quantum Solutions (PQS) computational cluster. Geometry optimizations were made without restraint in the ADF GUI using basis sets containing triple-ζ functions with one

polarization function (TZP), and the local density approximations of Becke and Perdew.[9] This was followed by single point calculations using triple-ζ functions with one polarization functions (TZP) and the PBE-hybrid (PBE0) functional by Ernzerhof-Scuseria.[10]

^{1}H NMR Spectroscopy Titration Studies

The general method for titration studies of all fully closed and semi-open complexes was as follows. 1.2 mM NMR solutions (750 μL CD_2Cl_2) of each complex were prepared. A stock solution of dimethylacetamide (DMA) (5.0 μL in 500 μL CD_2Cl_2) was prepared. To the NMR solution of the respective complex was added known equivalents of DMA (2.5 μL = 0.3 equiv) and a ^{1}H NMR spectrum was recorded. Δδ (ppm) of the DMA protons was plotted as a function of DMA equivalents.

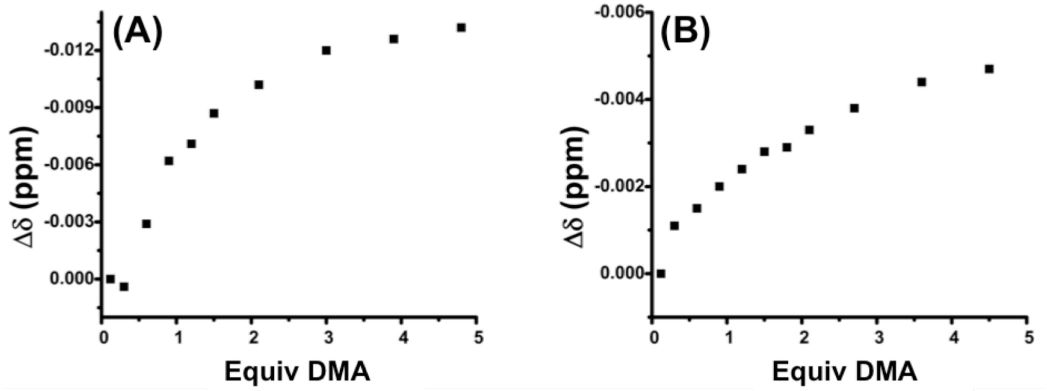

Figure S1. Δδ (ppm) of dimethylacetamide (DMA) vs. equivalents of DMA added to (A) fully closed **8**, (B) semi-open **7**.

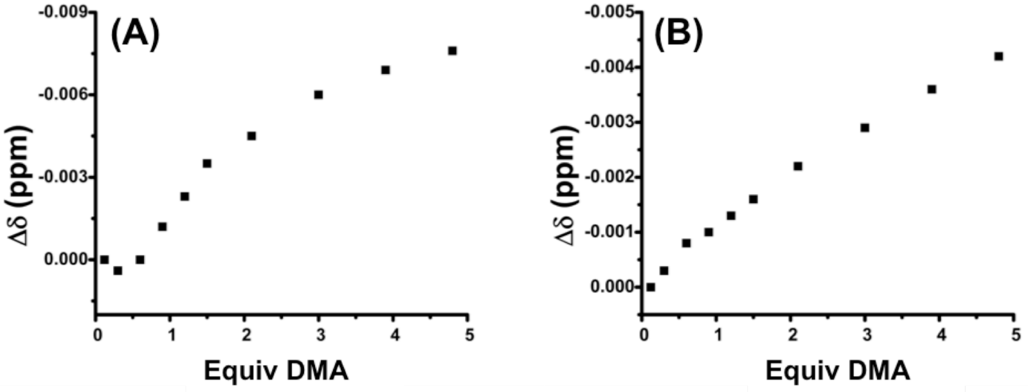

Figure S2. **Δ**δ (ppm) of dimethylacetamide (DMA) vs. equivalents of DMA added to (A) fully closed **10**, (B) semi-open **9**.

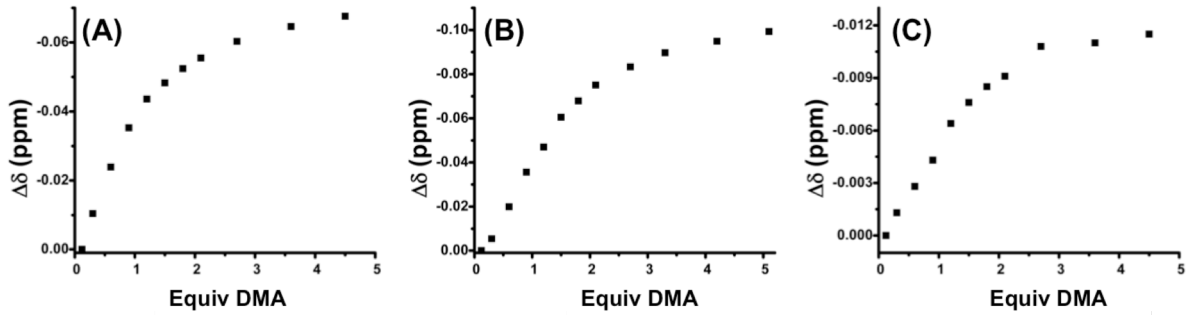

Figure S3. **Δ**δ (ppm) of dimethylacetamide (DMA) vs. equivalents of DMA added to (A) SO(H^+) **1H⁺** (K_a = 6,510 ± 24% M^{-1}), (B) FC(H^+) **2H⁺** (K_a = 18,990 ± 72% M^{-1}), (C) $HN(Et)_3BArF$ (K_a = 14,739 ± 40% M^{-1}). When the semi-open complex is protonated, a significant of amount of association with DMA occurs. Clearly, from the titration of the protonated $N(Et)_3$ control this association for **1H⁺** is due to association with the electron poor proton.

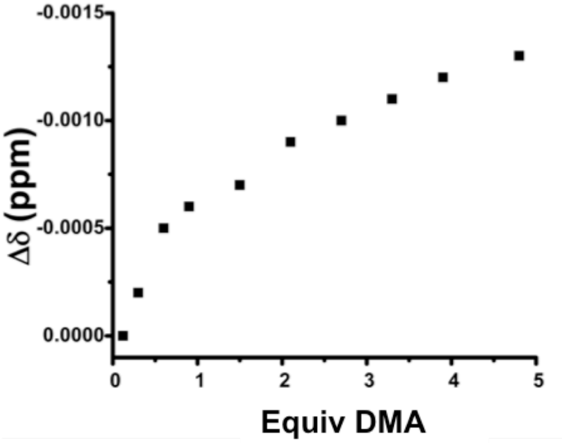

Figure S4. **Δ**δ (ppm) of dimethylacetamide (DMA) vs. equivalents of DMA added to fully closed **2** after two configurational inversions using PyHCl and NaHMDS.

Catalysis Experiments

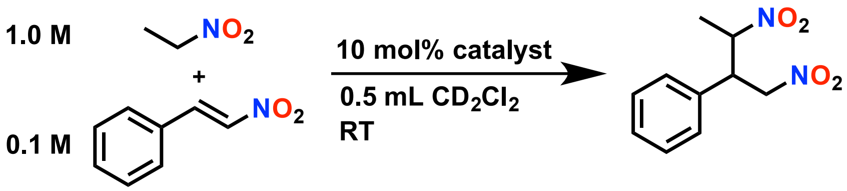

All catalysis experiments for the Michael addition of nitroethane to β–nitrostyrene were carried out at room temperature in CD_2Cl_2 under ambient conditions. Reaction progress (i.e., conversion) was tracked using ^{1}H NMR spectroscopy using the integration of the product (δ = 4.35 ppm, 1H) (See S44) versus a dichloroethane internal standard. Dichloroethane was chosen because it had no significant overlap with other resonance peaks in the spectrum and would not significantly interact with the catalyst. In all cases in which catalytic activity of a complex is studied, FC complex was used and the alternative configurational states (e.g., SO, SO(H^+)) were generated *in*

situ via addition of 1 equiv of the appropriate salt (e.g., $N(Bu)_4Cl$, PyHCl). Unless stated otherwise, catalysis experiments were carried out as follows: To a solution of β−nitrostyrene (7.4 mg, 0.05 mmol, 0.1 mL) was added 10 mol% (0.005 mmol, 0.2 mL) of the catalyst/complex/ligand. Then nitroethane (35.5 μL, 0.5 mmol) and dichloroethane (3.9 μL, 0.05 mmol) were added *via* microsyringe, and the solution was transferred to a NMR tube with an additional 0.2 mL of solvent.

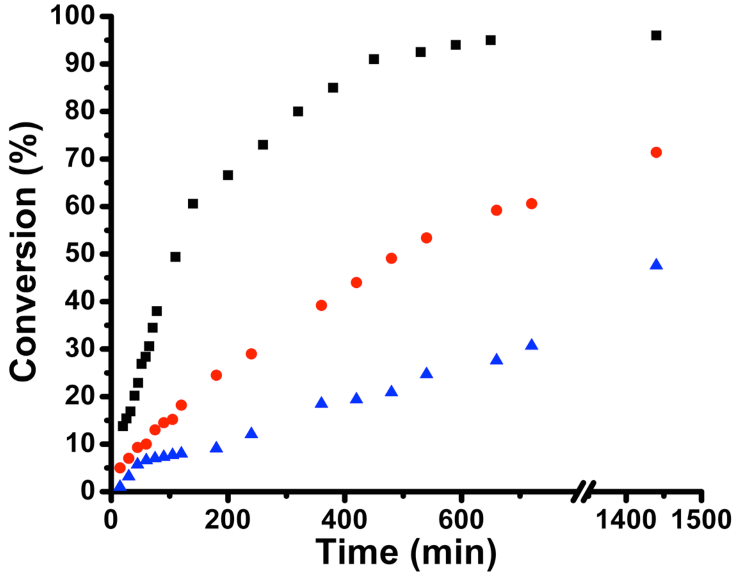

Figure S5. Different mol% loadings of FC **2**. 10 mol% (black squares), 5 mol% (red circles), and 2.5 mol% (blue triangles) (TON = 19.04 at 1450 Min).

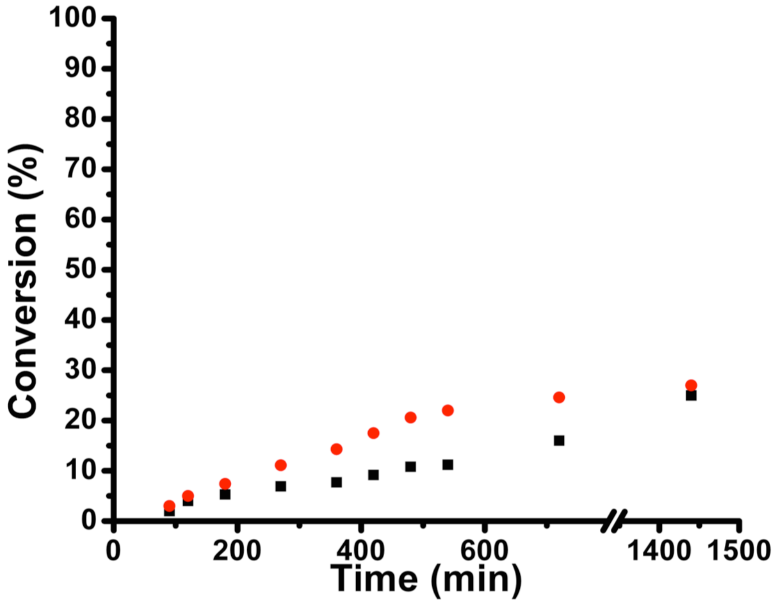

Figure S6. 10 mol% loading of SO **1** (black squares) and $N(Et)_3$ (red circles). Similarity in conversion shows that residual activity of **1** is due to monofunctional activity of the tertiary amine constituent.

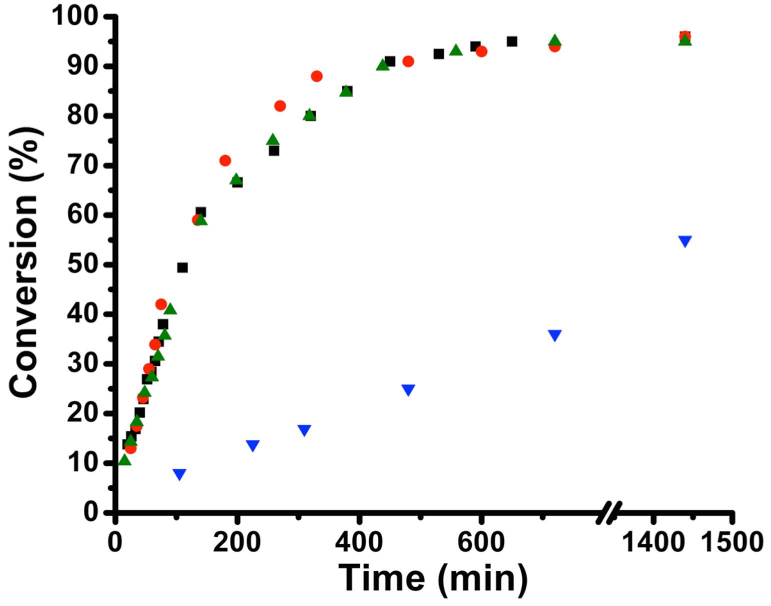

Figure S7. 10 mol% loading of FC **2** (black squares), FC **6** (*t*-butyl) (red circles), SO **5** (*t*-butyl) (green triangles), and free ligand **3** (blue triangles).

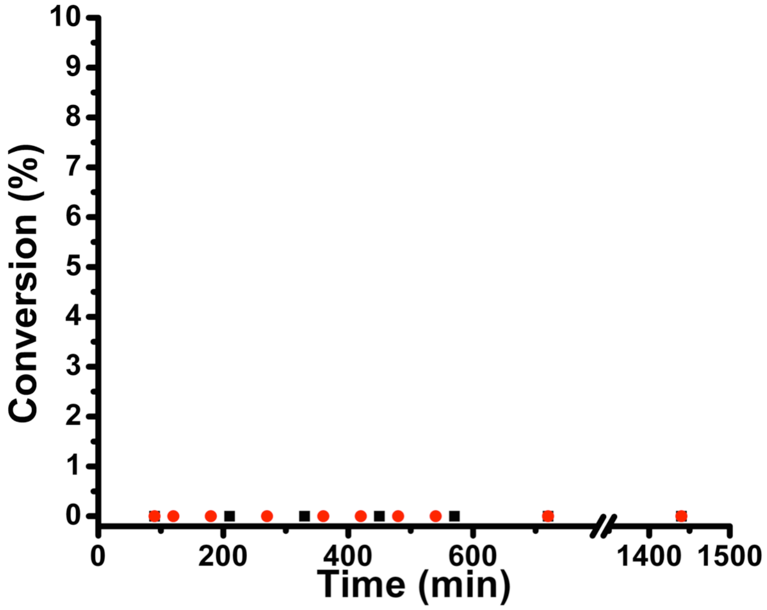

Figure S8. 10 mol% loading of SO(H$^+$) **1H$^+$** generated *in situ via* the addition of N(*n*-Bu)$_4$Cl + PyHBArF (black squares) or PyHCl (red circles) to FC **2**. Both routes give the same result.

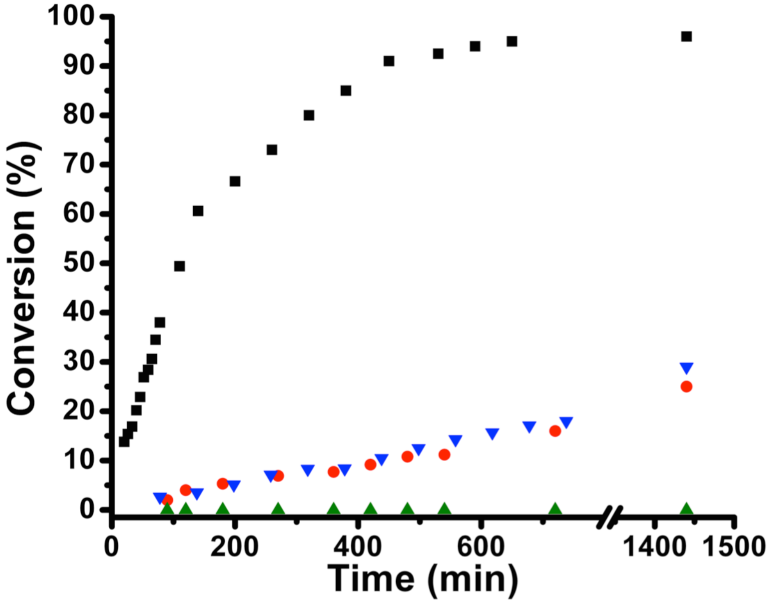

Figure S9. 10 mol% loading of FC **2** (black squares), FC(H$^+$) **2H$^+$** (blue triangles), SO **1** (red circles), and SO(H$^+$) **1H$^+$** (green triangles). Observed conversion for **2H$^+$** and **1** shows necessity of two-prong approach.

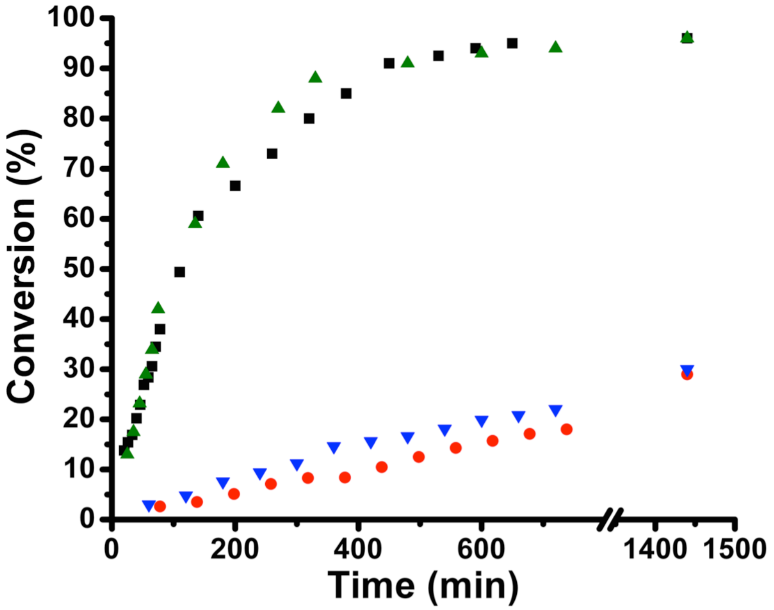

Figure S10. 10 mol% loading of FC **2** (black squares), FC **6** (*t*-butyl) (green triangles), FC(H^+) **2H⁺** (red circles), and FC(H^+) **6H⁺** (blue triangles).

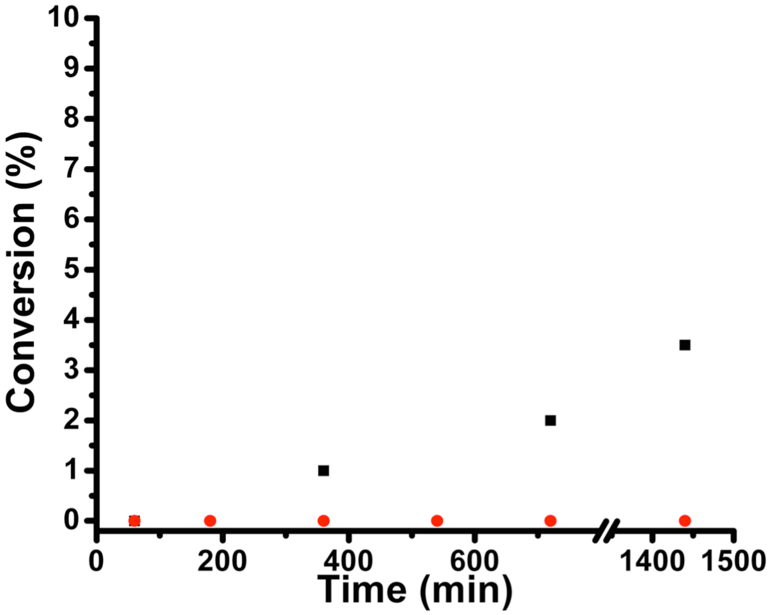

Figure S11. 10 mol% loading of ligand **4** (black rectangles) and FC model complex **13** (red circles). Residual conversion from **2** assumed to be due to weakly Lewis basic free phosphine.

***In Situ* Regulation of Catalytic Activity**

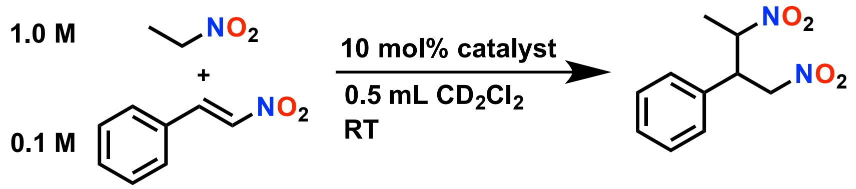

All allosteric regulation experiments for the Michael addition of nitroethane to β–nitrostyrene were carried out at room temperature in dry CD_2Cl_2. The reaction and regulatory steps were performed under inert conditions to avoid quenching of the allosteric effectors (i.e., PyHCl and NaHMDS). Reaction progress (i.e., conversion) was tracked using ^1H NMR spectroscopy using the integration of the product (δ = 4.35 ppm, 1H) (See S44) versus a dichloroethane internal standard.

To a solution of β–nitrostyrene (7.4 mg, 0.05 mmol, 0.1 mL) was added FC **2** (14.5 mg, 0.005 mmol, 0.2 mL). Then nitroethane (35.5 μL, 0.5 mmol) and dichloroethane (3.9 μL, 0.05 mmol) were added *via* microsyringe, and the solution was transferred to a J. Young NMR tube with an additional 0.2 mL of solvent. After the reaction was monitored by ^1H NMR spectroscopy for a given period of time (~1.5 h), to the reaction solution was added 1 equiv PyHCl (0.62 mg) *via* a concentrated stock solution in CD_2Cl_2 to generate **1H⁺**. The conversion was again monitored for a given period of time (~1–1.5 h). To the reaction was then added 1 equiv of NaHMDS (0.98 mg) *via* a concentrated stock solution in THF-d_8 to generate **2**. For polyinversion experiments, this process was repeated in the same manner.

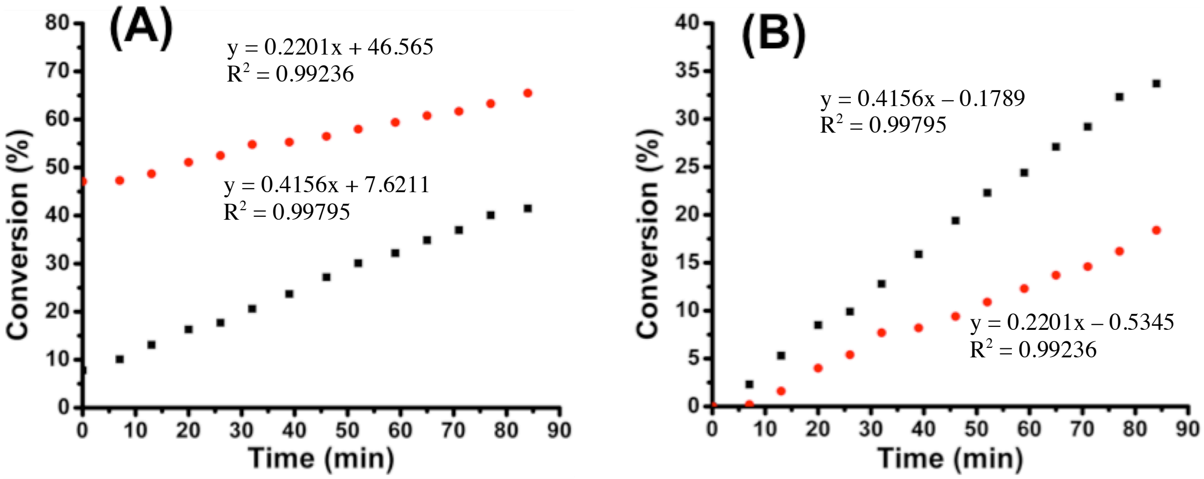

Figure S12. Regions of catalytic conversion with FC **2** from the allosteric regulation experiment found in Figure 5A (A) normalized for time, (B) normalized for time and conversion. First region (black squares) and second region (red circles).

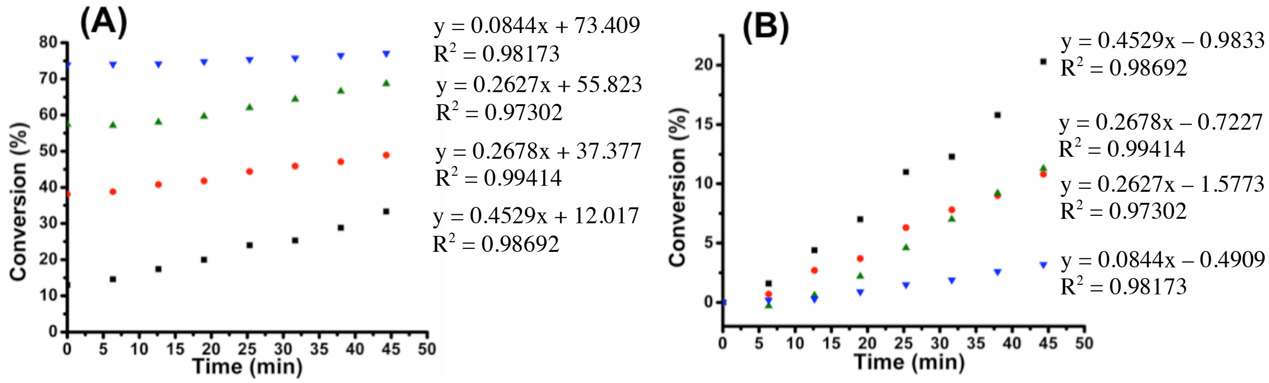

Figure S13. Regions of catalytic conversion with FC **2** from the allosteric regulation experiment found in Figure 5B (A) normalized for time, (B) normalized for time and conversion. First region (black squares), second region (red circles), third region (green triangles), and fourth region (blue triangles)

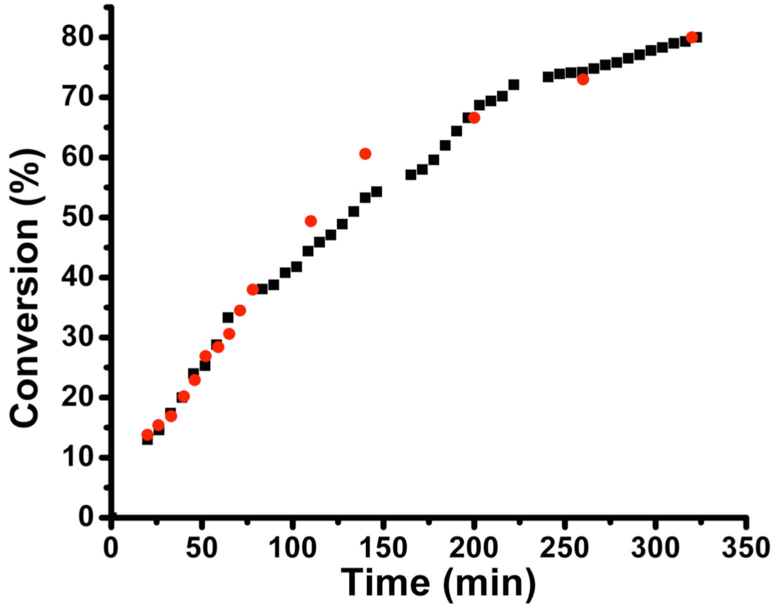

Figure S14. Regions of catalytic conversion with FC **2** from the allosteric regulation experiment found in Figure 5B plotted (black squares) with the catalytically dormant regions of SO(H$^+$) **1H$^+$** removed. Non-allosterically regulated catalytic conversion with FC **2** (red circles).

In Situ Regulation of Catalytic Activity with Added Substrate

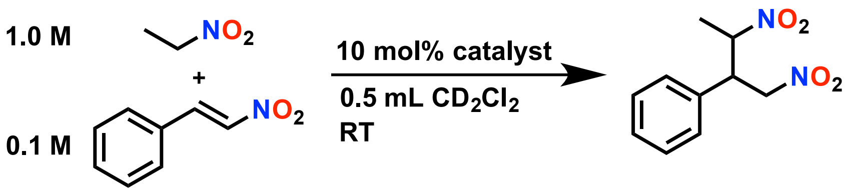

All allosteric regulation experiments for the Michael addition of nitroethane to β−nitrostyrene were carried out at room temperature in dry CD_2Cl_2. The reaction and regulatory steps were performed under inert conditions to avoid quenching of the allosteric effectors (i.e., PyHCl and NaHMDS). Reaction progress (i.e., conversion) was tracked using ^{1}H NMR

spectroscopy using the integration of the product (δ = 4.35 ppm, 1H) (See S44) versus a dichloroethane internal standard.

To a solution of β–nitrostyrene (7.4 mg, 0.05 mmol, 0.1 mL) was added FC **2** (14.5 mg, 0.005 mmol, 0.2 mL). Then nitroethane (35.5 μL, 0.5 mmol) and dichloroethane (3.9 μL, 0.05 mmol) were added *via* microsyringe, and the solution was transferred to a J. Young NMR tube with an additional 0.2 mL of solvent. After the reaction was monitored by ^{1}H NMR spectroscopy for a given period of time (~1.5 h), to the reaction solution was added 1equiv PyHCl (0.62 mg) *via* a concentrated stock solution in CD_2Cl_2 to generate **1H⁺**. The conversion was again monitored for a given period of time (~1–1.5 h). At this point the moles of β–nitrostyrene consumed by the reaction to this point was calculated and replenished in the reaction solution *via* a concentrated CD_2Cl_2 stock solution. To the reaction was then added 1 equiv of NaHMDS (0.98 mg) *via* a concentrated stock solution in THF-d_8 to generate **2**. This process was repeated in the same manner for each inversion.

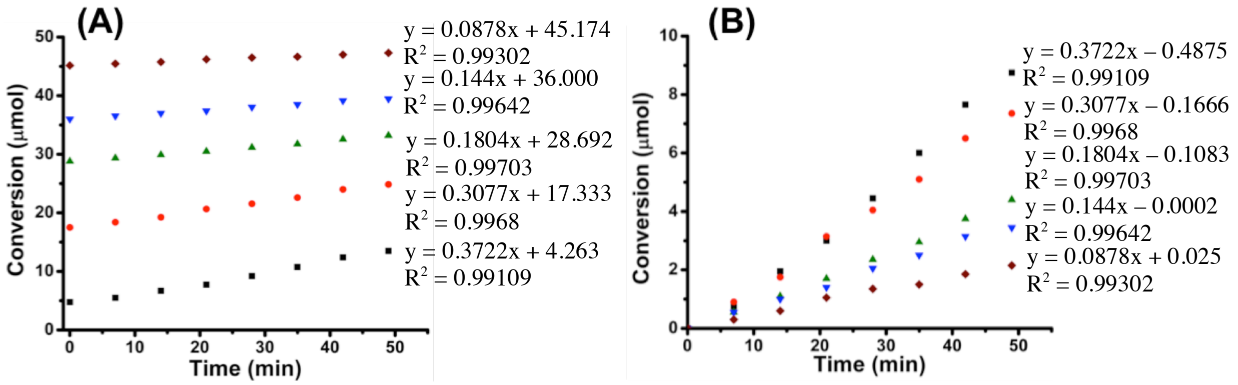

Figure S15. Regions of catalytic conversion with FC **2** from the allosteric regulation experiment found in Figure 5C (A) normalized for time, (B) normalized for time and conversion. First region (black squares), second region (red circles), third region (green triangles), fourth region (blue triangles), and fifth region (maroon diamonds).

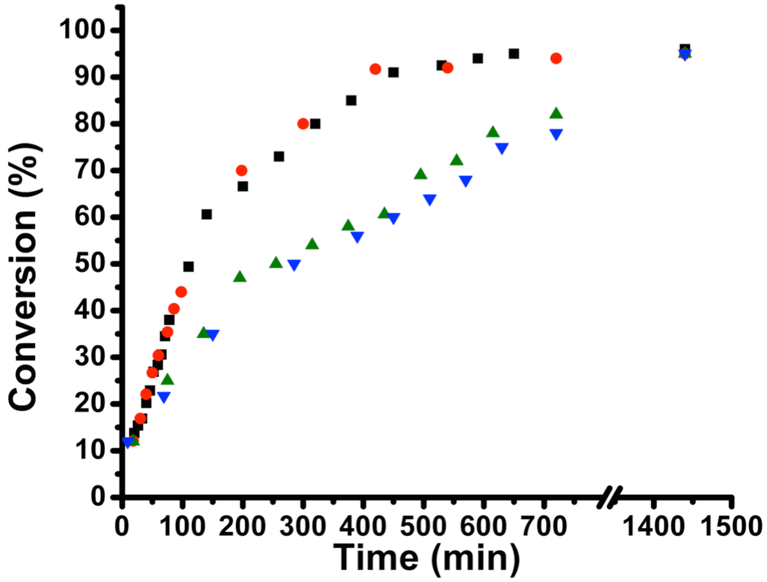

Figure S16. 10 mol% loading of FC **2** (black squares), FC **2** after one configurational inversion (i.e., FC–SO(H$^+$)–FC) before β–nitrostyrene addition (red circles), FC **2** after three configurational inversions before β–nitrostyrene addition (blue triangles), and FC **2** with three equivalents of neutralized PyHCl and NaHMDS added (green triangles).

Catalysis Experiments

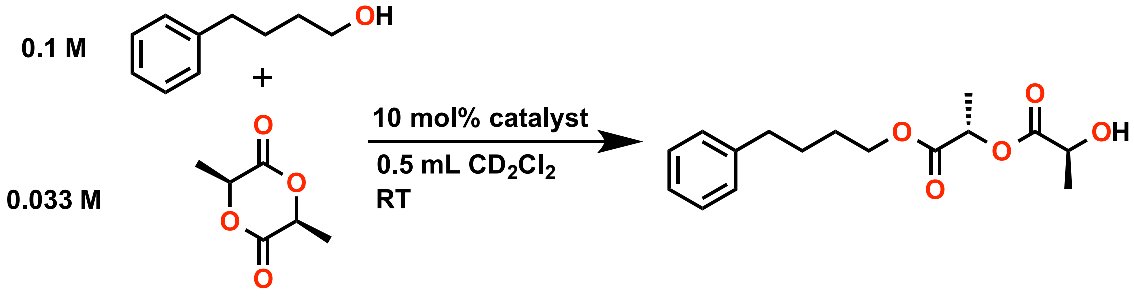

All catalysis experiments for the stoichiometric ring opening of L–(–)–lactide were carried out at room temperature in CD_2Cl_2 under inert conditions. FC **2** was dried over night in a THF solution with CaH_2. L–(–)–lactide was recrystallized 3x from toluene. 4–phenyl–1–butanol was dried over night in a THF solution with CaH_2. Solvent was dried over 3 Å sieves. Reaction

progress (i.e., conversion) was tracked using ^{1}H NMR spectroscopy using the integration of the product (δ = 5.20 ppm, 1H) (See S45) versus a dichloroethane internal standard. In all cases in which catalytic activity of a complex is studied, FC complex was used and the alternative configurational states (e.g., SO, SO(H^+)) were generated *in situ via* addition of 1 equiv of the appropriate salt (e.g., $N(Bu)_4Cl$, PyHCl). Unless stated otherwise, catalysis experiments were carried out as follows: To a solution of L–(–)–lactide (2.3 mg, 0.0166 mmol, 0.1 mL) was added 10 mol% (0.00166 mmol, 0.2 mL) of the complex. Then 4–phenyl–1–butanol (7.6 µL, 0.5 mmol) and dichloroethane (1.3 µL, 0.0166 mmol) were added *via* microsyringe, and the solution was transferred to a J. Young NMR tube with an additional 0.2 mL of solvent.

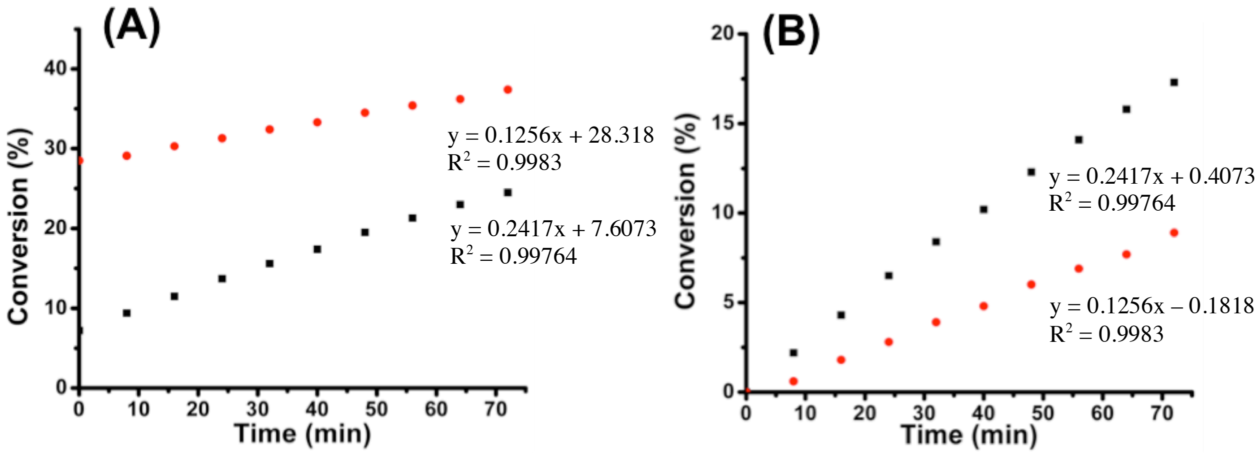

Figure S17. Regions of catalytic conversion with FC **2** from the allosteric regulation experiment found in Figure 6C (A) normalized for time, (B) normalized for time and conversion. First region (black squares) and second region (red circles).

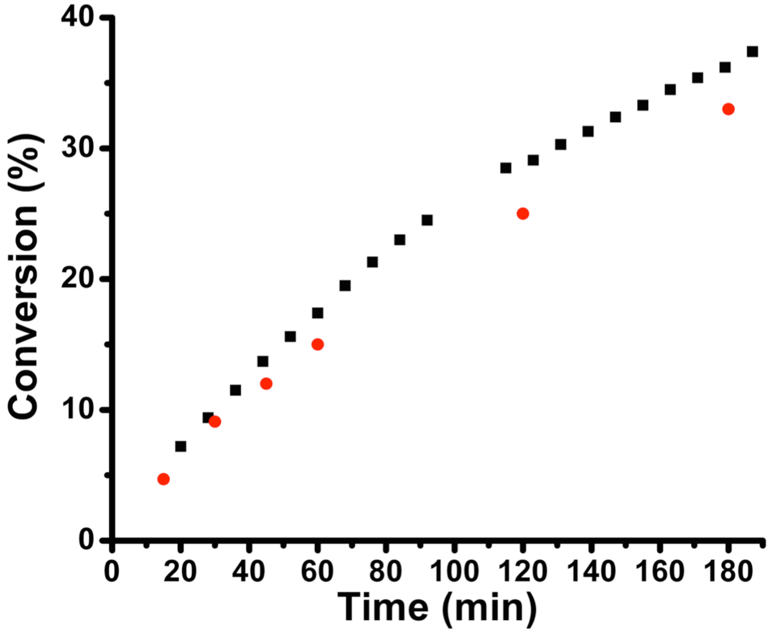

Figure S18. Regions of catalytic conversion with FC **2** from the allosteric regulation experiment found in Figure 6C plotted (black squares) with the catalytically dormant regions of SO(H^+) **$1H^+$** removed. Non-allosterically regulated catalytic conversion with FC **2** (red circles).

Single Crystal X−Ray Diffraction Studies

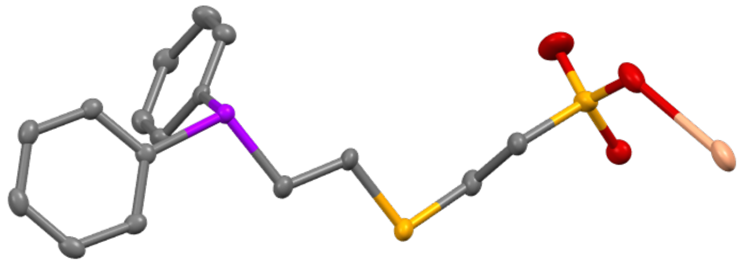

Figure S19. Single crystal solid-state structure of ligand **4**. Hydrogens and solvent omitted for clarity. Thermal ellipsoids drawn to 50% probability: C, gray; P, purple; S, orange; O, red; Na, pale pink.

A suitable crystal was selected and the crystal was mounted on a MITIGEN holder in Paratone oil on a Kappa Apex 2 diffractometer. The crystal was kept at 100.02 K during data collection. Using Olex2, the structure was solved with the ShelXT structure solution program using Direct Methods and refined with the ShelXL refinement package using Least Squares minimisation.

Distance restraints were imposed on the disordered oxygen atoms and refined hydrogen atoms. Rigid bond restraints were imposed on the displacement parameters as well as restraints on similar amplitudes separated by less than 1.7 Ang. on the disordered oxygen atoms.

The solvent masking procedure as implemented in Olex2 was used to remove the electronic contribution of solvent molecules from the refinement. As the exact solvent content is not known, only the atoms used in the refinement model are reported in the formula here. Total solvent accessible volume / cell = 340.4 Å^3 [8.5%] Total electron count / cell = 78.5.

Table S1. Crystallographic Information for **4**.

Empirical formula	$C_{16}H_{21}NaO_{4.5}PS_2$
Formula weight	403.41
Temperature (K)	100.02
Crystal system	monoclinic
Space group	$P2_1/c$
a (Å), b (Å), c (Å)	29.3940(10), 5.6190(2), 25.1528(10)
A (°), β (°), γ (°)	90, 104.918(2), 90
Volume (Å^3)	4014.3(3)

Z	8
Calculated density (mg mm^{-3})	1.335
Absorption Coefficient (mm^{-1})	3.543
F(000)	1688
Crystal size (mm^{3})	0.345 × 0.109 × 0.025
2Θ range for data collection	3.11 to 130.414°
Index ranges	$-34 \leq h \leq 32$, $-6 \leq k \leq 6$, $-29 \leq l \leq 25$
Reflections collected	27769
Independent reflections	6828[R(int) = 0.0405]
Data/restraints/parameters	6828/59/488
Goodness-of-fit on F^2	1.080
Final R indexes [I>2σ (I)]	R_1 = 0.0532, wR_2 = 0.1414
Final R indexes [all data]	R_1 = 0.0581, wR_2 = 0.1449
Largest diff. peak/hole (e Å^{-3})	1.746/-0.758

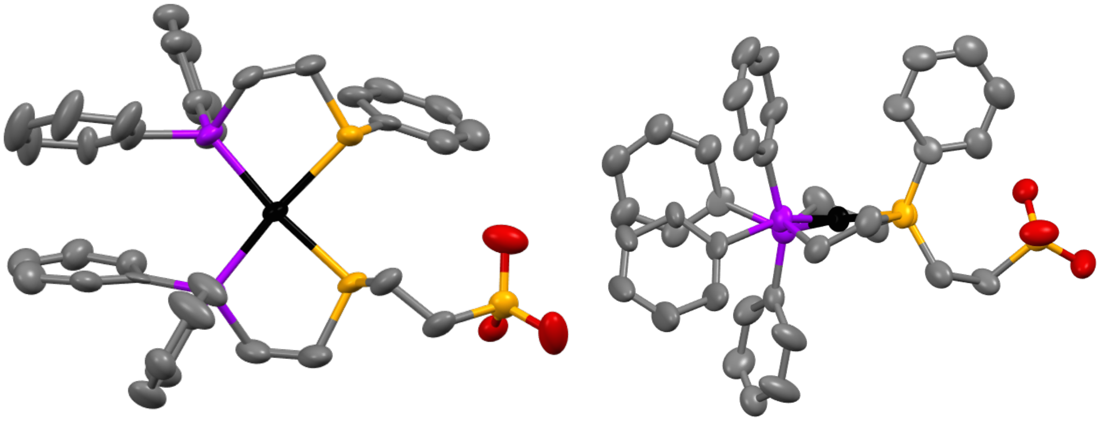

Figure S20. Single crystal solid-state structure of FC **13** face-on (left) and top-down (right). Outer-sphere counteranions, hydrogens and solvent omitted for clarity. Thermal ellipsoids drawn to 50% probability: C, gray; P, purple; S, orange; O, red; Na, pale pink; Pt, black.

A suitable crystal was selected and the crystal was mounted on a MITIGEN holder in Paratone oil on a Kappa Apex 2 diffractometer. The crystal was kept at 99.99 K during data collection. Using Olex2, the structure was solved with the ShelXT structure solution program using Direct Methods and refined with the ShelXL refinement package using Least Squares minimization.

Distance restraints were imposed on the disordered rings. The enhanced rigid-bond restraint (SHELX keyword RIGU) was applied globally. (Acta Cryst. A68 (2012) 448-451).

Table S2. Crystallographic Information for **13**.

Empirical formula	$C_{36}H_{37}BF_4O_3P_2PtS_3$
Formula weight	957.67
Temperature (K)	99.99
Crystal system	monoclinic
Space group	$P2_1/n$
a (Å), b (Å), c (Å)	18.1695(7), 9.2664(4), 22.3000(9)
A (°), β (°), γ (°)	90, 101.881(3), 90
Volume (Å^3)	3674.1(3)
Z	4
Calculated density (mg mm^{-3})	1.731
Absorption Coefficient (mm^{-1})	10.046

F(000)	1896
Crystal size (mm^3)	0.075 × 0.03 × 0.006
2Θ range for data collection	7.03 to 132.878°
Index ranges	$-21 \le h \le 21$, $-10 \le k \le 6$, $-26 \le l \le 26$
Reflections collected	24761
Independent reflections	6406[R(int) = 0.0968]
Data/restraints/parameters	6406/653/561
Goodness-of-fit on F^2	1.017
Final R indexes [I>2σ (I)]	$R_1 = 0.0445$, $wR_2 = 0.0989$
Final R indexes [all data]	$R_1 = 0.0708$, $wR_2 = 0.1116$
Largest diff. peak/hole (e $Å^{-3}$)	1.081/-0.954

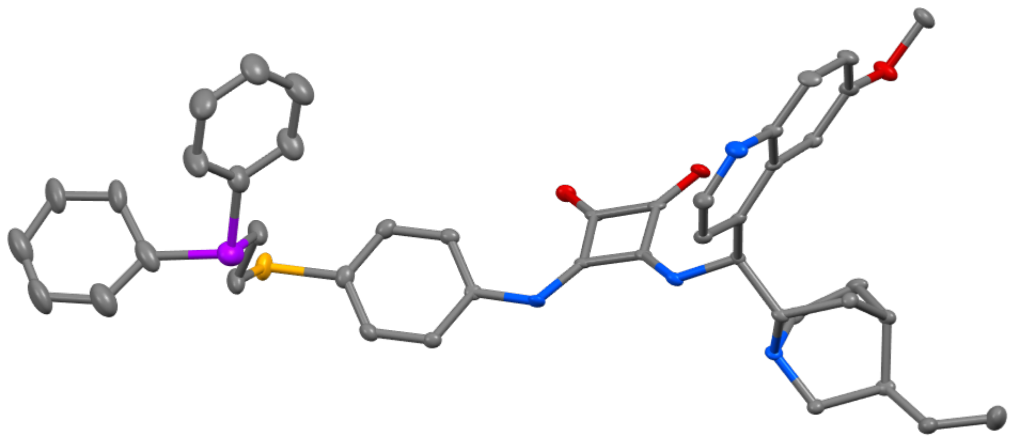

Figure S21. Single crystal solid-state structure of **S2**. Hydrogens and solvent omitted for clarity. Thermal ellipsoids drawn to 50% probability: C, gray; P, purple; S, orange; O, red; N, blue.

A suitable crystal was selected and mounted in inert oil and transferred to the cold gas stream of a Kappa Apex 2 diffractometer. The crystal was kept at 100.0 K during data collection. Using Olex2, the structure was solved with the ShelXT structure solution program using Direct Methods and refined with the ShelXL refinement package using Least Squares minimization.

Rigid bond restraints were imposed on the displacement parameters as well as restraints on similar amplitudes separated by less than 1.7 Ang. on the disordered phenyl rings.

Table S3. Crystallographic Information for **S2**.

Empirical formula	$C_{89}H_{98}Cl_2N_8O_8P_2S_2$
Formula weight	1604.71
Temperature (K)	100.0
Crystal system	monoclinic
Space group	C2
a (Å), b (Å), c (Å)	48.8875(19), 6.1721(2), 13.7803(5)
A (°), β (°), γ (°)	90, 103.831(3), 90
Volume (Å^3)	4037.5(3)
Z	2
Calculated density (mg mm^{-3})	1.320

Absorption Coefficient (mm^{-1})	2.084
F(000)	1696
Crystal size (mm^3)	0.11 × 0.092 × 0.011
2Θ range for data collection	6.606 to 130.274°
Index ranges	$-56 \leq h \leq 56$, $-7 \leq k \leq 7$, $-15 \leq l \leq 16$
Reflections collected	15230
Independent reflections	6673[R(int) = 0.0346]
Data/restraints/parameters	6673/251/598
Goodness-of-fit on F^2	1.042
Final R indexes [I>2σ (I)]	R_1 = 0.0488, wR_2 = 0.1288
Final R indexes [all data]	R_1 = 0.0517, wR_2 = 0.1311
Largest diff. peak/hole (e $Å^{-3}$)	0.405/-0.799

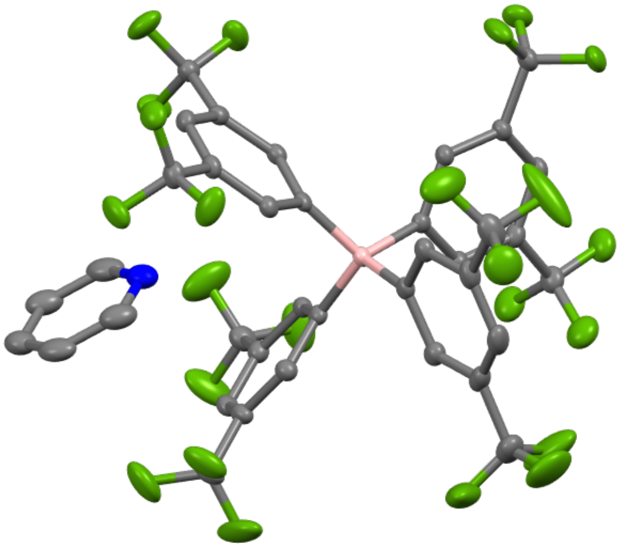

Figure S22. Single crystal solid-state structure of PyHBArF. Hydrogens and solvent omitted for clarity. Thermal ellipsoids drawn to 50% probability: C, gray; N, blue; F, green; B, pale pink.

A suitable crystal was selected and the crystal was mounted on a MITIGEN holder in Paratone oil on a Kappa Apex 2 diffractometer. The crystal was kept at 100.01 K during data collection. Using Olex2, the structure was solved with the ShelXT structure solution program using Direct Methods and refined with the ShelXL refinement package using Least Squares minimization.

Distance restraints were imposed on the disordered flourine atoms. The enhanced rigid-bond restraint (SHELX keyword RIGU) was applied on the disordered flourine atoms. (Acta Cryst. A68 (2012) 448-451). The disordered pyridinium was constrained to certain positions.

Table S4. Crystallographic Information for PyHBArF

Empirical formula	$C_{37}H_{18}BNF_{24}$
Formula weight	943.33
Temperature (K)	100.01
Crystal system	triclinic
Space group	P-1
a (Å), b (Å), c (Å)	12.3832(6), 12.7027(6), 13.6428(6)
Α (°), β (°), γ (°)	79.884(3), 76.218(3), 63.750(3)
Volume (Å^3)	1863.20(16)
Z	2

Calculated density (mg mm^{-3})	1.681
Absorption Coefficient (mm^{-1})	0.182
F(000)	936
Crystal size (mm^3)	0.199 × 0.095 × 0.017
2Θ range for data collection	3.586 to 58.26°
Index ranges	$-16 \leq h \leq 16, -17 \leq k \leq 17, -18 \leq l \leq 18$
Reflections collected	27044
Independent reflections	9629[R(int) = 0.0264]
Data/restraints/parameters	9629/53/600
Goodness-of-fit on F^2	1.060
Final R indexes [I>2σ (I)]	$R_1 = 0.0636$, $wR_2 = 0.1666$
Final R indexes [all data]	$R_1 = 0.0877$, $wR_2 = 0.1830$
Largest diff. peak/hole (e $Å^{-3}$)	1.108/-0.673

Supporting Information References

1. A. M. Brown, M. V. Ovchinnikov and C. A. Mirkin, *Angew. Chem., Int. Ed.*, 2005, **44**, 4207-4209.
2. A. M. Spokoyny, C. W. Machan, D. J. Clingerman, M. S. Rosen, M. J. Wiester, R. D. Kennedy, C. L. Stern, A. A. Sarjeant and C. A. Mirkin, *Nat. Chem.*, 2011, **3**, 590-596.
3. H. J. Yoon, J. Kuwabara, J.-H. Kim and C. A. Mirkin, *Science* 2010, **330**, 66-69.
4. C. M. McGuirk, J. Mendez-Arroyo, A. M. Lifschitz and C. A. Mirkin, *J. Am. Chem. Soc.*, 2014, **136**, 16594-16601.
5. P. Casuso, I. Loinaz, M. Möller, P. Carrasco, J. A. Pomposo, H. J. Grande and I. Odriozola, *Supramol. Chem.*, 2009, **21**, 581-584.
6. B. Vakulya, S. Varga, A. Csámpai and T. Soós, *Org. Lett.*, 2005, **7**, 1967-1969.
7. Y. Zhu, J. P. Malerich and V. H. Rawal, *Angew. Chem. Int. Ed.*, 2010, **49**, 153-156.
8. E. Paetzold, M. Michalik and G. Oehme, *J. Prakt. Chem. /Chem-Ztg*, 1997, **339**, 38-43.
9. G. te Velde, F. M. Bickelhaupt, E. J. Baerends, C. Fonseca Guerra, S. J. A. van Gisbergen, J. G. Snijders and T. Ziegler, *J. Comput. Chem.*, 2001, **22**, 931-967.
10. M. Ernzerhof and G. E. Scuseria, *J. Chem. Phys.*, 1999, **110**, 5029-5036.

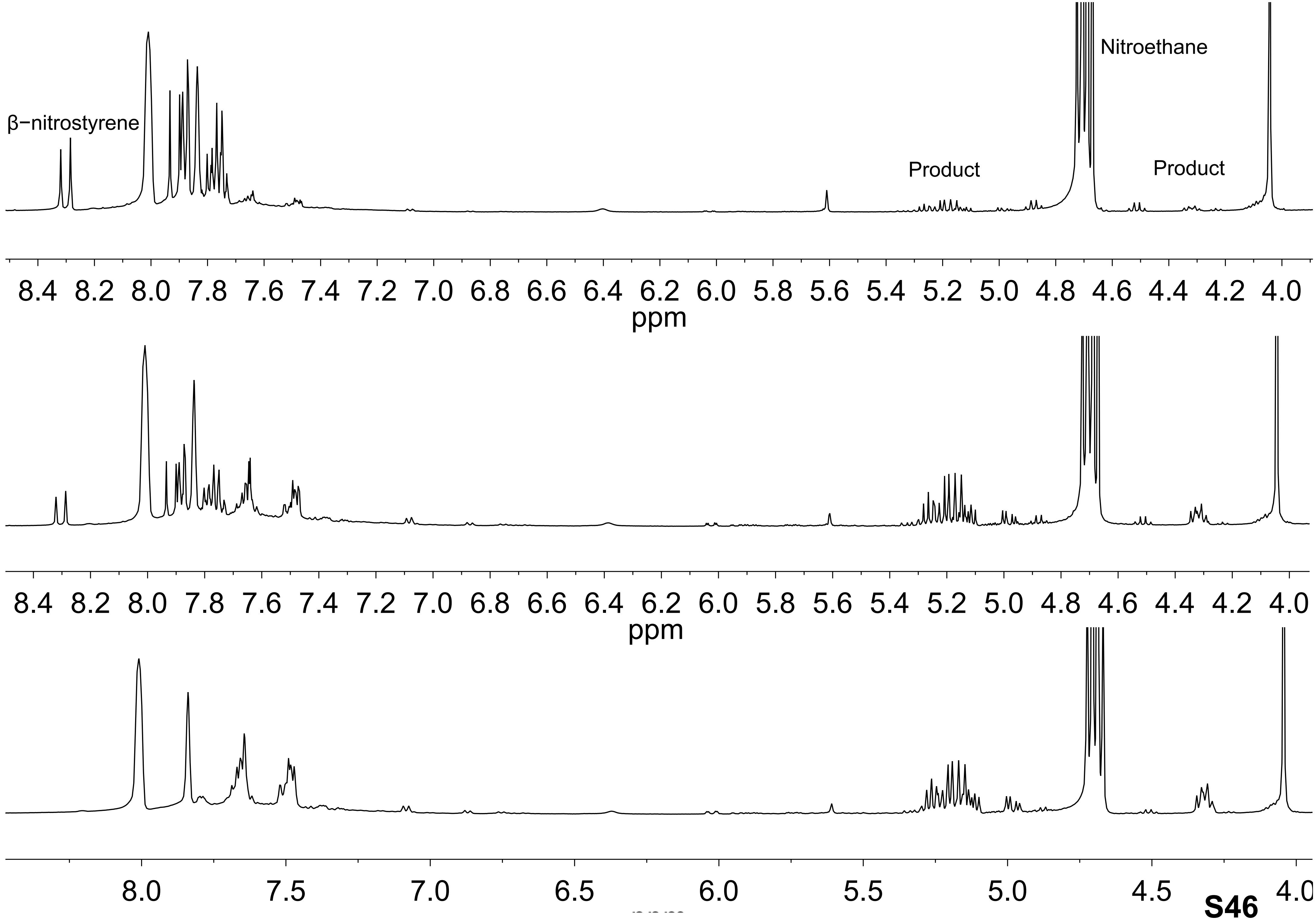
β−nitrostyrene
Product
Nitroethane
Product
8.4 8.2 8.0 7.8 7.6 7.4 7.2 7.0 6.8 6.6 6.4 6.2 6.0 5.8 5.6 5.4 5.2 5.0 4.8 4.6 4.4 4.2 4.0
ppm
8.4 8.2 8.0 7.8 7.6 7.4 7.2 7.0 6.8 6.6 6.4 6.2 6.0 5.8 5.6 5.4 5.2 5.0 4.8 4.6 4.4 4.2 4.0
ppm
8.0 7.5 7.0 6.5 6.0 5.5 5.0 4.5 4.0

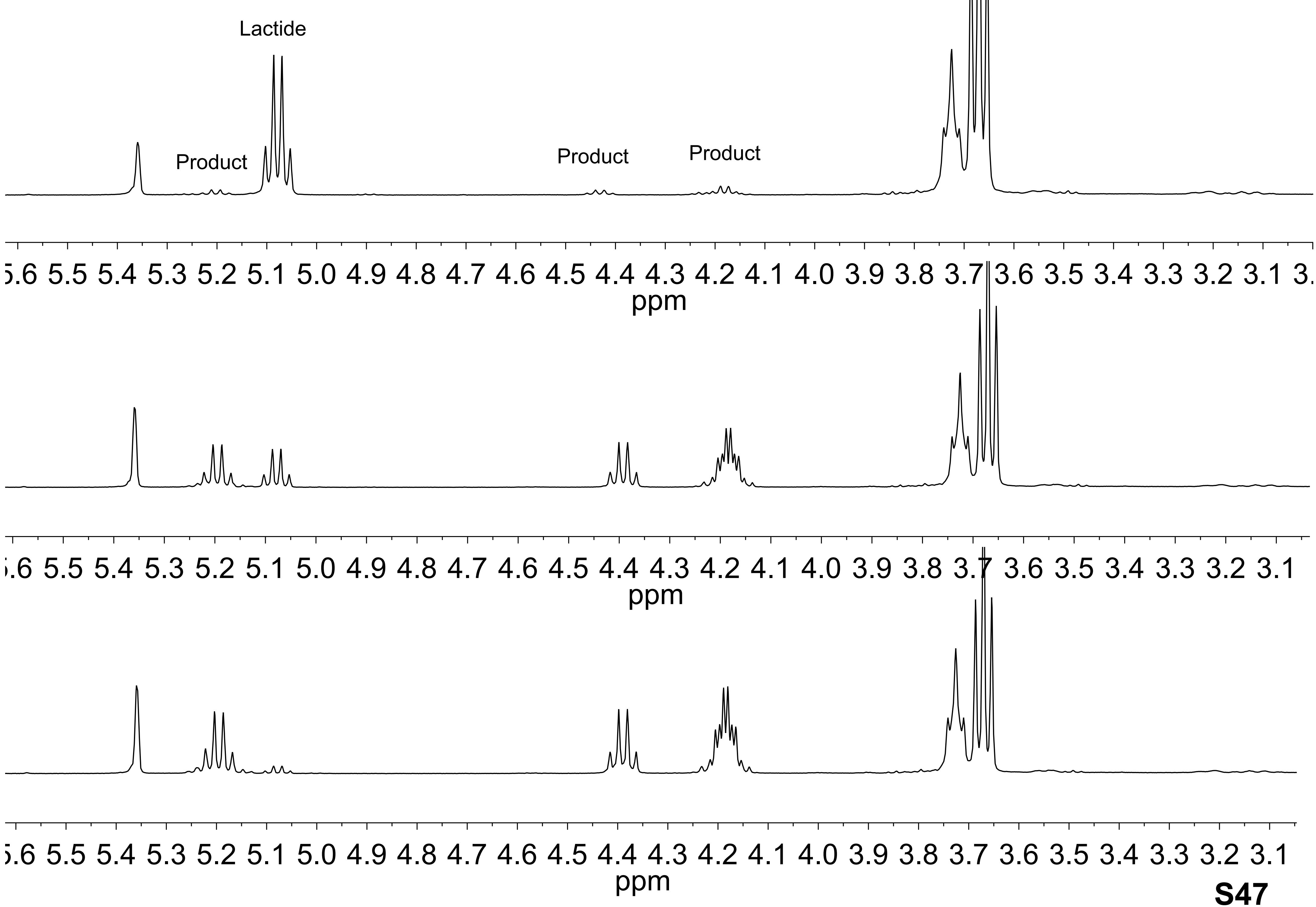
Lactide
Product
Product
Product
ppm
ppm
ppm

$^{31}P\{^{1}H\}$ NMR Spectrum of

3−(4−(2−diphenylphosphanylethylthio)phenylamino−4−methoxycyclobut−3−ene−1,2−dione in DMSO-d_6

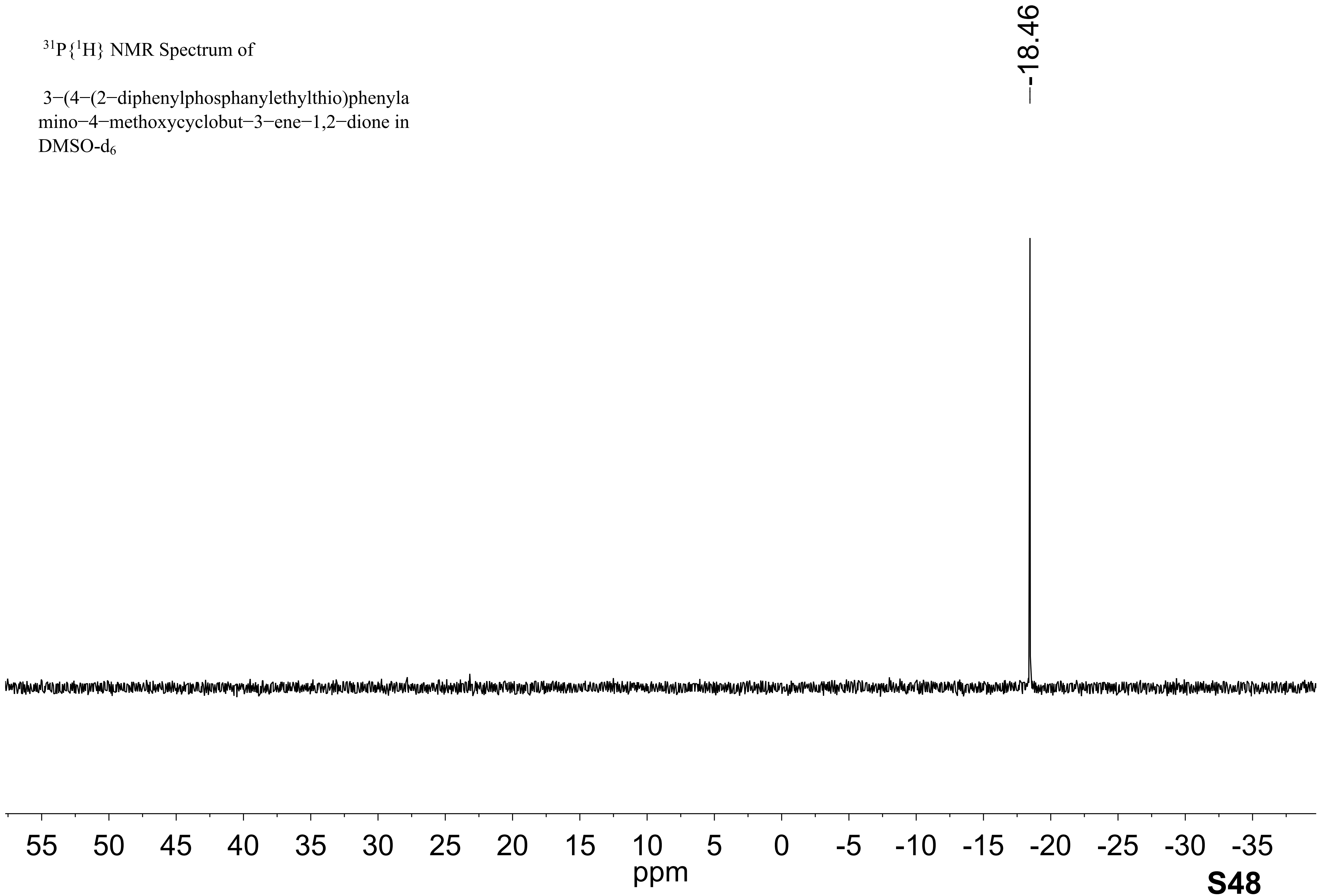

^{1}H NMR Spectrum of

3−(4−(2−diphenylphosphanylethylthio)pheny
lamino−4−methoxycyclobut−3−ene−1,2−dio
ne in DMSO-d_6

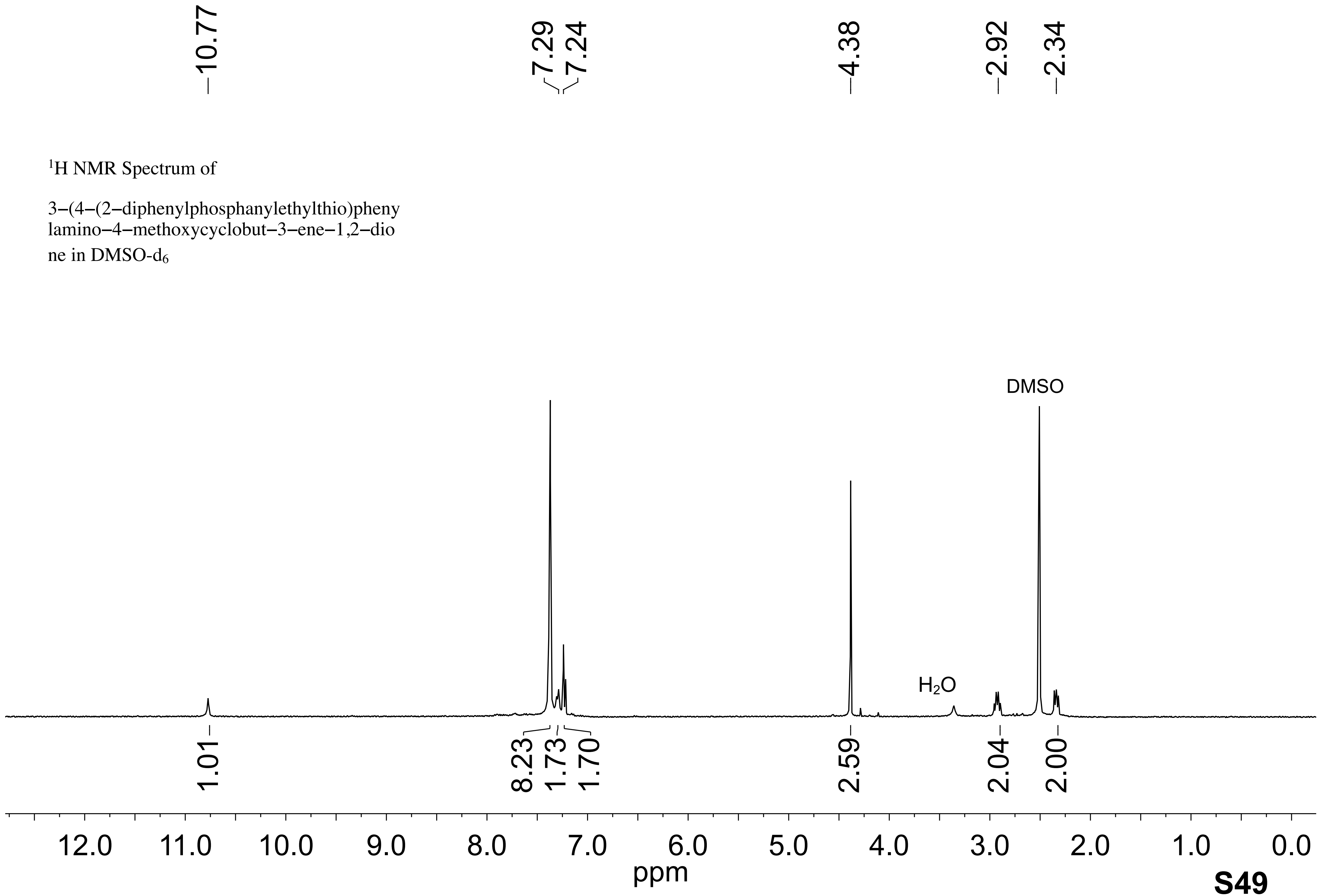

$^{31}P\{^1H\}$ NMR Spectrum of

Ligand **3** in CD_2Cl_2

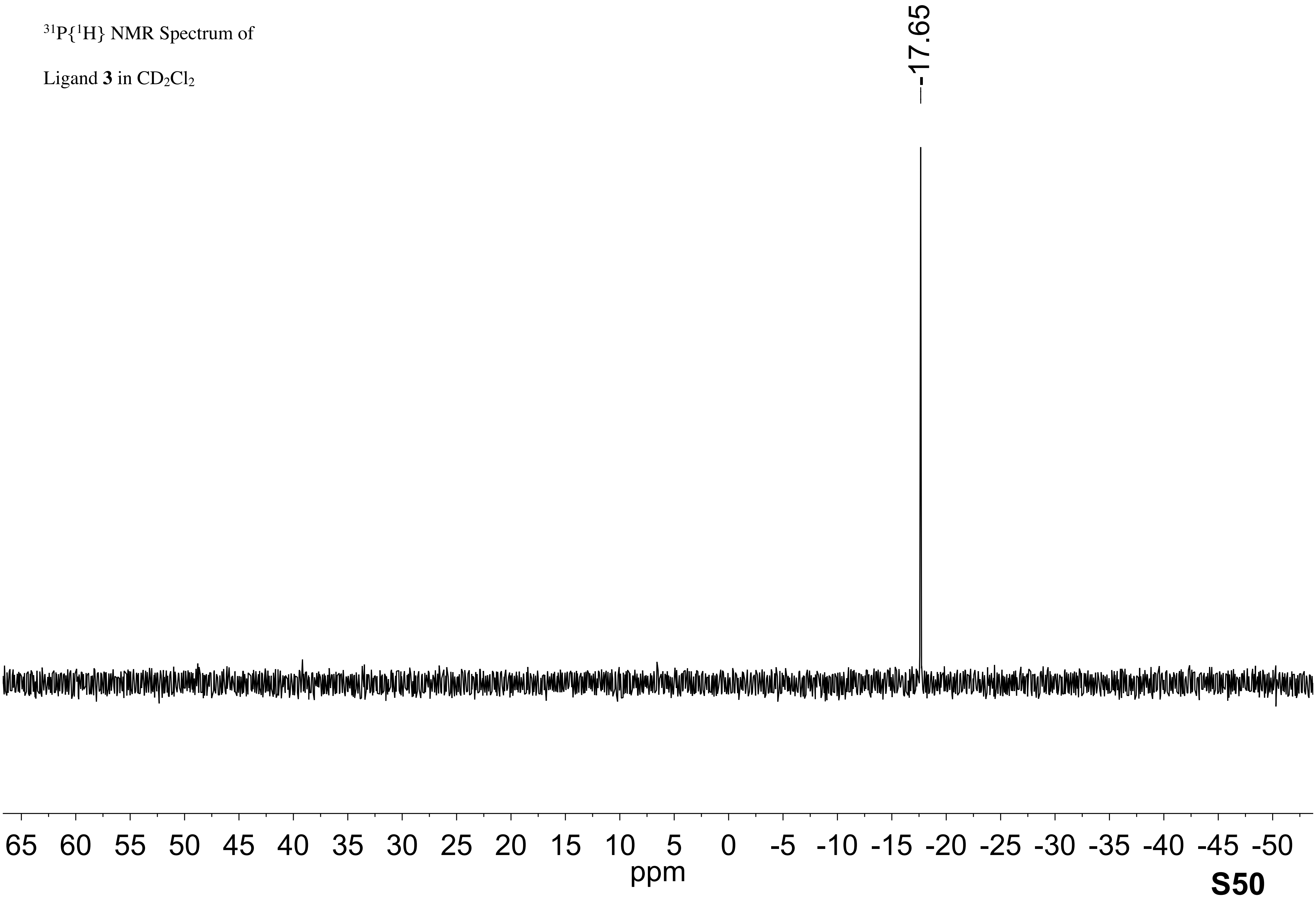

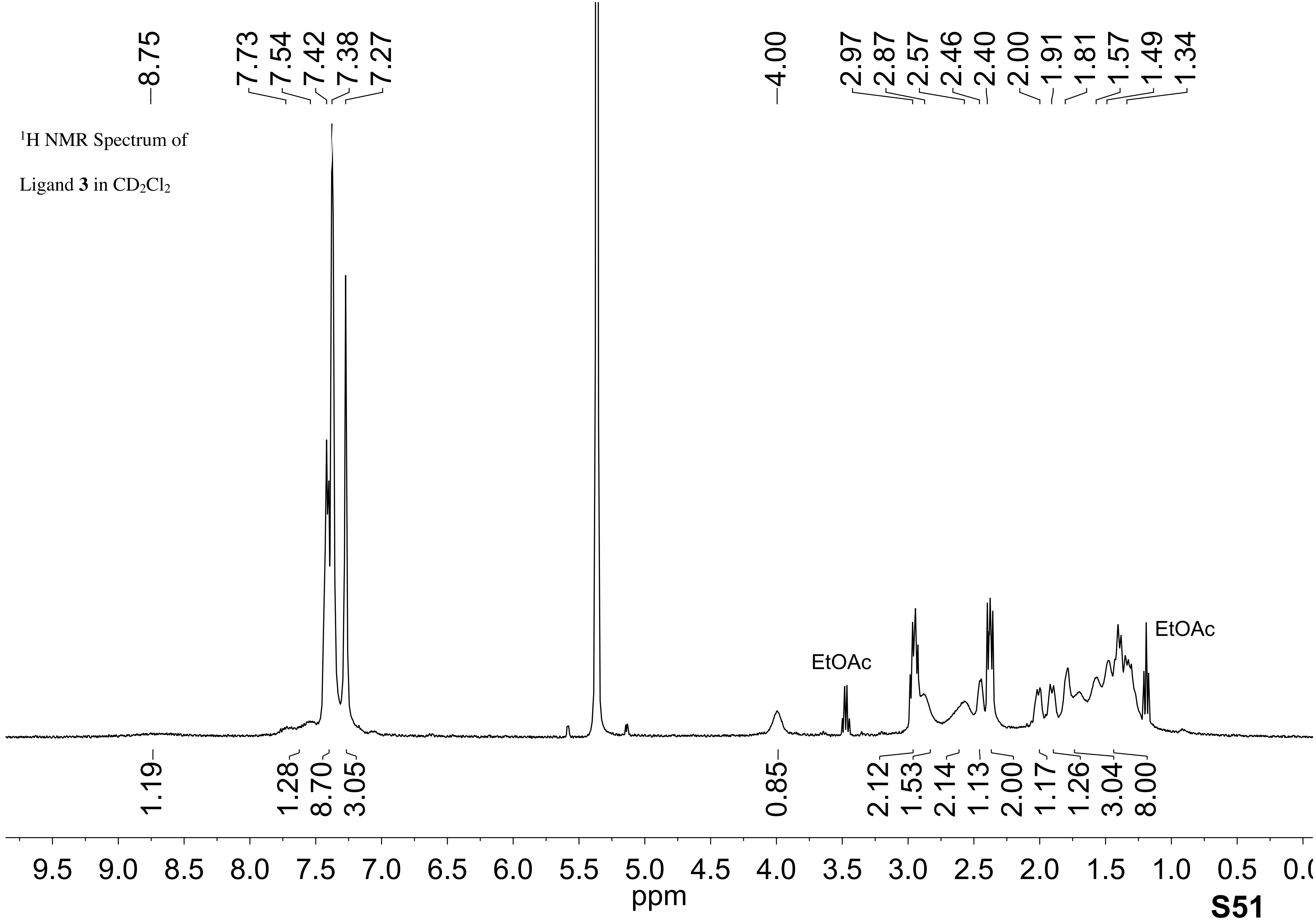

¹H NMR Spectrum of
Ligand 3 in CD2Cl2
8.75
7.73
7.54
7.42
7.38
7.27
4.00
2.97
2.87
2.57
2.46
2.40
2.00
1.91
1.81
1.57
1.49
1.34
EtOAc
EtOAc
1.19
1.28
8.70
3.05
0.85
2.12
1.53
2.14
1.13
2.00
1.17
1.26
3.04
8.00
9.5
9.0
8.5
8.0
7.5
7.0
6.5
6.0
5.5
5.0
4.5
4.0
3.5
3.0
2.5
2.0
1.5
1.0
0.5
ppm

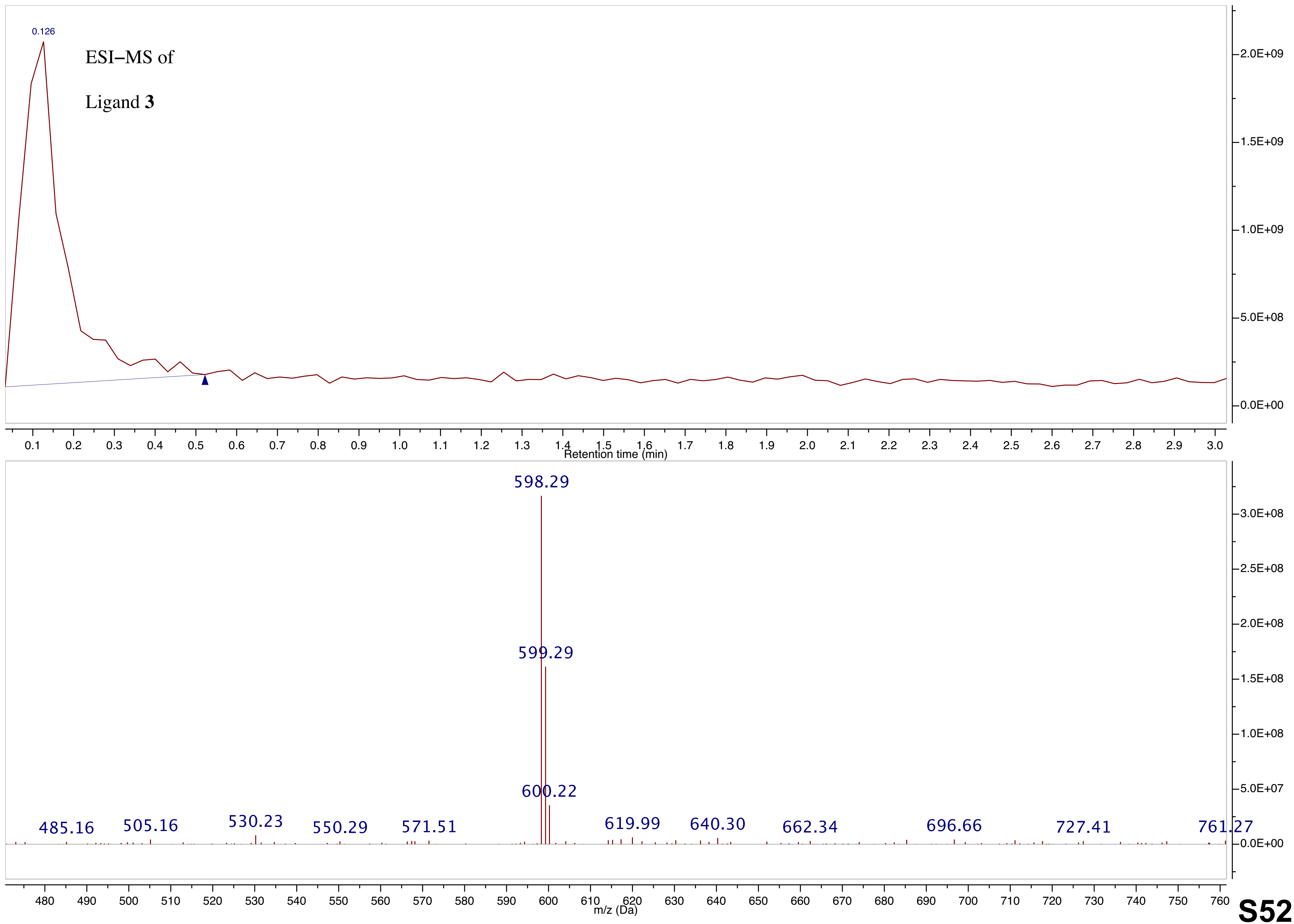

0.126
ESI–MS of
Ligand **3**
Retention time (min)
598.29
599.29
600.22
485.16
505.16
530.23
550.29
571.51
619.99
640.30
662.34
696.66
727.41
761.27
m/z (Da)

$^{31}P\{^1H\}$ NMR Spectrum of

Ligand **4** in D_2O

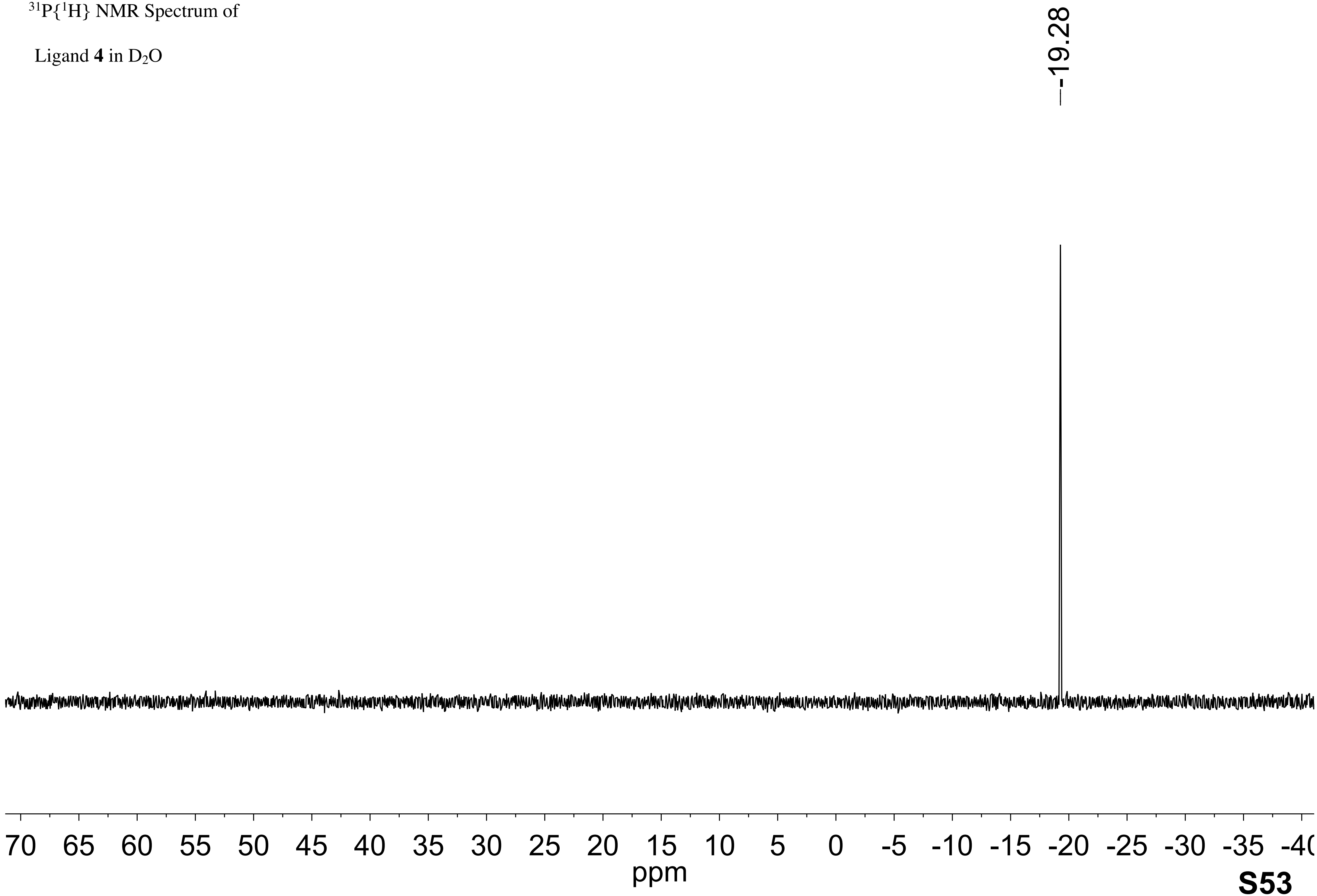

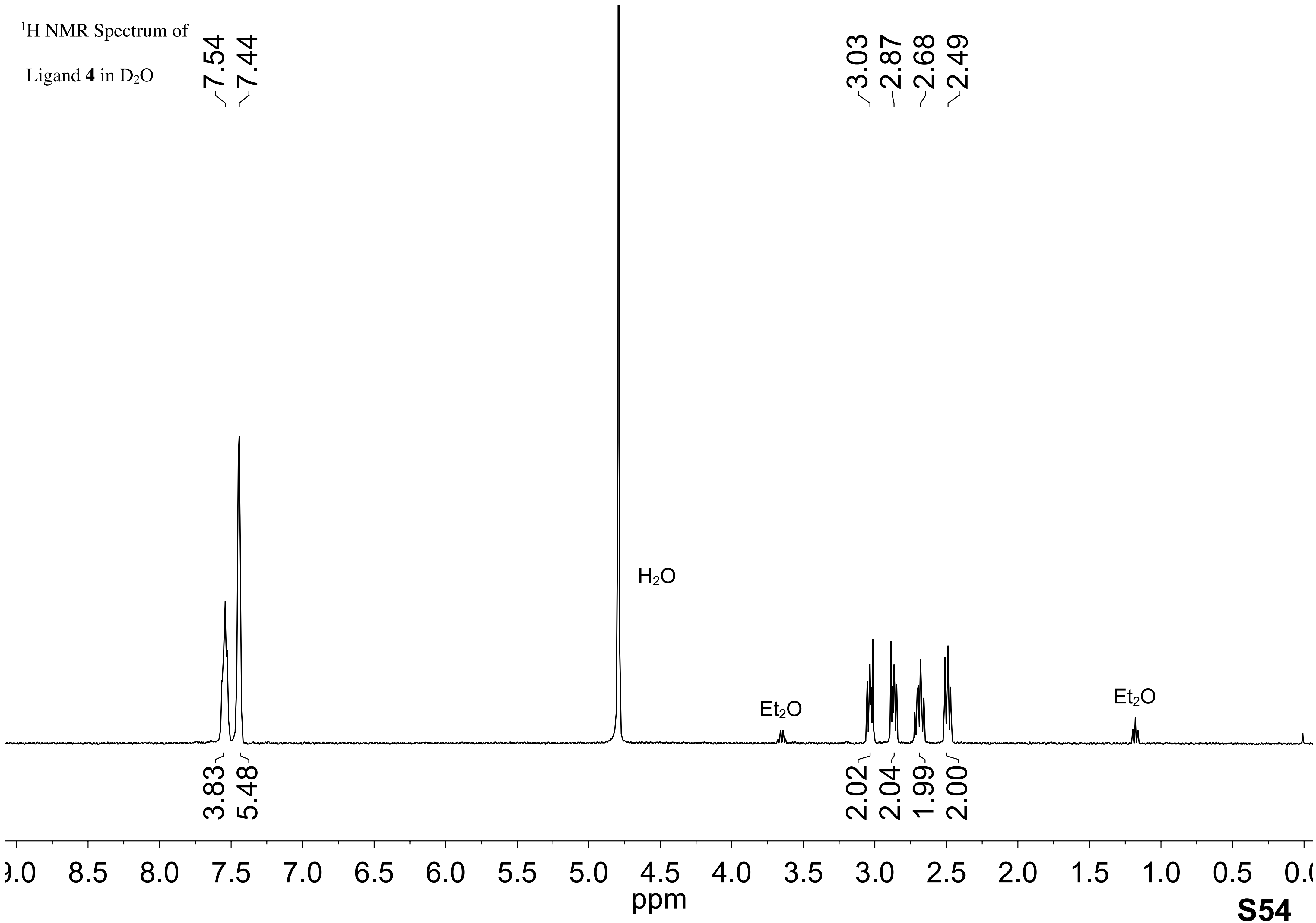

^{1}H NMR Spectrum of
Ligand **4** in D_2O
7.54
7.44
3.03
2.87
2.68
2.49
H_2O
Et_2O
Et_2O
3.83
5.48
2.02
2.04
1.99
2.00
9.0 8.5 8.0 7.5 7.0 6.5 6.0 5.5 5.0 4.5 4.0 3.5 3.0 2.5 2.0 1.5 1.0 0.5 0.0
ppm

³¹P{¹H} NMR Spectrum of

Semi-open **1** in DMSO-d_6

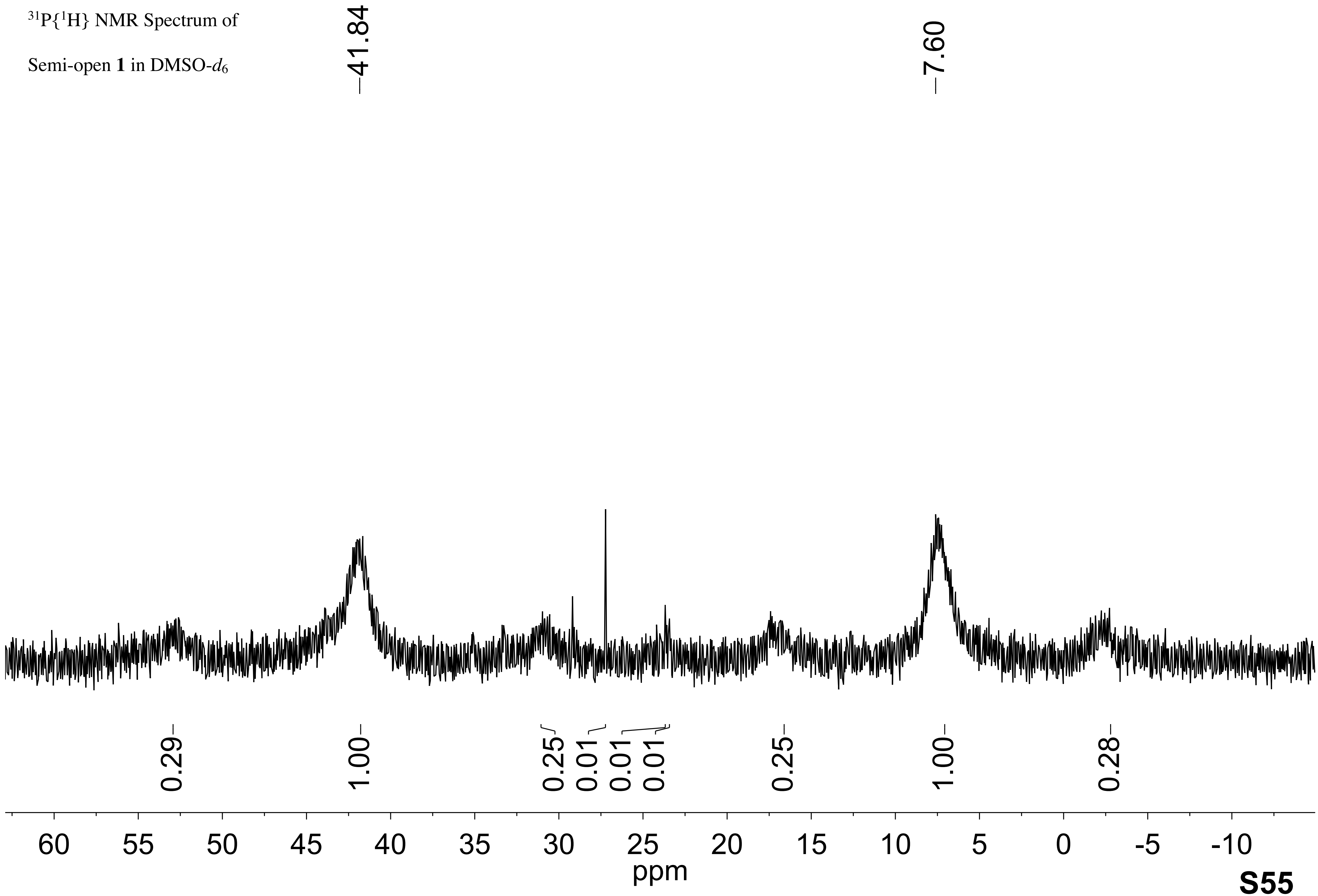

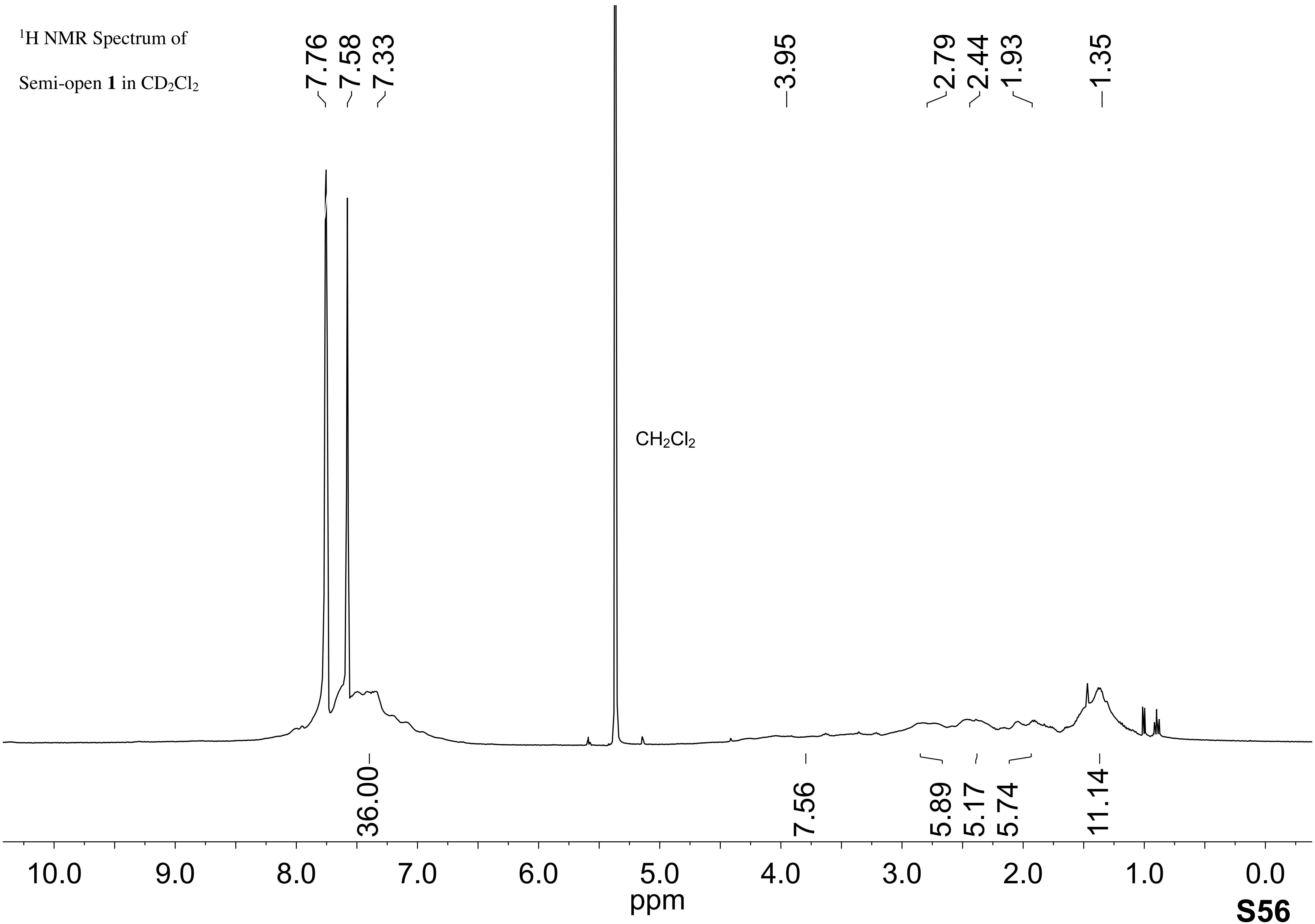
^{1}H NMR Spectrum of
Semi-open 1 in CD2Cl2
7.76
7.58
7.33
3.95
2.79
2.44
1.93
1.35
CH2Cl2
36.00
7.56
5.89
5.17
5.74
11.14
10.0
9.0
8.0
7.0
6.0
5.0
4.0
3.0
2.0
1.0
0.0
ppm

$^{19}F\{^1H\}$ NMR Spectrum of

Semi-open **1** in CD_2Cl_2

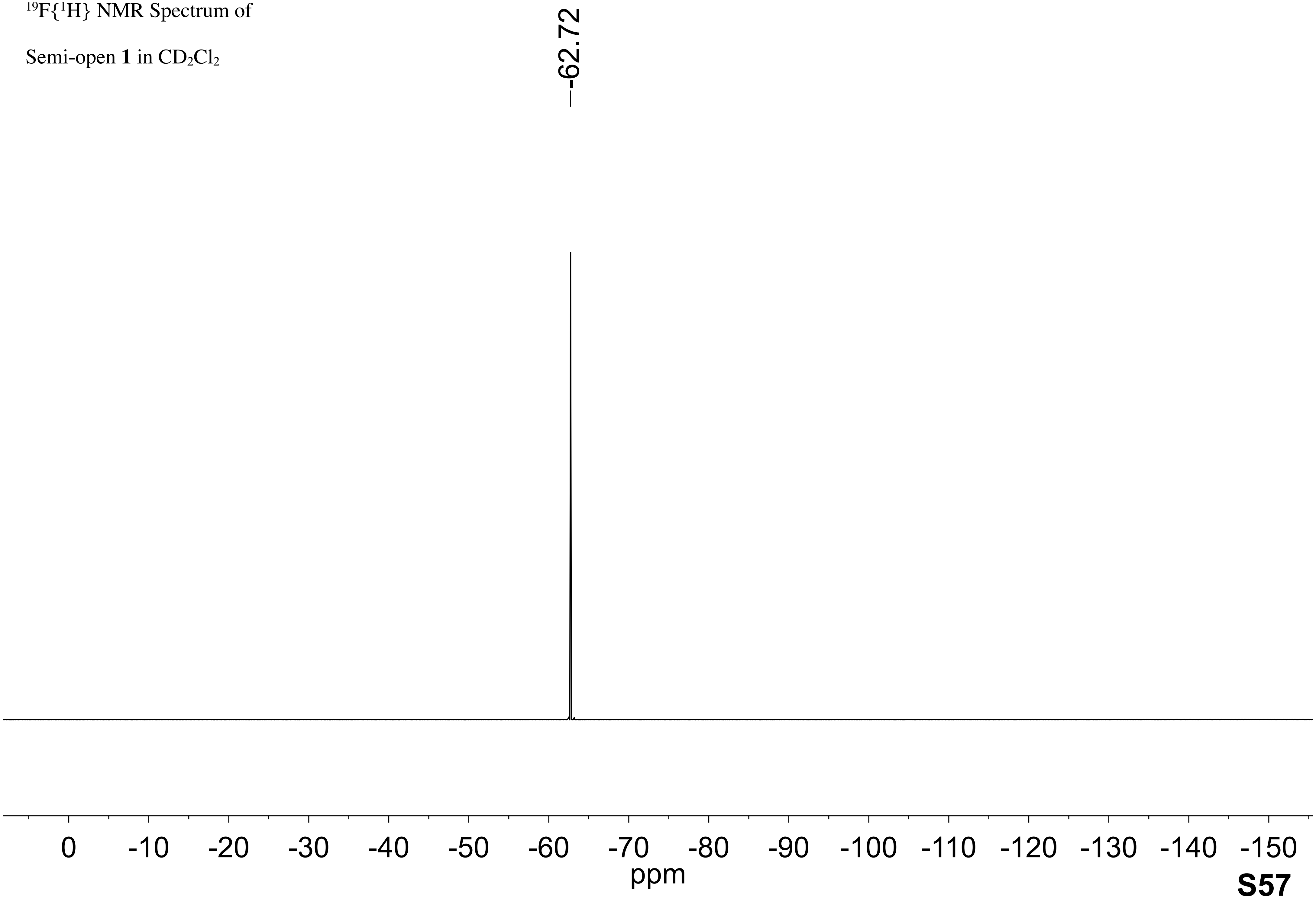

$^{31}P\{^{1}H\}$ NMR Spectrum of

Fully Closed **2** in DMSO-d_6

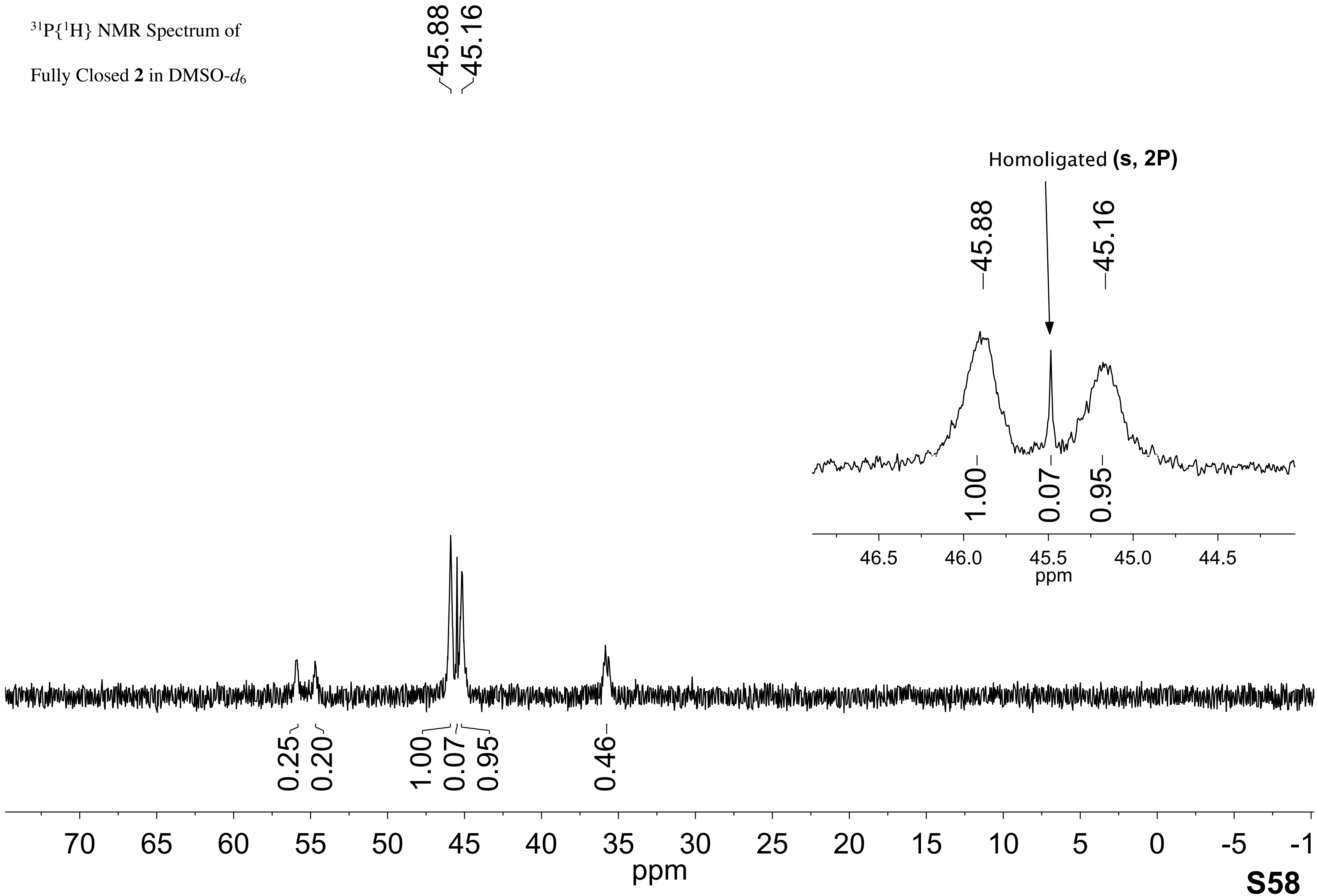

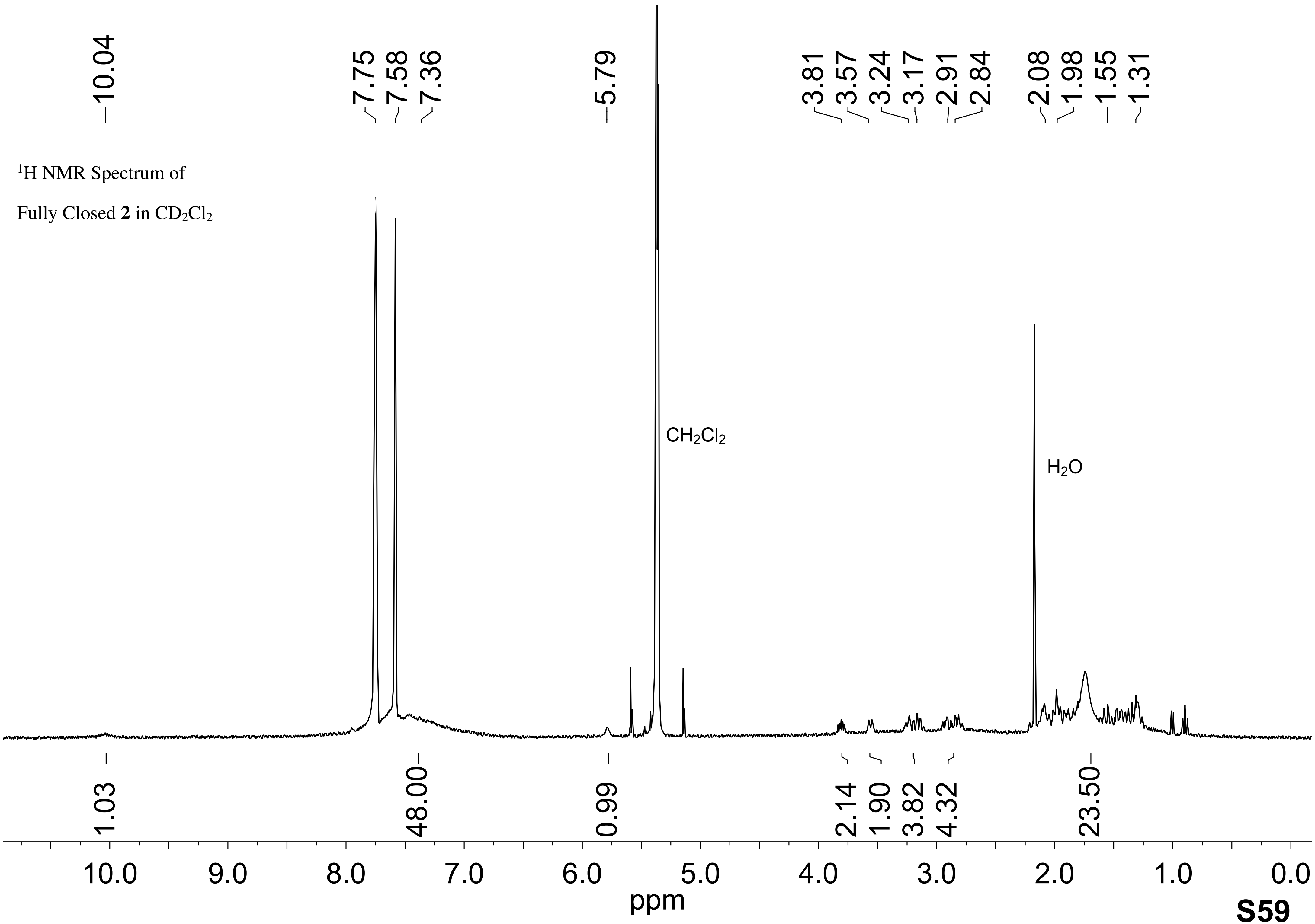
10.04
7.75
7.58
7.36
5.79
3.81
3.57
3.24
3.17
2.91
2.84
2.08
1.98
1.55
1.31
^{1}H NMR Spectrum of
Fully Closed 2 in CD_2Cl_2
CH_2Cl_2
H_2O
1.03
48.00
0.99
2.14
1.90
3.82
4.32
23.50
10.0
9.0
8.0
7.0
6.0
5.0
4.0
3.0
2.0
1.0
0.0
ppm

$^{19}F\{^{1}H\}$ NMR Spectrum of

Fully Closed **2** in CD_2Cl_2

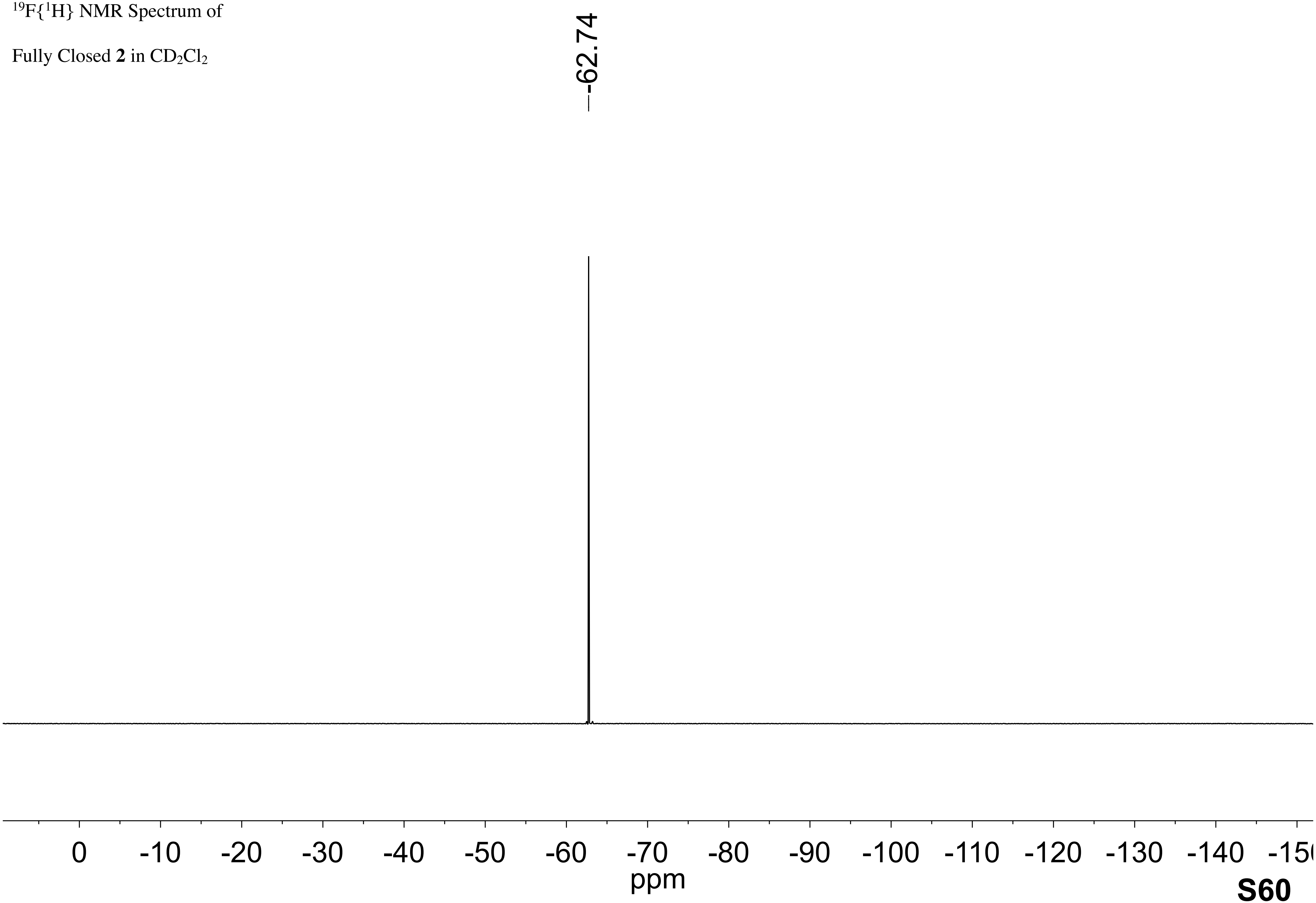

$^{31}P\{^1H\}$ NMR Spectrum of

Methyl 3–(2–diphenylphosphanylethylthio)propionate in CD_2Cl_2

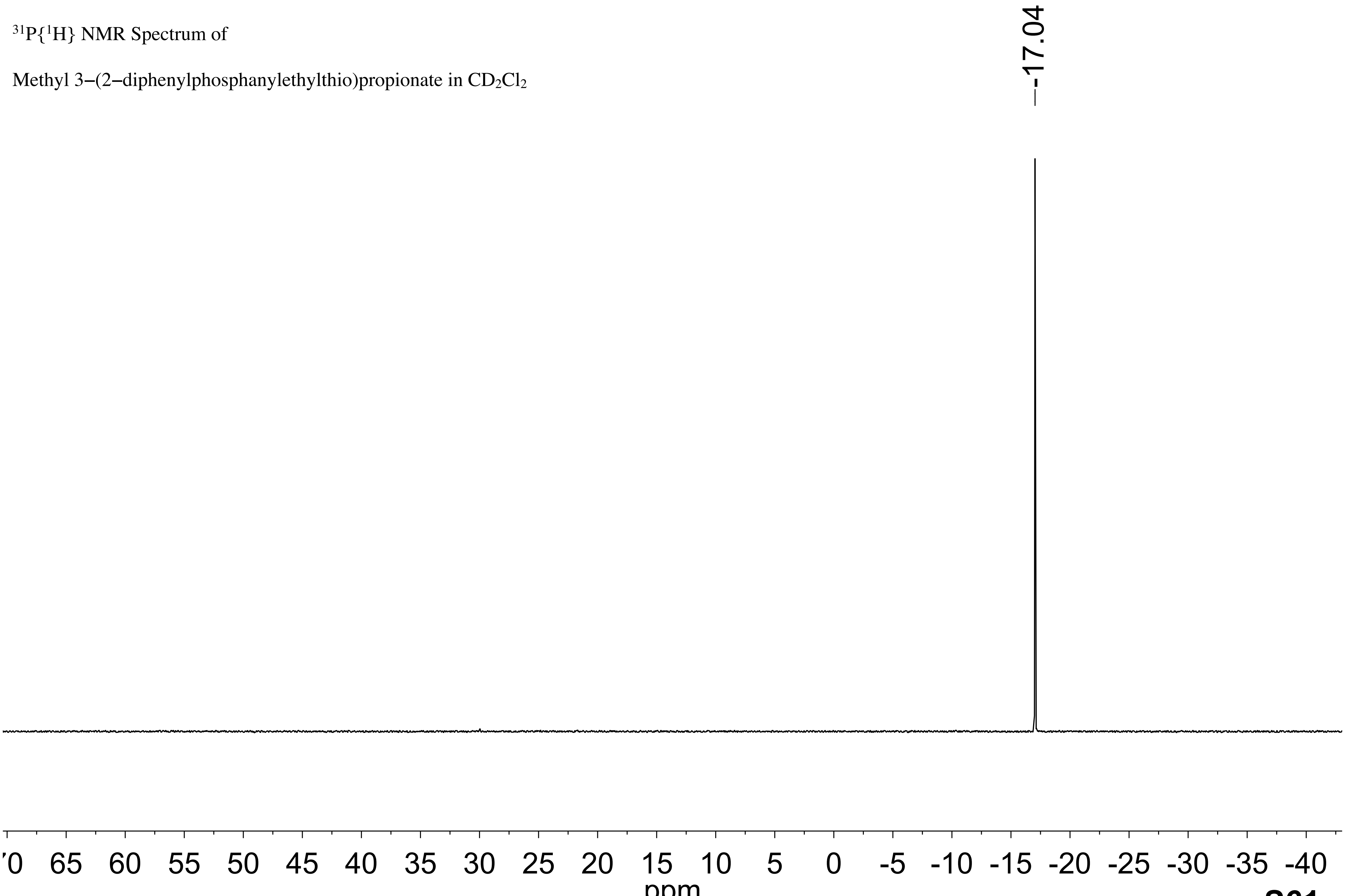

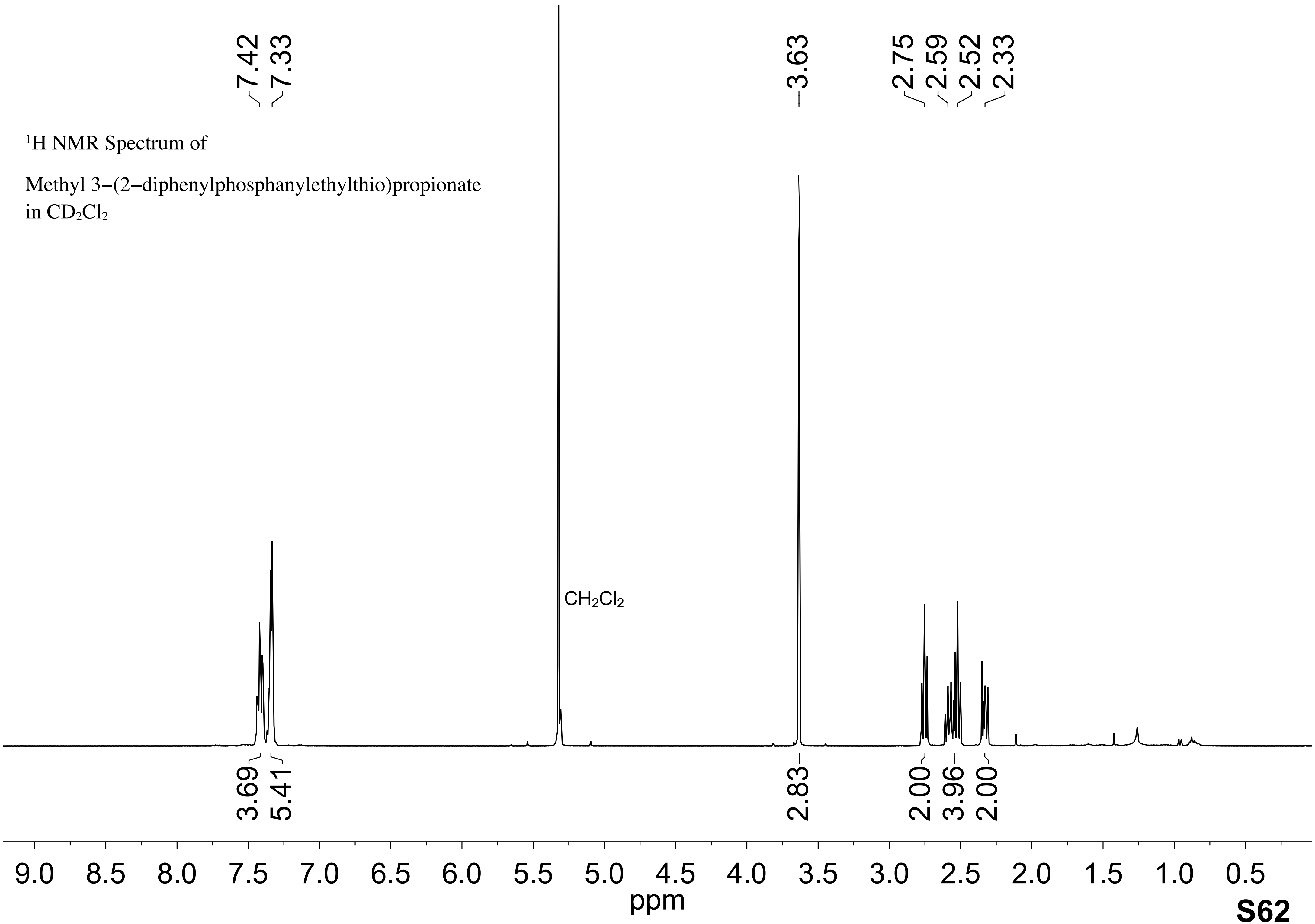

7.42
7.33
3.63
2.75
2.59
2.52
2.33
^{1}H NMR Spectrum of
Methyl 3−(2−diphenylphosphanylethylthio)propionate
in CD2Cl2
CH2Cl2
3.69
5.41
2.83
2.00
3.96
2.00
9.0 8.5 8.0 7.5 7.0 6.5 6.0 5.5 5.0 4.5 4.0 3.5 3.0 2.5 2.0 1.5 1.0 0.5
ppm

$^{31}P\{^1H\}$ NMR Spectrum of

Dimethyl 3−(2−diphenylphosphanylethylthio)propanamide in $CDCl_3$

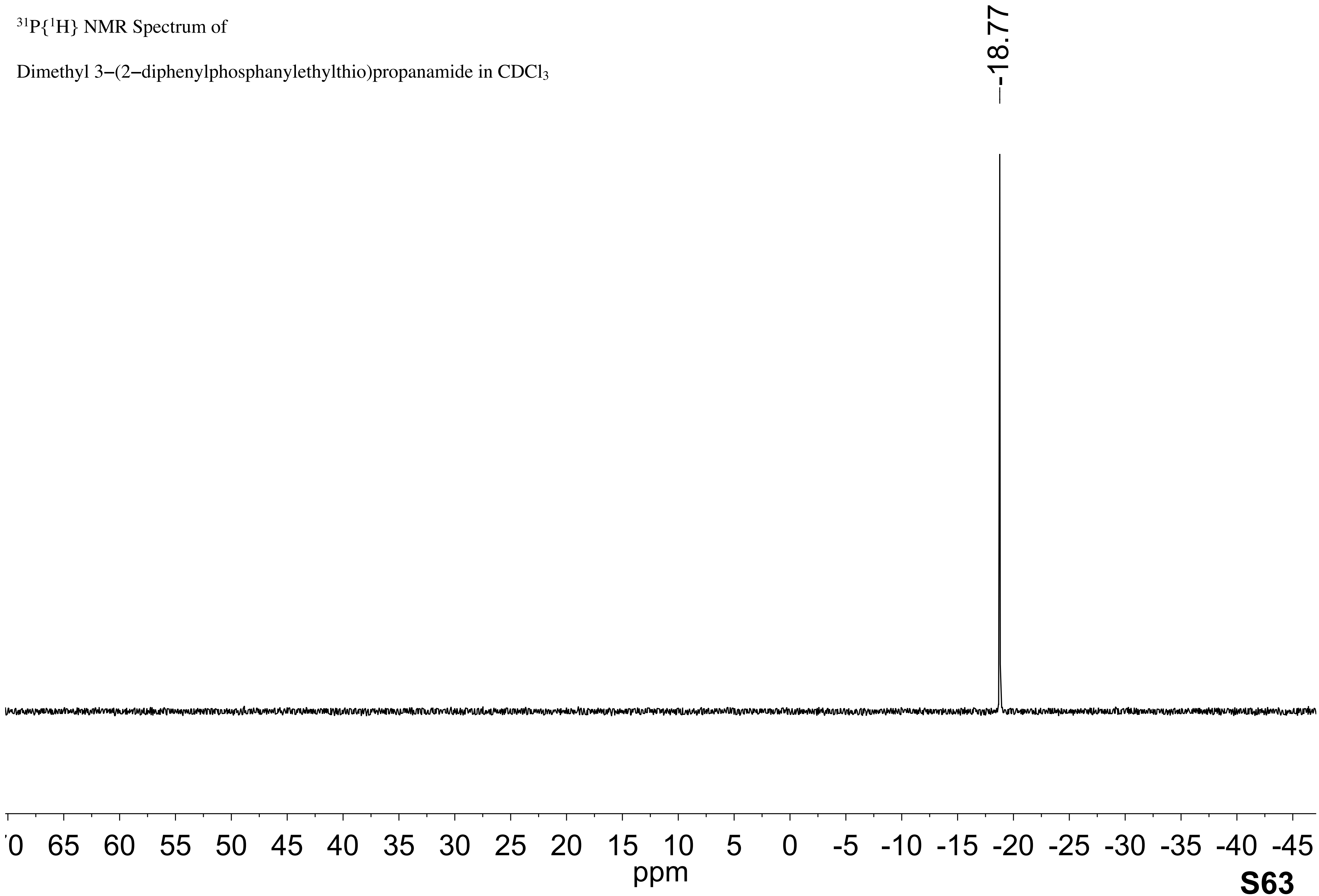

^{1}H NMR Spectrum of Dimethyl 3−(2−diphenylphosphanylethylthio)propanamide in $CDCl_3$

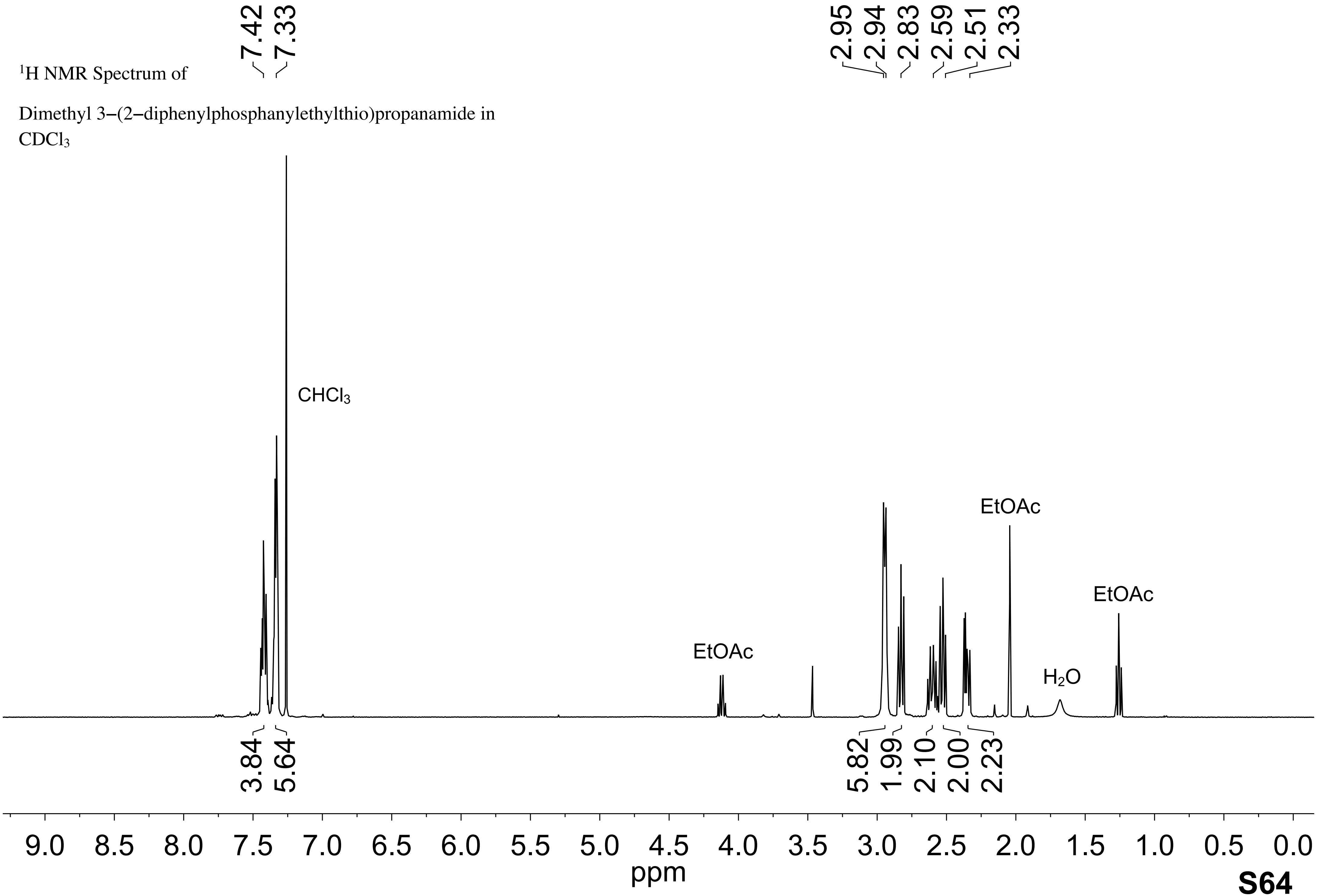

$^{31}P\{^{1}H\}$ NMR Spectrum of

Tetrabutylammonium
2−(2−diphenylphosphanylethylthio)ethanesulfonate in CD_2Cl_2

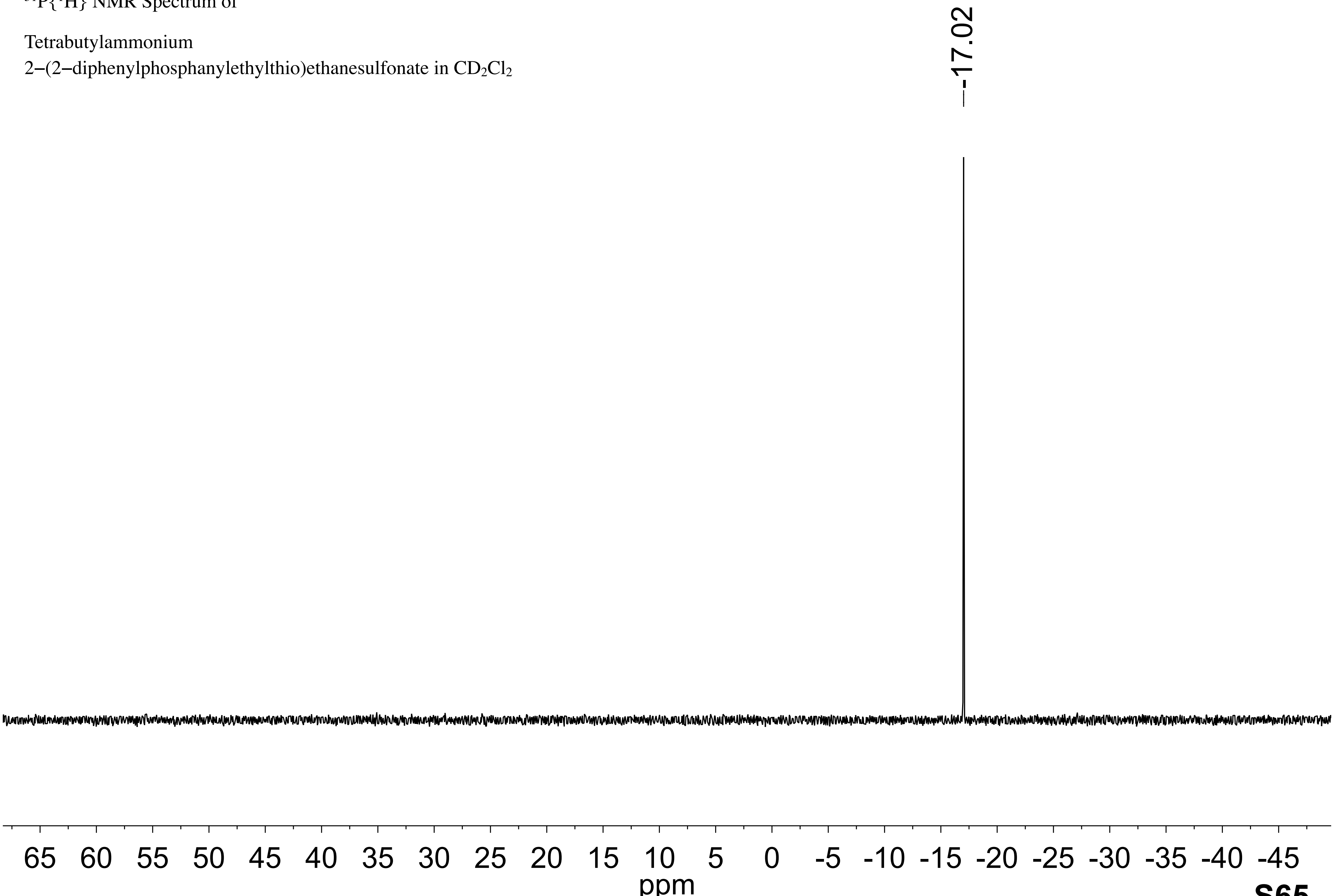

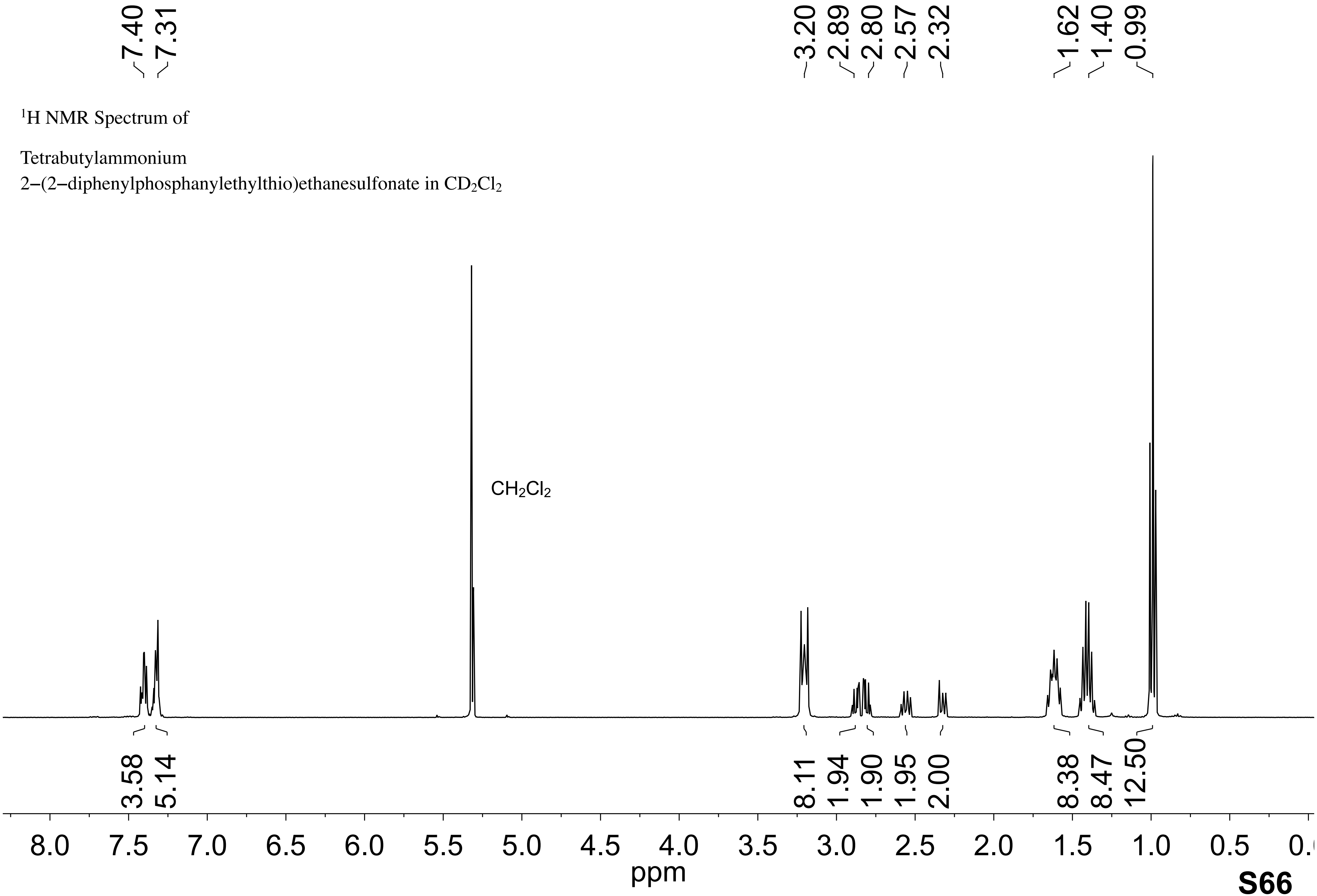
1H NMR Spectrum of
Tetrabutylammonium
2−(2−diphenylphosphanylethylthio)ethanesulfonate in CD2Cl2
CH2Cl2
7.40
7.31
3.20
2.89
2.80
2.57
2.32
1.62
1.40
0.99
3.58
5.14
8.11
1.94
1.90
1.95
2.00
8.38
8.47
12.50
8.0 7.5 7.0 6.5 6.0 5.5 5.0 4.5 4.0 3.5 3.0 2.5 2.0 1.5 1.0 0.5
ppm

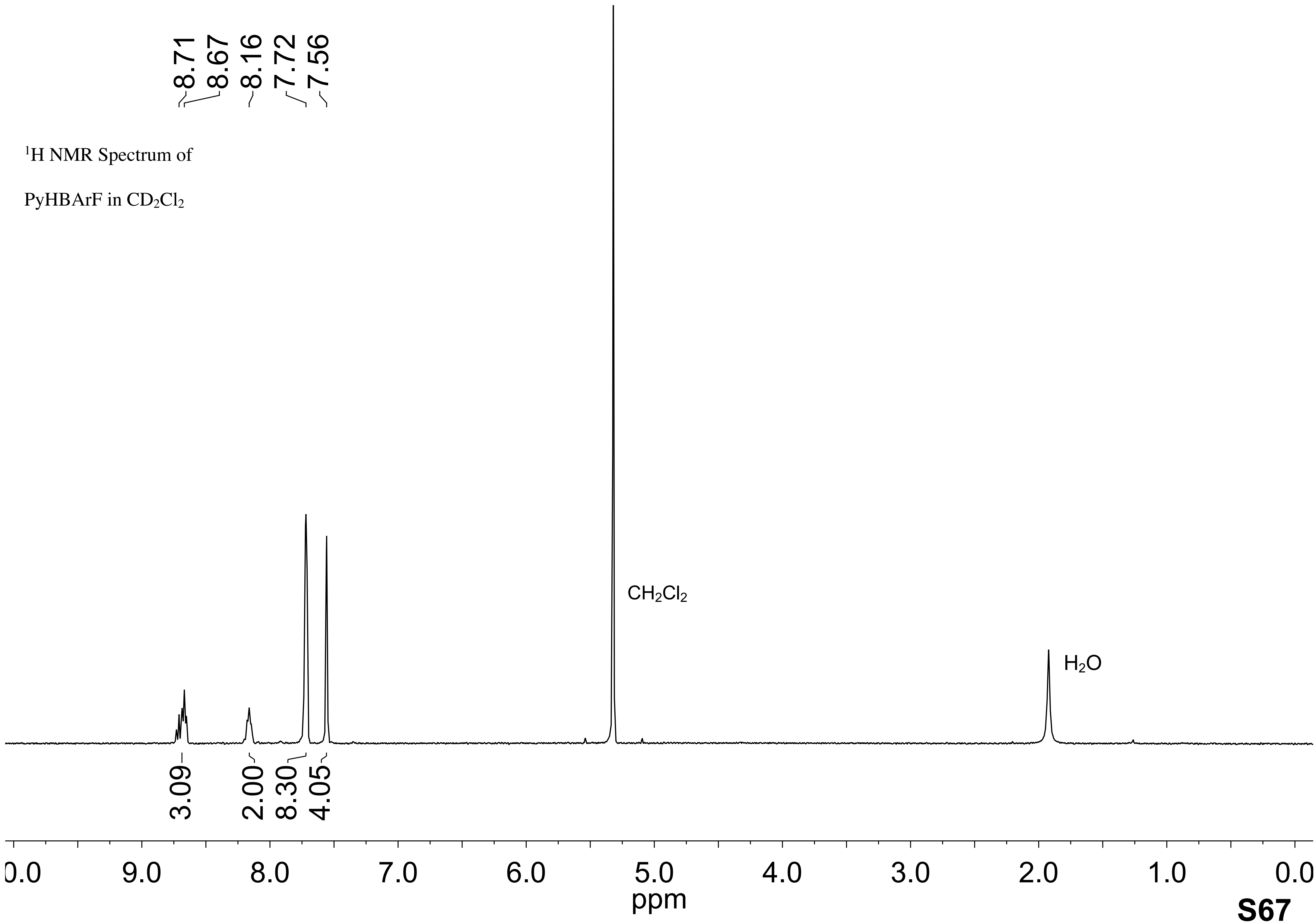
8.71
8.67
8.16
7.72
7.56
¹H NMR Spectrum of
PyHBArF in CD2Cl2
CH2Cl2
H2O
3.09
2.00
8.30
4.05
0.0
9.0
8.0
7.0
6.0
5.0
4.0
3.0
2.0
1.0
0.0
ppm

$^{31}P\{^{1}H\}$ NMR Spectrum of

Ligand **S2** in DMSO-d_6

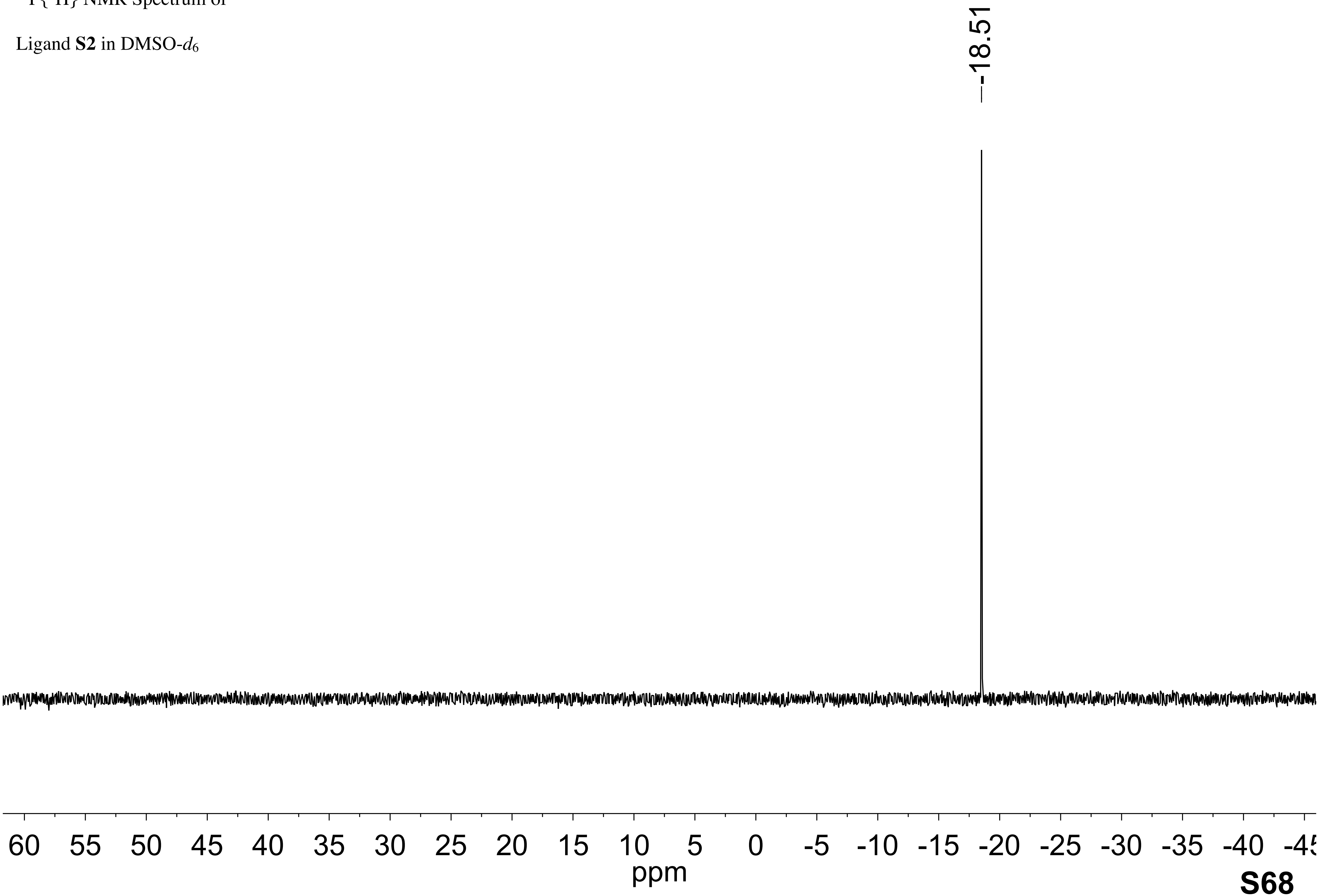

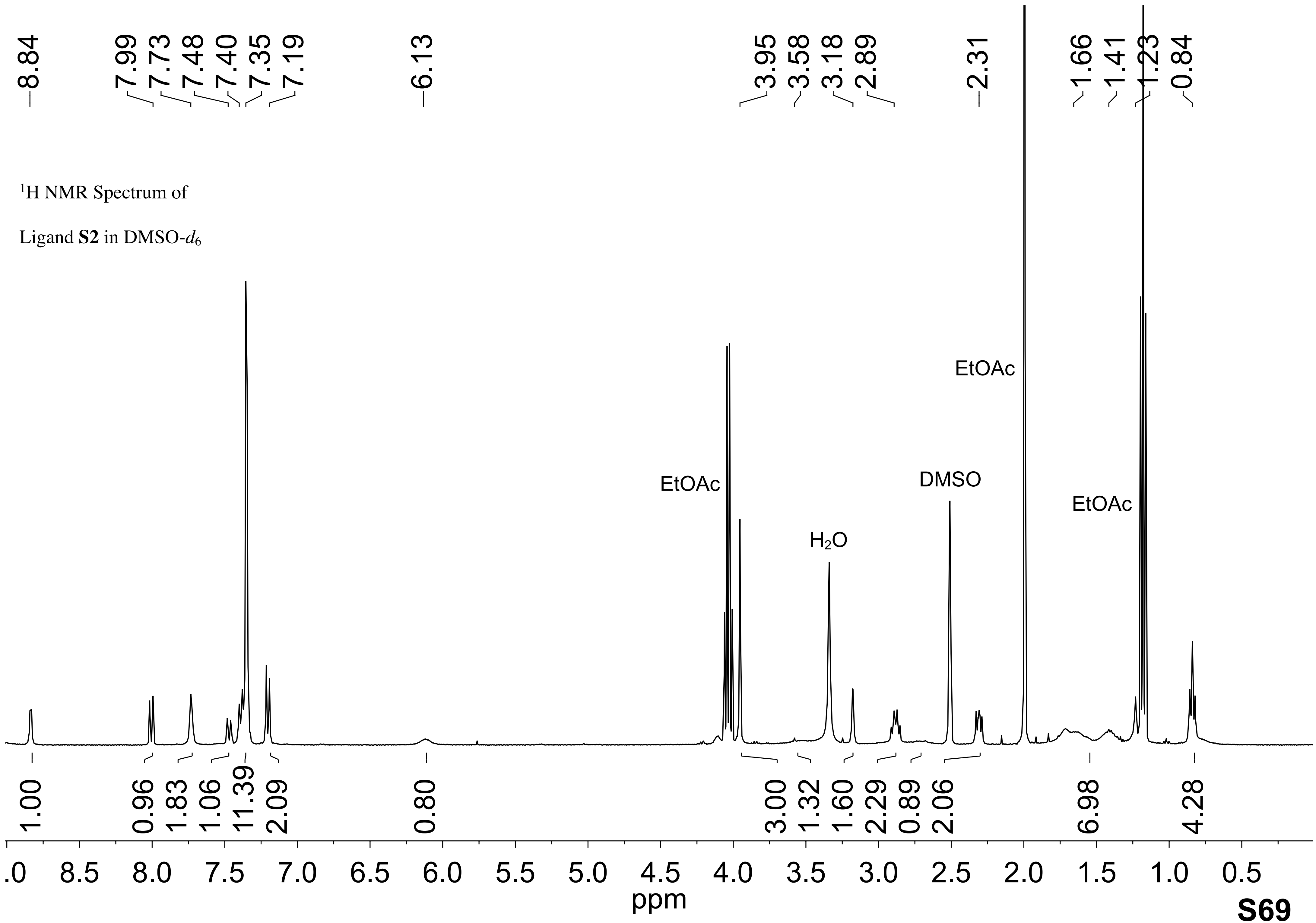

8.84
7.99
7.73
7.48
7.40
7.35
7.19
6.13
3.95
3.58
3.18
2.89
2.31
1.66
1.41
1.23
0.84
¹H NMR Spectrum of
Ligand S2 in DMSO-d6
EtOAc
H2O
DMSO
EtOAc
EtOAc
1.00
0.96
1.83
1.06
11.39
2.09
0.80
3.00
1.32
1.60
2.29
0.89
2.06
6.98
4.28
.0
8.5
8.0
7.5
7.0
6.5
6.0
5.5
5.0
4.5
4.0
3.5
3.0
2.5
2.0
1.5
1.0
0.5
ppm

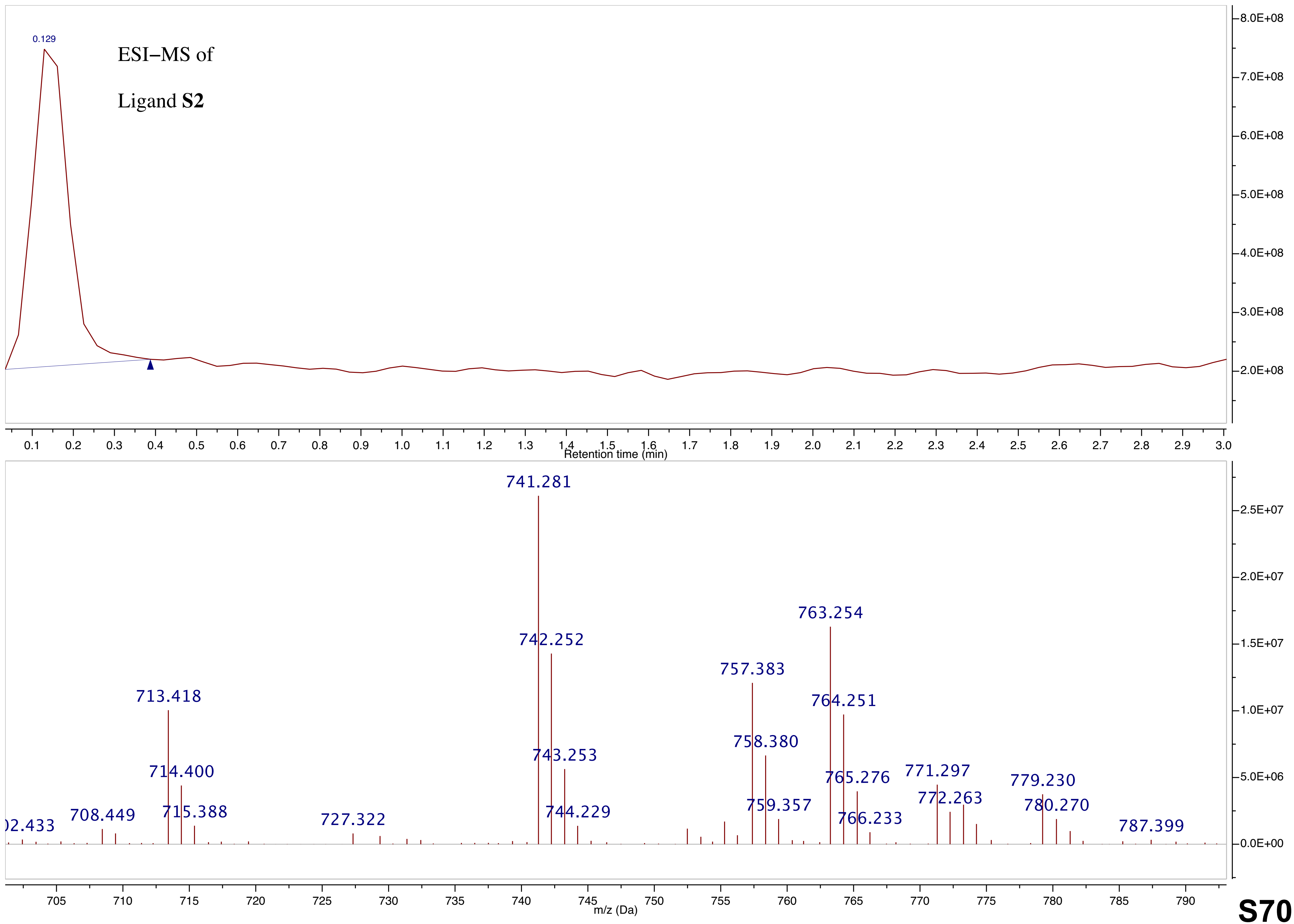
ESI–MS of
Ligand **S2**
0.129
Retention time (min)
741.281
742.252
743.253
744.229
763.254
764.251
765.276
766.233
757.383
758.380
759.357
713.418
714.400
715.388
708.449
727.322
771.297
772.263
779.230
780.270
787.399
m/z (Da)
